# Stabilization of Reactive Nitrene by Silylenes without Using a Reducing Metal

**DOI:** 10.1002/anie.202110456

**Published:** 2021-11-10

**Authors:** Yi Ding, Samir Kumar Sarkar, Mohd Nazish, Shahila Muhammed, Daniel Lüert, Paul Niklas Ruth, Christina M. Legendre, Regine Herbst‐Irmer, Pattiyil Parameswaran, Dietmar Stalke, Zhi Yang, Herbert W. Roesky

**Affiliations:** ^1^ Institut für Anorganische Chemie Universität Göttingen Tammannstrasse 4 37077 Göttingen Germany; ^2^ School of Chemistry and Chemical Engineering Beijing Institute of Technology Beijing 100081 P. R. China; ^3^ National Institute of Technology Calicut Kozhikode 673601 India

**Keywords:** amidinato ligands, hyperconjugative interactions, molecular orbitals, nitrene, silylenes

## Abstract

Herein, we report the stabilization of nitrene reagents as the source of a nitrogen atom to synthesize nitrogen‐incorporated R_1_LSi‐N←SiLR_2_ (**1**) [L=PhC(N*t*Bu)_2_; R_1_=NTMS_2_, R_2_=NTMS]. Compound **1** is synthesized by reacting LSi(I)‐Si^I^L with 3.1 equivalents of Me_3_SiN_3_ at low temperature to afford a triene‐like structural framework. Whereas the reaction of the LSi(I)‐Si^I^L with 2.1 equivalents of Me_3_SiN_3_ at room temperature produced silaimine **2** with a central four‐membered Si_2_N_2_ ring which is accompanied by a silylene LSi and a cleaved silylene fragment. **1** further reacts with AgOTf at room temperature to yield compound **3** which shows coordination of nitrene to silver with the triflate salt. The compounds **1** and **2** were fully characterized by NMR, mass spectrometry, and X‐ray crystallographic analysis. The quantum mechanical calculations reveal that compounds **1** and **2** have dicoordinated monovalent N atoms having two active lone pairs of electrons. These lone pairs are stabilized by hyperconjugative interactions.

## Graphical abstract

The uncharged univalent and reactive nitrene is captured and stabilized by silylenes. Two nitrene compounds are synthesized and fully characterized. The nitrene character was established by X‐ray analysis, first‐principles electronic structure studies using density functional theory, and natural bond orbital‐based bonding analysis.

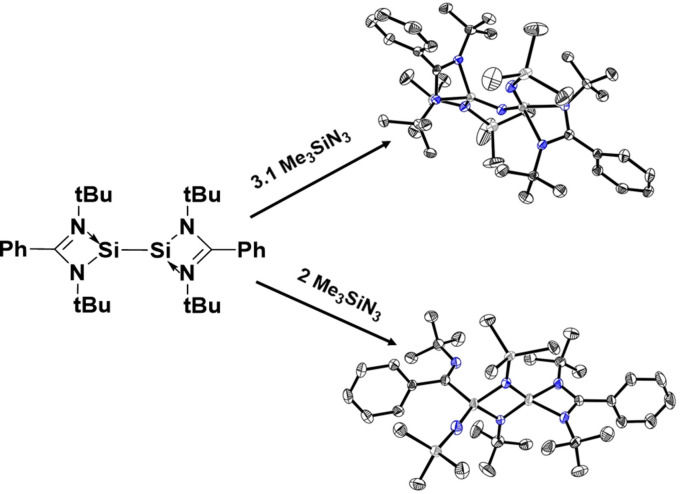

Nitrene is the nitrogen analogue of a carbene and has been used in many organic transformation reactions.^[1]^ The uncharged and univalent nitrogen atom in nitrene is highly reactive and was not isolated till now. The nitrene is generated in situ during reactions from its parent molecules such as azide or isocyanate by releasing N_2_ or CO, respectively.^[1]^ Whereas silylenes and bis(silylene) are the heavier analogues of carbenes and have attracted great interest over the last decade because of their easy synthesis.^[2]^ LSiCl and LSi‐SiL were extensively studied by different groups in recent years owing to their unique bonding nature and reactivities.^[2]^ We reported various reactions of the LSi‐SiL with benzophenone, benzil, diphenyl alkyne, phosphaalkyne or P_4_ afforded a four‐membered ring,^[3]^ a Si−Si bond connected two siladioxolene rings,^[4]^ the first room‐temperature stable 1,4‐disilabenzene,^[5]^ LSiC(Ad)SiPL ring or LSiPSiPL heteocycles.^[6]^ However, LSi‐SiL reacted with cyclooctatetraene and resulted in a colorless compound without cleavage of the Si−Si bond of the bis(silylene), but one of each Si−N(ligand) bond was opened.^[7]^ Treatment of the LSi‐SiL with AlH_3_⋅NEtMe_2_ afforded LAl(SiH_2_SiH_2_)_2_AlL, where two silicon atoms of cyclohexasilane were replaced by two aluminum atoms.^[8]^ Moreover, the reaction of LSi‐SiL with AlMe_3_ yielded a silylene‐alane adduct LSi(AlMe_3_)‐Si(AlMe_3_)L.^[8]^ C.‐W. So et al. found that bromine cleaved the Si−Si bond to afford novel monomeric bromosilylene LSiBr.^[9]^ LSi‐SiL reacted with PhC≡CH or PhC≡*CPh* to produce *cis*‐LSi[C(Ph)=C(H)]SiL or delocalized biradicaloid LSi(*μ*_2_‐C_2_Ph_2_)_2_SiL.^[10]^ The reaction of the LSi‐SiL with ArN=C=NAr afforded [LSi(*μ*‐CNAr)_2_SiL] and [LSi‐(=NAr)‐SiL].^[11]^ LSi‐SiL reacted with [4‐NMe_2_C_5_H_4_NSiMe_3_]OTf and DMAP to form [LSi(DMAP)]OTf. The latter reacted with LSi‐SiL, KHB(*i*Bu)_3_ or S_8_ to yield [LSi(DMAP)]OTf that exhibited both electrophilic and nucleophilic characters.[^12^, ^13^] P. Roesky et al. reported the reaction of LSi‐SiL with LSiCl and NaBPh_4_ to afford a symmetrically tetrasilacyclobutadiene dication salt of composition [(LSi)_4_](BPh_4_)_2_.^[14]^ Recently, Driess et al. described the bis(silylene)‐stabilized nitrogen cations.^[15]^ Very recently, we have reported a neutral 2π‐aromatic three‐membered disilaborirane.^[16]^ Inspired by the high reactivity of bis‐silylene LSi‐SiL, we report two reactions between LSi‐SiL with different equivalents of Me_3_SiN_3_ which yielded nitrogen atom incorporation of a LSi‐N←SiL unit of **1** by migrating the trimethylsilyl group from nitrene (Me_3_SiN:) and a central four‐membered Si_2_N_2_ ring system (**2**).

The LSi‐SiL reacted with 3.1 equivalents of Me_3_SiN_3_ in toluene at −78 °C to afford L(N(SiMe_3_)_2_)SiNSi(L)NSiMe_3_ (**1**) with a toluene molecule (Scheme 1). Compound **1** was obtained as a colorless crystalline solid in 66 % yield and showed good solubility in different organic solvents such as benzene, toluene, diethyl ether, and THF. Compound **1** is fully characterized by ^1^H, ^13^C, ^29^Si NMR and HRMS techniques. The ^1^H NMR spectrum of **1** showed three singlets at *δ*=0.48, 0.65, and 0.73 ppm for the trimethylsilyl groups, two singlets at *δ*=1.38 and 1.42 ppm for the ^t^Bu substituents, and four broad resonances at *δ*=6.93 −7.52 ppm attributed to phenyl protons. We have calculated ^29^Si NMR spectra of compound **1** at the M06/PCM/def2‐TZVPP//BP86/D3‐BJ/def2‐TZVPP level of theory and have assigned the chemical shift values for each Si atom.^[18]^ The calculated values (Table S13) are in agreement with the experimental NMR chemical shift values. The chemical shift values of silyl silicon centers in compound **1** viz., Si3 (*δ*=−0.93 (exp); *δ*=1.91 (calc)), Si4 (*δ*=4.25 (exp); *δ*=3.45 (calc)) and Si5 (*δ*=−27.28 (exp); *δ*=−24.01 (calc)) are less shielded as compared to the other two Si centers viz., Si1 (*δ*=−62.95 (exp); *δ*=−61.78 (calc)) and Si2 (*δ*=−57.20 (exp); *δ*=−56.64 (calc)). Single crystals were obtained from the toluene solution of **1** by storing at −32 °C in a freezer after three days. X‐ray structural analysis reveals that compound **1** crystallizes in the monoclinic space group *P* $\bar{1}$ .

**Scheme 1:**
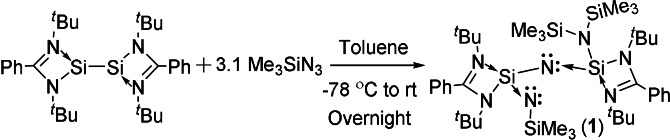
Synthetic route for the preparation of **1**.

Our previous studies revealed the reaction of LSiN(SiMe_3_)_2_ with AdN_3_ (Ad=adamantyl) to result in the formation of a silaimine with a terminal SiMe_3_ group through 1,3‐silyl migration of one SiMe_3_ group to form L(N(Ad)SiMe_3_)SiN(SiMe_3_).^[17]^ The detailed mechanism of the reaction is still unclear. However, the same type of 1,3‐silyl migration occurs during the present reaction from LSi‐SiL to **1**. We propose that an unstable N(SiMe_3_) group bridging disilaimine forms as an intermediate under the elimination of dinitrogen. Subsequently, there is a 1,3‐migration of the SiMe_3_ group from bridging nitrogen atom to another terminal nitrogen.

The structure of **1** shows a central coordinate nitrogen atom bridging two benzamidinate‐coordinated silicon atoms Si1 and Si2 (Figure 1). In addition, the silicon atoms are either coordinated by a trimethylsilyl imido group (Si1−N3 1.6126(18) Å) or a bis(trimethylsilyl)amido group (Si2−N7 1.7212(18) Å), respectively. The first distance is close to the central Si2−N4 bond length 1.6115(19) Å, while the second Si1−N4 distance of 1.6522(18) Å is noticeably longer. The shorter distances, however, are within the typical range for Si=N double bonds found in silaimines (1.545–1.6113 Å).[^2^, ^11^, ^17^] It is most interesting to note that the five atoms N3, Si1, N4, Si2, and N7 are almost sharing a plane to form the main backbone of **1**.

**Figure 1:**
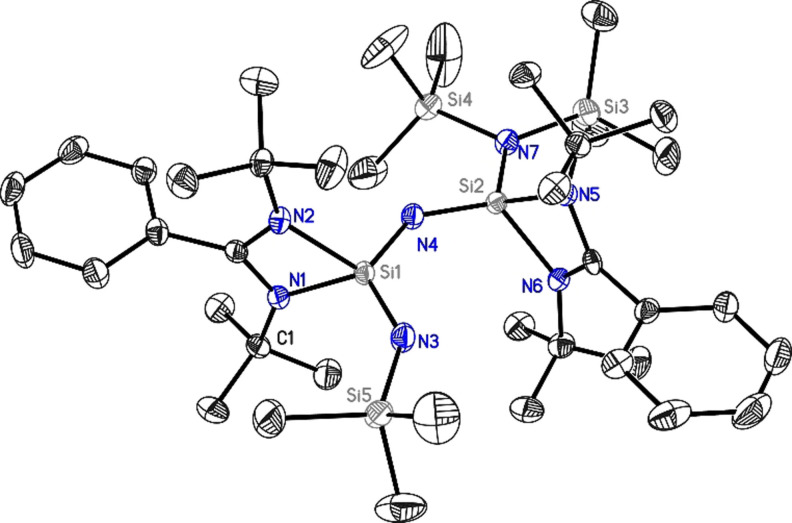
Molecular structure of **1**. Anisotropic displacement parameters are depicted at the 50 % probability level. The hydrogen atoms are omitted for clarity. Si1–N1: 1.8841(18) Å, Si1–N2: 1.8774(18) Å, Si1–N3: 1.6126(18) Å, Si1–N4: 1.6522(18) Å, Si2–N4: 1.6115(19) Å, Si2–N5: 1.8294(18) Å, Si2–N6: 1.8323(18) Å, Si2–N7: 1.7212(18) Å.

Treatment of the LSi‐SiL with 2 equivalents of Me_3_SiN_3_ in toluene at room temperature, yielded compound **2** (Scheme 2). The ^1^H NMR spectrum of **2** showed two singlets at *δ*=0.36 and 0.51 ppm for the trimethylsilyl groups, four sharp singlets at *δ*=1.10, 1.11, 1.30, and 1.59 ppm for the ^t^Bu substituents. It also displayed six broad resonances at *δ*=6.68–7.52 ppm attributed to phenyl protons. The calculated (M06/PCM/def2‐TZVPP//BP86/D3‐BJ/def2‐TZVPP) and experimental ^29^Si NMR chemical shifts values of compound **2** (Table S14) indicates that silyl Si centers viz., Si3 (*δ*=−3.67 (exp); *δ*=−0.83 (calc)) and Si4 (*δ*=−28.48 (exp); *δ*=−24.87 (calc)) are less shielded as compared to the other two Si centers viz., Si1 (*δ*=−50.81 (exp); *δ*=−56.81 (calc)) and Si2 (*δ*=−70.94 (exp); *δ*=−80.0 (calc)) The single crystal for X‐ray analysis was grown from the concentrated toluene solvent of compound **2** at −30 °C in a fridge after five days.

**Scheme 2:**
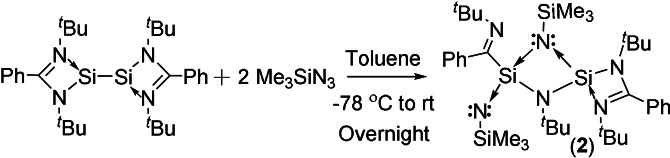
Synthetic route for the preparation of **2**.

The structure of compound **2** is probably formed after ring‐opening, followed by ligand scrambling and rearrangement (Figure 2). The transition state compound from ligand scrambling might have shown three *spiro* Si‐connected four‐membered rings, with an overwhelming ring tension. Hence, one C−N bond of the amidino ligands is cleaved and the related silicon atom bonds to the carbon rather than to the nitrogen atom of the ligand to give **2**.

**Figure 2:**
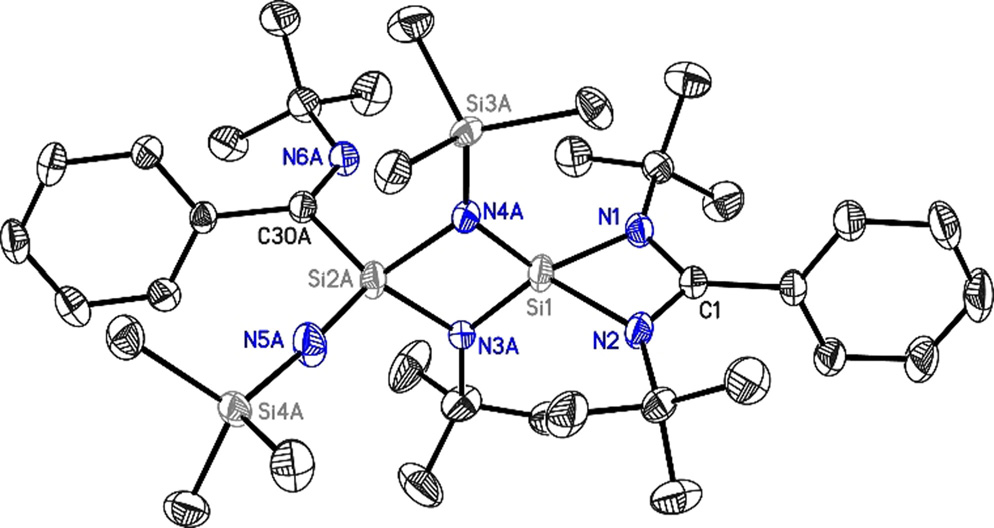
Molecular structure of **2**. Anisotropic displacement parameters are depicted at the 50 % probability level. The hydrogen atoms as well as the second position are omitted for clarity. Si1–N1: 1.8051(19) Å, Si1–N2: 1.8114(19) Å, Si2A/B‐N5A/B: 1.598(4) Å/1.589(11) Å, where A and B stand for severely disordered residues.

The X‐ray crystallography of **2** clearly shows that two Si atoms are bridged by one N(SiMe_3_) and one N(*t*Bu) group each to give a central four‐membered N_2_Si_2_ ring where the Si atoms of the ring are four‐coordinate. The amidinato silicon four‐membered ring is arranged perpendicular to this central N_2_Si_2_ four‐membered ring. Interestingly, atom Si1 coordinates to the amidinato ligand in a *N*,*N*′‐chelating fashion, whereas atom Si2A coordinates to the central carbon atom C30A of the scrambled amidinato ligand. The coordination sphere of Si2A is completed by an additional NSiMe_3_ group. The C−N bond lengths in **2** (C1−N1: 1.341(3) Å; C1−N2: 1.340(3) Å) are approximately the same as in LSiCl^[2a]^ (1.333(2) Å) and LSi−SiL^[2b]^ (1.331(6) Å). These geometries show considerable delocalization throughout the NCN backbone of the ligand. However, the Si1−N1 (1.8051(19) Å) and Si1−N2 bond lengths (1.8114(19) Å) are slightly shorter than the Si−N_amidinato_ bond length in the starting material LSiCl^[2a]^ and LSi‐SiL.^[2b]^ As the bridging groups are equally disordered between two positions, the bond lengths and angles within these groups should be considered with caution. An additional independent disorder is present at the atoms labeled Si2A/B to C36A/B (see Supporting Information, Figure S16). This is less pronounced and allows the determination of the C30A/B−N6A/B bond lengths to be 1.287(4) Å and 1.266(11) Å implying a C=N double bond. The distance between Si1 and Si2A is 2.4855(9) Å, while the distance between Si1 and Si2B is 2.482(3) Å, which is in both cases longer than the Si−Si bond in LSi(I)−Si^I^L (2.413 Å). This suggests that there is no bond present between these two silicon atoms in **2**.^[2b]^ The N1‐Si1‐N2 angle (72.90(7)°) in **2** is larger than that in LSiCl^[2a]^ and LSi‐SiL^[2b]^ (68.35(8)° and 69.52(18)°), while the N1‐C1‐N2 angle (106.50(13)°) in **2** is similar to that in LSiCl^[2a]^ and LSi‐SiL^[2b]^ (105.94(18)° and 105.2(3)°). The central Si_2_N_2_ four‐membered ring is close to ideal planarity.

Treatment of **1** with one molar equivalent of AgOTf in toluene at room temperature yield the respective nitrene silver adduct **3** (Scheme S1). As expected for **3**, the coordination of silver to the nitrene led to a downfield and upfield shift in the respective ^29^Si NMR resonance (**3**: *δ*=6.19, 0.43, −10.89, −63.59, −65.05 ppm) in comparison with those of **1** (*δ*=4.25, −0.93, −27.28, −57.20, −62.95 ppm, see Supporting Information, Experimental Section).[^17b^, ^17c^]

Quantum mechanical calculations at the M06/def2‐TZVPP//BP86‐D3BJ/def2‐TZVPP are performed to understand the electronic structure and bonding in **1**, and **2**. The benzamidinato‐stabilized bis(silylene) (Figure S17‐c) possesses two pyramidal silicon centers owing to the presence of a σ‐type lone pair on each silicon, having high s‐character (70.9 % s and 28.9 % p). The reaction of bis(silylene) with Me_3_SiN_3_ results in compounds **1** and **2**. The reaction energies for the formation of **1** (Scheme 1) are calculated to be highly exothermic (−276.9 kcal mol^−1^) and exergonic (−260.6 kcal mol^−1^). The Si2−N_(amidinato)_ bonds are shortened (1.845 Å and 1.849 Å, Figure S17‐a) in **1** as compared to those in bis(silylene) (1.905 Å and 1.913 Å, Figure S17‐c). The longer Si2−N bond length in bis(silylene) can be attributed to the inter‐electronic repulsion arising from the lone pair of electrons on the silicon atoms. Compound **1** has two dicoordinated N‐centers viz., N3 and N4. Both N3 and N4 are coordinated to tricoordinated Si centers and have bent geometry. The Si‐N3‐Si and Si‐N4‐Si bond angles are 135.7 and 130.6 degrees, respectively. The bonds between Si and dicoordinated nitrogens viz., Si−N3 and Si−N4 are much shorter than the bonds between Si and tricoordinated nitrogens. One of the Si−N3 and Si−N4 bonds is shorter than the others and is within the range of experimentally reported delocalized Si=N bonds.^[17]^

The molecular orbital analysis (Figure 3‐a) shows that HOMO and HOMO−1 represent the π and σ lone‐pair orbitals on N3, and HOMO−2 and HOMO−3 correspond to π and σ lone pair orbitals on N4. However, the lone pairs on N3 and N4 are delocalized towards the Si1 and Si2 centers. The lone pair on N3 is delocalized towards the SiMe_3_ group as well. The NBO analysis indicates that these two dicoordinated nitrogen centers are monovalent, possessing two lone pairs. The lone pair occupancies are 1.80 and 1.76 for N3 and 1.81 and 1.75 for N4 (Table S4). Hence, it is anticipated that the hyperconjugative donation of the lone pair on dicoordinated N to Si−N_amidinate_ σ* and Si−C σ* orbitals make Si2−N4 and Si1−N3 bonds shorter. The energy calculated for these hyperconjugative interactions using the second‐order perturbative method is significant (Table 1). It is noteworthy that the hyperconjugative donation from π‐type lone pairs is stronger than the hyperconjugative donation from σ‐type lone pairs. The hyperconjugative donation from both lone pair orbitals on N4 to S2−N σ* orbitals (20.81 kcal mol^−1^ and 23.81 kcal mol^−1^ for LP(σ) and LP(π) respectively) is higher in energy as compared to the donation to S1−N σ* orbitals (13.92 kcal mol^−1^ 21.01 kcal mol^−1^ for LP(σ) and LP(π), respectively). Similarly, the hyperconjugative donation from both lone pair orbitals on N3 to S1−N σ* orbitals (22.9 kcal mol^−1^ and 26.1 kcal mol^−1^ for LP(σ) and LP(π) respectively) is higher in energy as compared to the donation to S5−C σ* orbitals (14.43 kcal mol^−1^ and 16.46 kcal mol^−1^ for LP(σ) and LP(π), respectively). This is corroborated well with the geometrical data viz., the Si1−N3 and Si2−N4 bonds are shorter than the Si5−N3 and Si1−N4 bonds. Even after these strong hyperconjugative interactions, the charge on the Si1 (2.29 e) and Si2 (2.36 e) centers are highly positive, and those of N3 (−1.81 e) and N4 (−1.83 e) are highly negative (Table S7). It can be attributed to highly polarized Si−N bonds. For example, the Si1−N3 and Si2−N4 sigma bonding orbitals have 81.8 % and 81.9 % contributions from N3 and N4 atoms, respectively, making the bond polarized towards the nitrogen centers.

**Figure 3:**
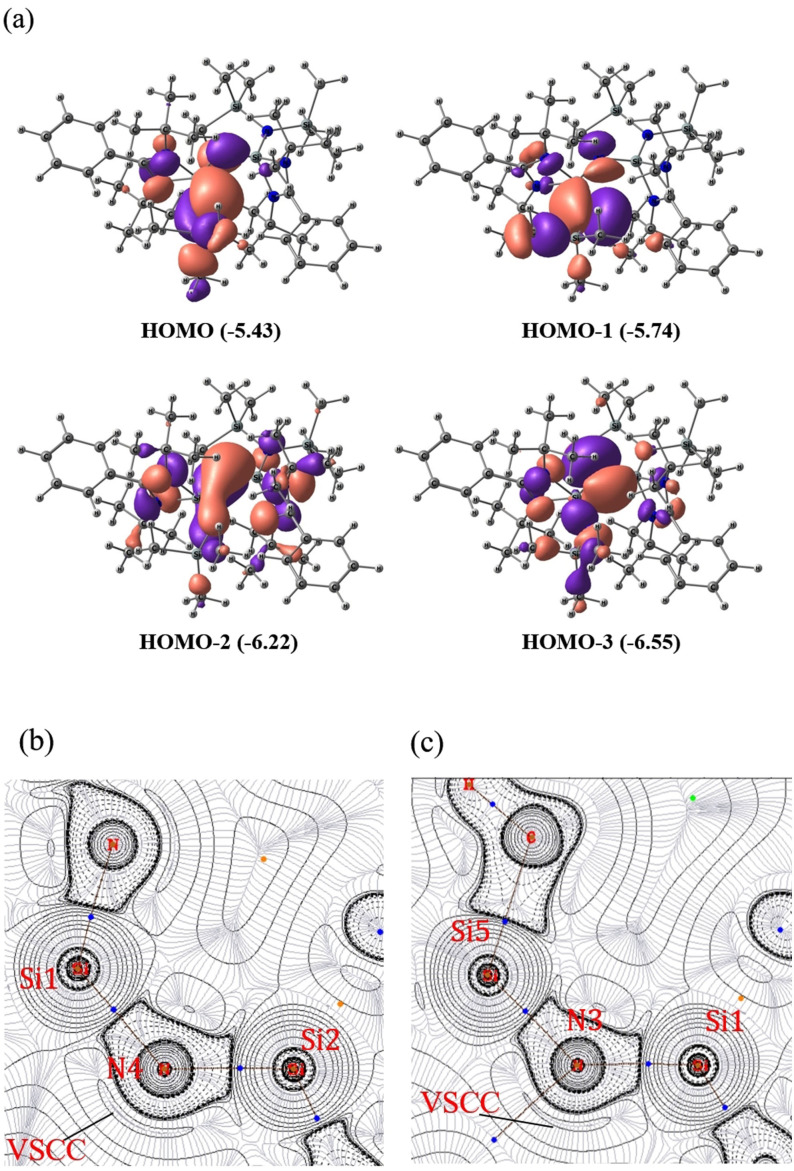
a) Four high lying molecular orbitals correspond to the delocalized lone pairs on N3 and N4, calculated at the M06/def2‐TZVPP//BP86‐D3BJ/def2‐TZVPP level of theory. The surfaces are plotted at the iso‐surface value of 0.03. The energy values given in the parentheses are in eV. b,c) The Laplacian of the electron density plotted in the plane of Si1‐N4‐Si2 and Si1‐N3‐Si5 respectively of **1**. QTAIM analysis was done on the wavefunction generated at M06/def2‐TZVPP//BP86‐D3BJ/def2‐TZVPP level of theory. Valence shell charge concentration (VSCC) is marked. The atom numbering is based on Figure S17‐a.

**Table 1:** Hyperconjugative stabilization energy calculated for **1** at the M06/def2‐TZVPP//BP86‐D3BJ/def2‐TZVPP level of theory.

Donor Orbital	Accepter Orbital^[a]^	Energy [kcal mol^−1^]
LP^[b]^(σ) N4	Si1–N σ*	13.92
LP(σ) N4	Si2–N σ*	20.81
LP(π) N4	Si1–N σ*	21.01
LP(π) N4	Si2–N σ*	23.81
LP(σ) N3	Si1–N σ*	22.9
LP(σ) N3	Si5–C σ*	14.43
LP(π) N3	Si1–N σ*	26.1
LP(π) N3	Si5–C σ*	16.46

[a] Stabilization energies shown are from summing up the contributions from the corresponding individual accepter orbitals. [b] Lone pair (LP).

The bond dissociation energies (M06/def2‐TZVPP//BP86/D3‐BJ/def2‐TZVPP) of Si−N bonds involving dicoordinated N atoms viz., N3 and N4 in compound **1** were calculated. The calculated values are in the range of 125.7–137.5 kcal mol^−1^ (Table S16). These high bond dissociation energy values indicate strong binding interaction between Si and N atoms in compounds **1**. The bond dissociation energy values suggest that Si_silyl_−N bonds are relatively weaker than the other Si−N bonds. The topology of electron density was analyzed using Bader's quantum theory of atoms in molecules (QTAIM) theory to get additional insights into the electronic structure and bonding. The Laplacian of the electron density plotted in the plane of Si1‐N4‐Si2 and Si1‐N3‐Si5 are given in Figures 3 b and 3 c. The bond critical points (BCP) of Si2−N4, Si1−N4, Si1−N3 and Si5−N3 bonds have a large positive Laplacian of electron density. This indicates the highly polar nature of the Si−N bonds, correlating with the NBO charge data. The bond ellipticity values at the BCP of Si−N bonds are 0.1020 (Si1−N3), 0.0801 (Si1−N4), and 0.07801 (Si2−N4), indicating partial multiple bond character of these bonds (Table 2). This clearly shows that one type of hyperconjugative interaction is stronger than the other. Here, the π lone pair shows larger hyperconjugation to the Si−N σ* orbitals as compared to the σ lone pair (Table 1). The contour plot of the Laplacian in the plane of Si1‐N4‐Si2 and Si1‐N3‐Si5 features valence shell charge concentration pointing from dicoordinated nitrogen, which can be considered as π‐type lone pair. Based on the structure and bonding analysis, the bonding scheme as shown in Scheme S4(a) is proposed for compound **1**. The dicoordinated nitrogen atoms in compound **1** can be considered as monovalent having two lone pairs of electrons. The hyperconjugative interactions stabilize these lone pairs. Since the strength of the hyperconjugative interaction is relatively high and closer to that of the Si−N π‐bond strength, the N3‐Si1‐N4‐Si2 skeleton can be considered as similar to silaazatriene.

**Table 2:** Topological parameters of the electron density at the bond critical points of selected bonds calculated at the M06/def2‐TZVPP//BP86‐D3BJ/def2‐TZVPP level of theory of **1**. All the quantities are in atomic units.^[a]^

	Si1–N3	Si5–N3	Si1–N4	Si2–N4
*ρ*(*r*)	0.157829	0.140358	0.148546	0.160216
∇^2^ *ρ*(*r*)	0.745988	0.598918	0.649774	0.754357
*H*(*r*)	−0.081618	−0.068818	−0.075232	−0.084398
*G*(*r*)	0.268115	0.218548	0.237676	0.272987
*V*(*r*)	−0.34973	−0.287366	−0.312909	−0.357386
*ϵ*	0.102039	0.007530	0.080627	0.078042

[a] Electron density *ρ*(*r*), Laplacian of electron density ∇^2^ *ρ*(*r*), Energy density *H*(*r*), Lagrangian kinetic energy *G*(*r*), Potential energy density *V*(*r*), ellipticity *ϵ*.

To understand the accessibility of the lone pairs, we have calculated the first and second proton affinity on both dicoordinated nitrogen center (Figure S21). The first protonation occurs at the σ‐type lone pair on N3 and N4. The proton affinity at N3 (281.0 kcal mol^−1^) is slightly higher than that at N4 (268.7 kcal mol^−1^). This agrees with the higher hyperconjugative donation from the σ‐type lone pair on N3 (37.3 kcal mol^−1^) compared to hyperconjugative donation from (34.7 kcal mol^−1^). The second proton affinity values of N3 and N4 are 172.1 kcal mol^−1^ and 161.0 kcal mol^−1^, confirming the presence of two highly active lone pairs on both of these nitrogen centers. The first and second proton affinity values are significantly higher as compared to reported values for the dicoordinated nitrogen cation compounds having two lone pairs on nitrogen.^[19]^ For instance, the first proton affinities reported by Bharatam et al. for the acyclic, dicoordinated nitrogen cation compounds having two lone pairs fall in the range of 90–130 kcal mol.^[19b]^ Hence, the dicoordinated nitrogen centers are considered similar to the carbon atom in carbones.^[20]^

We have also calculated the ligating ability of the dicoordinated nitrogen centers N3 and N4 in compound **1** at the M06/def2‐ TZVPP//BP86/D3‐BJ/def2‐TZVPP level of theory. The complexation energy calculated by the reaction (**1+**W(CO)_6_ → **1**‐W(CO)_5_ + CO) is endothermic by 23.5 kcal mol^−1^ for N3 coordination and 37.8 kcal mol^−1^ for N4 coordination (Figure S23, Table S15). These energetics indicate that the dicoordinated N‐centers are less reactive as compared to CO and the dicoordinated N3 center is more reactive than N4. The complexation energies are corroborated well with proton affinity values, and the energies correspond to hyperconjugative donation of a lone pair of electrons to Si−N σ*‐orbitals.

The compound **2** is obtained by the reaction of bis(silylene) with Me_3_SiN_3,_ and the reaction (Scheme S2) is found to be highly exothermic (196.2 kcal mol^−1^) and exergonic (−188.4 kcal mol^−1^). The four‐membered SiNSiN ring is planar (Figure S17‐b). There are two sets of Si−N bonds in the ring. The Si1−N3 and Si1−N4 bonds are shorter (1.674 and 1.670 Å) than Si2−N3 and Si2−N4 bonds (1.833 and 1.823 Å). The molecular orbital analysis (Figure S19) shows that HOMO and HOMO−1 represent the two perpendicular lone pairs on N5. HOMO−2 represents the σ‐type lone pair on N6. HOMO−4 and HOMO−8 represent the linear combinations of the π‐type lone‐pair orbitals on the ring nitrogen atoms. The lone pairs on the dicoordinated nitrogen N5 are involved in the hyperconjugative interaction with Si2−N σ* orbitals, making two of the Si−N bonds (Si2−N3 and Si2−N4) longer than the others (Table S8). This is reflected in the lone pair occupancies, which are 1.80 e and 1.74 e. Also, the total hyperconjugative stabilization energies for the donation of these lone pairs to Si2−N3 and Si2−N4 σ* orbitals are 21.38 kcal mol^−1^ and 15.55 kcal mol^−1^, respectively. The calculated bond dissociation energies (M06/def2‐TZVPP//BP86/D3‐BJ/def2‐TZVPP) of Si−N bonds involving the dicoordinated N atom (N5) in compound **2** are in the range of 127.1–132.8 kcal mol^−1^ (Table S16). These high bond dissociation energy values indicate a strong binding interaction between Si and N atoms in compound **2**. The bond dissociation energy values suggest that the Si_silyl_−N bond is relatively weaker than the other Si−N bond.

QTAIM analysis of **2** shows a ring critical point (RCP) at the center of Si‐N‐Si‐N with comparatively small values of electron density and Laplacian of electron density. The considerably large magnitude, as well as a positive sign for the Laplacian values for all the four Si−N bonds of the ring, is indicative of the highly polar nature of Si−N bonds. The location of the corresponding BCPs is closer to the silicon center, which is another feature of a polar bond. The large Laplacian at Si2−N5 bond critical point (0.8551 in a.u) corroborates well with its shorter bond length and the energy values from the second‐order perturbation theory analysis. The non‐zero ellipticity values for Si1−N3 and Si1−N4 (0.1959 and 0.1316) indicates a partial double‐bond nature of these bonds. The mechanism of **1** is given in the Supporting Information (see Scheme S5).

In summary, the disilylene LSi‐SiL exhibits unique reactivity in the presence of Me_3_SiN_3_ to form the silaazatriene **1** and silaimine **2**. Such curiosities are rarely reported for bis(silylene) reactivity with azides. A pendent N(SiMe_3_) forms a LSi→N(SiMe_3_) coordinating bond, which is stabilized by hyperconjugative interactions in **1**. Compound **2** consists of a central Si_2_N_2_ four‐membered ring in which N(SiMe_3_) and N(*t*Bu) groups bridge two silicon atoms. The bonding and reactivity studies carried out for **1** and **2** show the presence of dicoordinated, monovalent N atoms having two lone pair of electrons. The reaction of **1** with silver triflate leads to coordination of nitrene to silver (**3**). The nitrene center of **1** is comparable to that of the carbon center of carbone.

## Experimental Section

Crystallographic analysis: All crystals were selected under cooling, using a X‐Temp2 device.^[21]^ The diffraction data were collected using an Incoatec Mo Microsource^[22]^ and a Bruker Apex II detector. The data were integrated with SAINT.^[23]^ A multi‐scan absorption correction was applied using SADABS.^[24]^ The structures were solved by SHELXT^[25]^ and refined on *F* ^2^ using SHELXL^[26]^ in the graphical user interface ShelXle.^[27]^

## Conflict of interest

The authors declare no conflict of interest.

## Supporting information

As a service to our authors and readers, this journal provides supporting information supplied by the authors. Such materials are peer reviewed and may be re‐organized for online delivery, but are not copy‐edited or typeset. Technical support issues arising from supporting information (other than missing files) should be addressed to the authors.

Supporting Information

Supporting Information
