# Supplementary material for: Stabilization of Reactive Nitrene by Silylenes without Using a Reducing Metal
Source: Angew Chem Int Ed Engl. 2021 Nov 10;60(52):27206–11. doi: 10.1002/anie.202110456 (PMC9299049; doi:10.1002/anie.202110456)
Supplement: Supplementary file 2 — Supporting Information [file ANIE-60-27206-s002.pdf]

Angewandte Chemie
Eine Zeitschrift der Gesellschaft Deutscher Chemiker

Supporting Information

Stabilization of Reactive Nitrene by Silylenes without Using a Reducing Metal

Yi Ding[+], Samir Kumar Sarkar[+], Mohd Nazish[+], Shahila Muhammed[+], Daniel Lüert, Paul Niklas Ruth, Christina M. Legendre, Regine Herbst-Irmer, Pattiyil Parameswaran, Dietmar Stalke,* Zhi Yang,* and Herbert W. Roesky**

anie_202110456_sm_miscellaneous_information.pdf
anie_202110456_sm_cif.zip

Supporting Information

Table of Contents:

(S1) Experimental Section (S1)

(S2) X-Ray Crystallographic Analysis (S11)

(S3) Theoretical calculations (S19)

(S4) References (S67)

(S1) Experimental Section:

All manipulations were carried out using standard Schlenk and glove box techniques under an atmosphere of high purity dinitrogen. Hexane, toluene and THF were distilled over Na/K alloy (25:75). Deuterated NMR solvents C_6D_6 was dried by stirring for 2 days over Na/K alloy followed by distillation in vacuum and degassed. ^{1}H, ^{13}C, and ^{29}Si NMR spectra were recorded on Bruker Avance 200, Bruker Avance 300, and Bruker Avance 500 MHz NMR spectrometers and were referenced to the resonances of the solvent used. Microanalyses were performed by the Analytisches Labor für Anorganische Chemie für Universität Göttingen. Melting points were determined in sealed glass capillaries under dinitrogen and are uncorrected. LIFDI measurements were performed on a Joel AccuTOF spectrometer under inert atmosphere. Continuous-wave (CW) EPR spectra were recorded at X-band microwave frequencies (9 GHz) using a Bruker ElexSys E500 spectrometer with a Bruker SuperX CW bridge. The spectrometer S12 was equipped with the Bruker SHQ rectangular microwave cavity (Bruker 4122SHQ) and a helium flow cryostat (Oxford Instruments) for low temperature experiments.

Synthesis of **1.**

The bis-silylene LSi–SiL (L = PhC(NtBu)$_2$) (259 mg, 0.5 mmol) was placed in a 50 mL round bottom flask and dissolved in 30ml toluene, Me_3SiN_3 (0.21 mL, 1.55 mmol) was added at room temperature. The reaction mixture was stirred overnight, then the solvent was removed in vacuum and the product was extracted with 10 mL of toluene. After filtration, the solvent was concentrated to 5 mL under vacuum. The light-yellow solution was stored in a freezer at -30 °C for overnight to get X-ray quality colorless block shaped crystals of **1** (yield: 257 mg, 66%). ^{1}H NMR (500 MHz, C_6D_6): δ = 7.52 (*m*, 1 H, Ar-CH), 7.40 (*m*, 2 H, Ar-CH), 7.22 (*m*, 1 H, Ar-CH), 7.00 (*m*, 6 H, Ar-CH), 1.42 (*s*, 18 H, *t*Bu), 1.38 (*s*, 18 H, *t*Bu), 0.73 (*s*, 9 H, $SiMe_3$), 0.65 (*s*, 9 H, $SiMe_3$), 0.48 (*s*, 9 H, $SiMe_3$); ^{13}C NMR (126 MHz, C_6D_6): δ = 176.13 (NCN), 170.62 (PhCN), 133.31, 131.58, 130.36, 130.18, 129.19, 129.07, 128.46, 127.22, 126.63, 126.48 (Ar-C), 53.77, 52.79 ($_t$Butyl-C), 32.15, 31.27 (CH_3), 6.61, 6.33, 4.92 ($SiMe_3$); ^{29}Si NMR (99 MHz, 298 K, C_6D_6, ppm): δ = 4.25, -0.93, -27.28, -57.20, -62.95. Exact Mass: 780.5, Found: 780.5 (M); Melting range: 212-214 °C to an orange liquid; Anal (%). calcd for $C_{39}H_{73}N_7Si_5$ (780.48): 60.02; H, 9.43; N, 12.56 Found: C, 60.81; H, 9.97; N, 12.17.

Synthesis of **2**.

The bis-silylene LSi–SiL (L = PhC(NtBu)$_2$) (259 mg, 0.5 mmol) was placed in a 50 mL round bottom flask and dissolved in 30ml toluene, Me_3SiN_3 (254 mg, 2.10 mmol) was added at room temperature. The reaction mixture was stirred overnight, then the solvent was removed in

vacuum and the product was extracted with 10 mL of toluene. After filtration, the solvent was concentrated to 5 mL under vacuum. The light-yellow solution was stored in a freezer at -30 °C for overnight to get X-ray quality colorless block shaped crystals of **2** (yield: 242 mg, 70%). ^{1}H NMR (500 MHz, C_6D_6): δ = 7.51 (d, 2H, Ar-CH), 7.34 (m, 2H, Ar-CH), 7.20 (m, 1H, Ar-CH), 6.91 (m, 2H, Ar-CH), 6.84 (m, 2H, Ar-CH), 6.70 (m, 1H, Ar-CH), 1.59 (s, 9H, *t*Bu), 1.30 (s, 9H, *t*Bu), 1.11 (s, 9H, *t*Bu), 1.10 (s, 9H, *t*Bu), 0.51 (s, 9H, $SiMe_3$), 0.36 (s, 9H, $SiMe_3$); ^{13}C NMR (126 MHz, C_6D_6): δ = 186.06 (NCN), 180.49 (PhCN),147.25, 130.94, 128.07, 127.49, 126.85, 126.77, 126.52, 125.39 (Ar-C), 59.36, 55.34, 55.04, 50.67 ($_t$Butyl-C), 33.62, 31.92, 31.49, 30.81 (CH_3), 5.93, 3.18 ($SiMe_3$); ^{29}Si NMR (99 MHz, 298 K, C_6D_6, ppm): δ = -3.67, -28.48, -50.81, -70.94. Exact Mass: 692.43, Found:693.43 (M+H); Melting range: 242-245 °C to an orange-colored liquid; Anal (%). calcd for $C_{36}H_{64}N_6Si_4$ (692.43): 62.37; H, 9.31; N, 12.12 Found: C, 62.88; H, 9.47; N, 11.86.

Synthesis of **3.**

Compound **1** (78 mg, 0.1 mmol) and AgOTf (25 mg, 0.1 mmol) were placed together in a Schlenk tube and 6 ml toluene was added at room temperature. The reaction mixture was stirred overnight. After filtration, the solvent was concentrated to 3 mL under vacuum. The light-yellow solution was stored in a freezer at -30 °C to get solid precipitate of **3** (yield: 80 mg, 78%). The ^{29}Si NMR studies clearly suggested that the AgOTf is coordinated to N3 nitrene because the ^{29}Si NMR of Si5 shifted from -27.28 ppm to -10.89 ppm. Lifdi spectrum of compound **3** in toluene shows molecular ion at m/z 872.4 (M-OTf) correspondings to the formation of nitrene silver adduct **3** (See ESI, Figure S14).

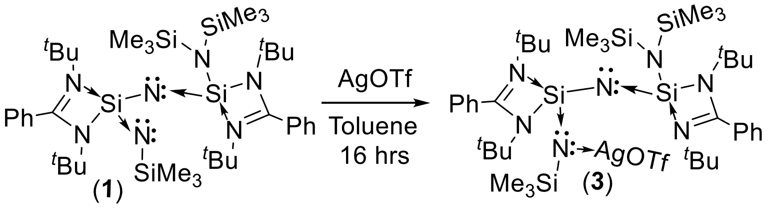

Scheme S1. Synthetic route for the preparation of **3**.

Compound **3**; ^{1}H NMR (500 MHz, C_7D_8): δ = 7.40 (*m*, 1 H, Ar-CH), 7.73-7.74 (*m*, 1 H, Ar-CH), 7.72-7.53 (*m*, 1 H, Ar-CH), 7.40-7.42 (*m*, 1 H, Ar-CH), 7.35-7.37 (*m*, 1 H, Ar-CH), 7.18-7.19 (*m*, 2 H, Ar-CH), 7.11-7.13 (*m*, 1 H, Ar-CH), 7.03-7.04 (*m*, 3 H, Ar-CH), 1.26 (*s*, 18 H, *t*Bu), 1.28 (*s*, 18 H, *t*Bu), 0.64 (*s*, 9 H, $SiMe_3$), 0.57 (*s*, 9 H, $SiMe_3$), 0.40 (*s*, 9 H, $SiMe_3$); ^{13}C NMR (126 MHz, C_7D_8): δ = 176.52 (NCN), 170.97 (PhCN), 133.71, 132.03, 130.74, 130.55, 129.57, 129.42, 128.23, 128.05, 127.86, 127.58, 126.97, 126.82 (Ar-C), 54.15, 53.16 ($_t$Butyl-C), 32.50, 31.62 (CH_3), 6.93, 6.66, 5.26 ($SiMe_3$); ^{29}Si NMR (99 MHz, 298 K, C_7D_8, ppm): δ = 6.19, 0.43,

-10.89, -63.59, -65.05; ^{19}F NMR (125 MHz, 298 K, C_7D_8, ppm): δ = 76.93; Exact Mass: 1035.33, Found: 872.4 (M-$SO_3CF_3CH_3$); Anal (%). calcd for $C_{40}H_{73}AgF_3N_7O_3SSi_5$ (1035.33): 46.31; H, 7.09; N, 9.45 Found: C, 47.22; H, 7.91; N, 9.42.

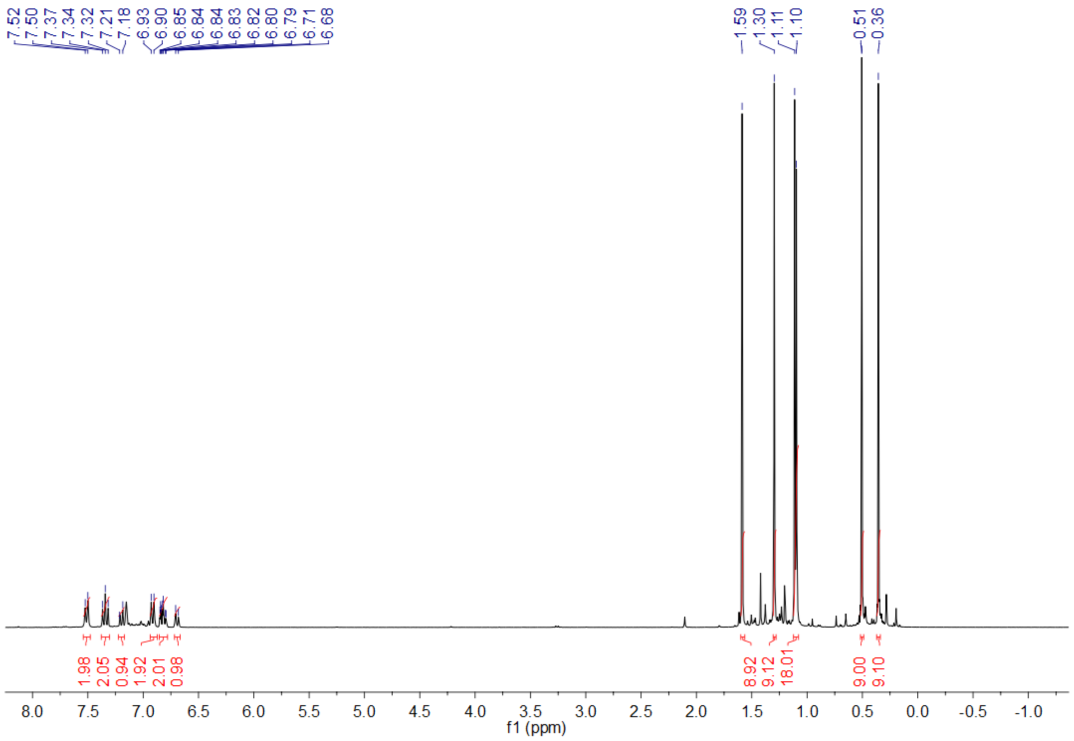

Figure S1. ^{1}H-NMR spectrum of **1**.

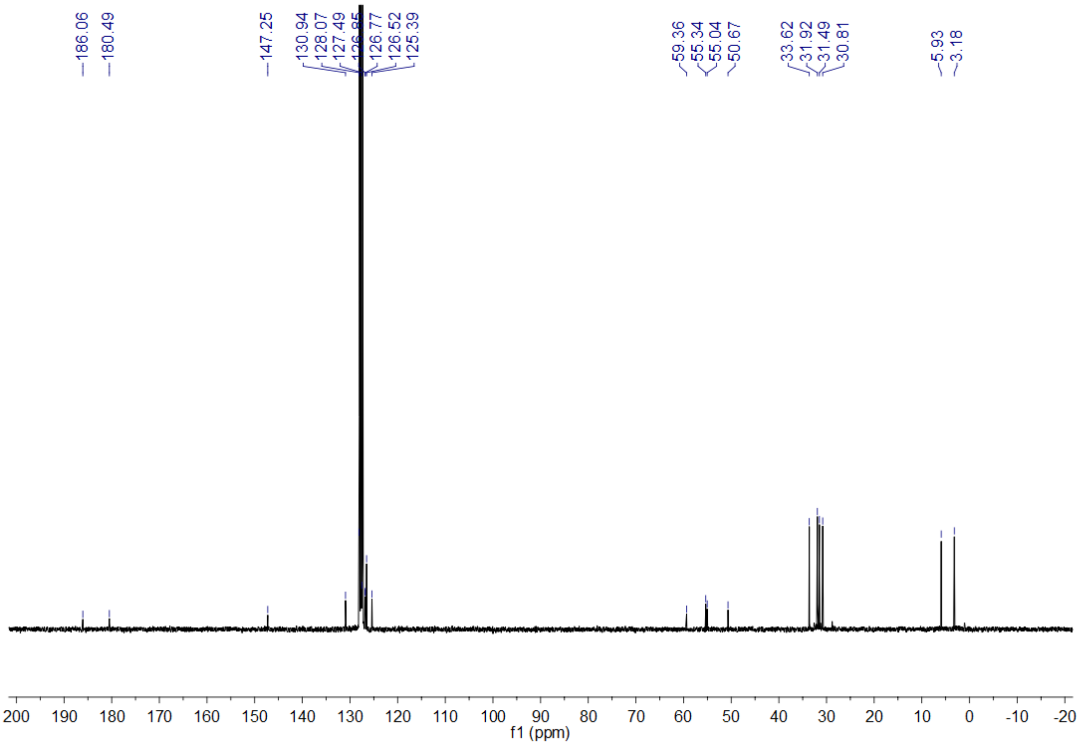

Figure S2. ^{13}C-NMR spectrum of **1**.

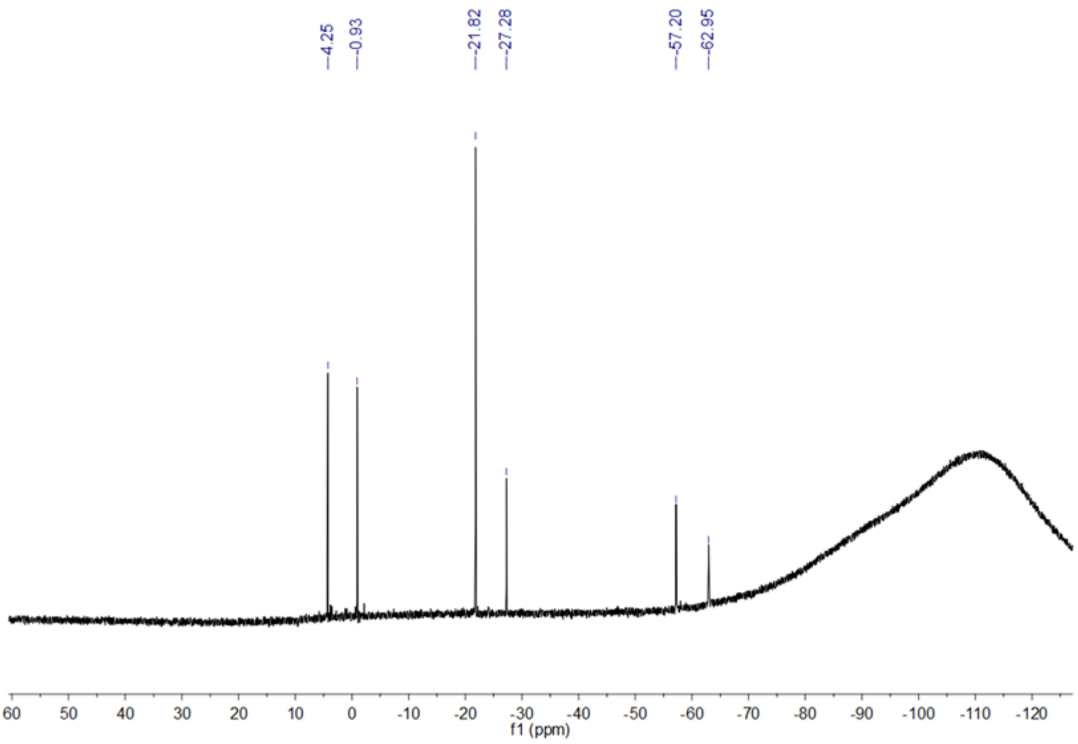

Figure S3. ^{29}Si-NMR spectrum of **1** (grease peaks at -21.82 ppm).

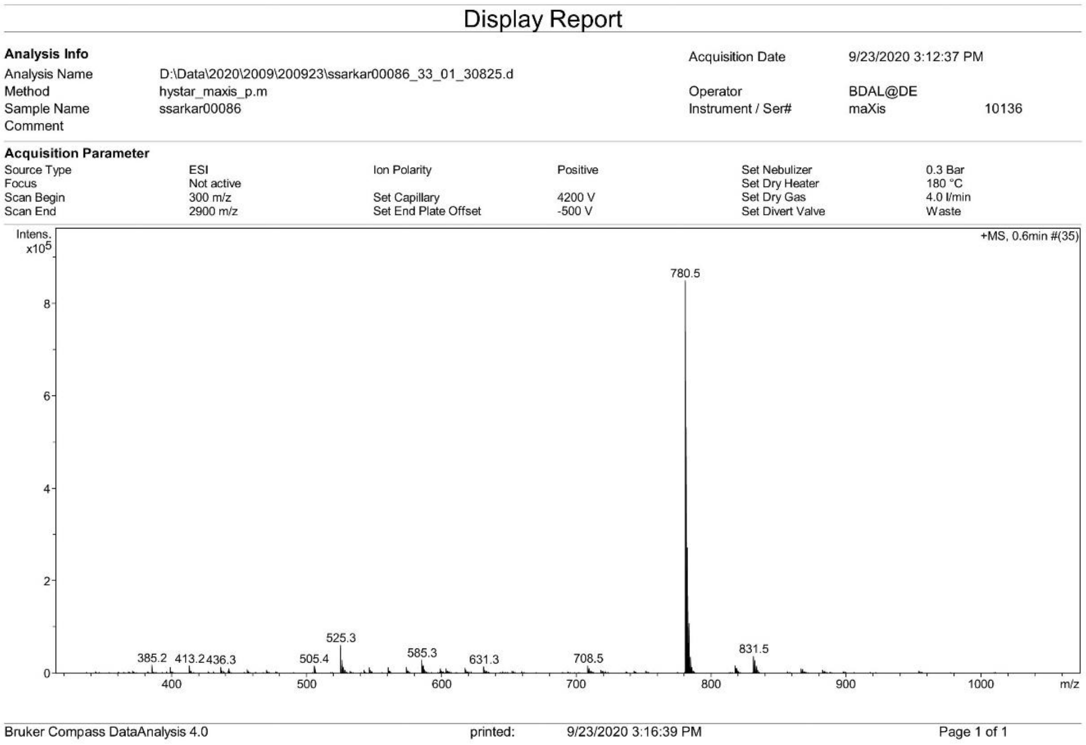

Figure S4. Mass spectrum of **1**.

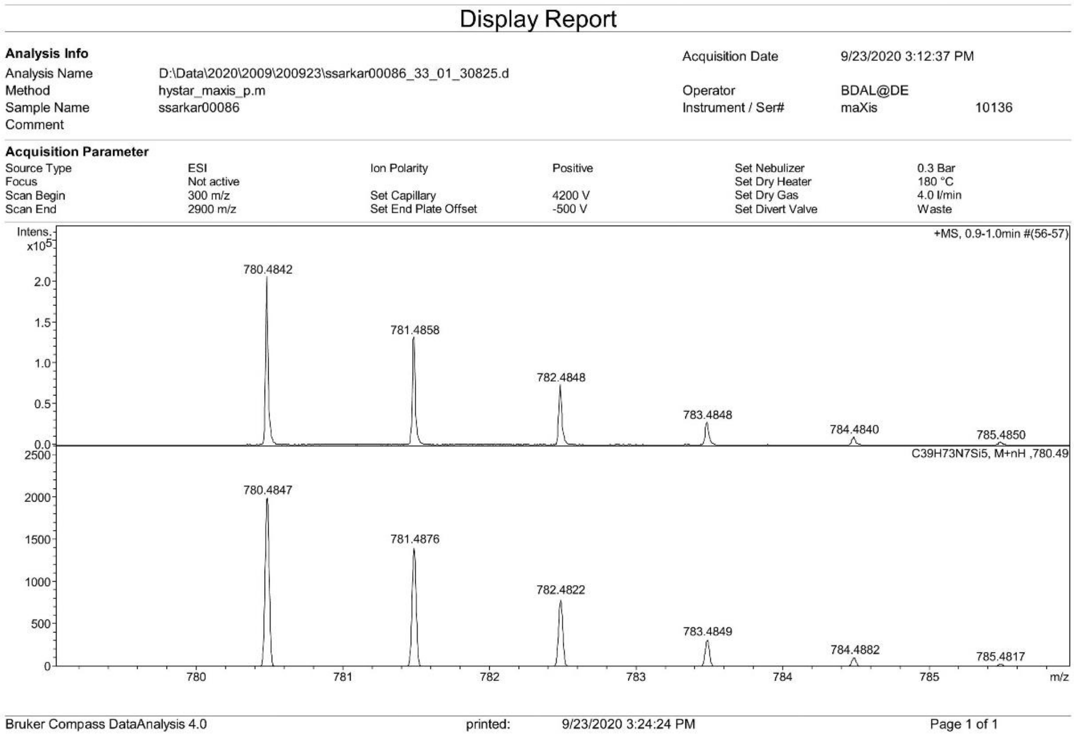

Figure S5. Isotopic mass distribution spectrum of **1**.

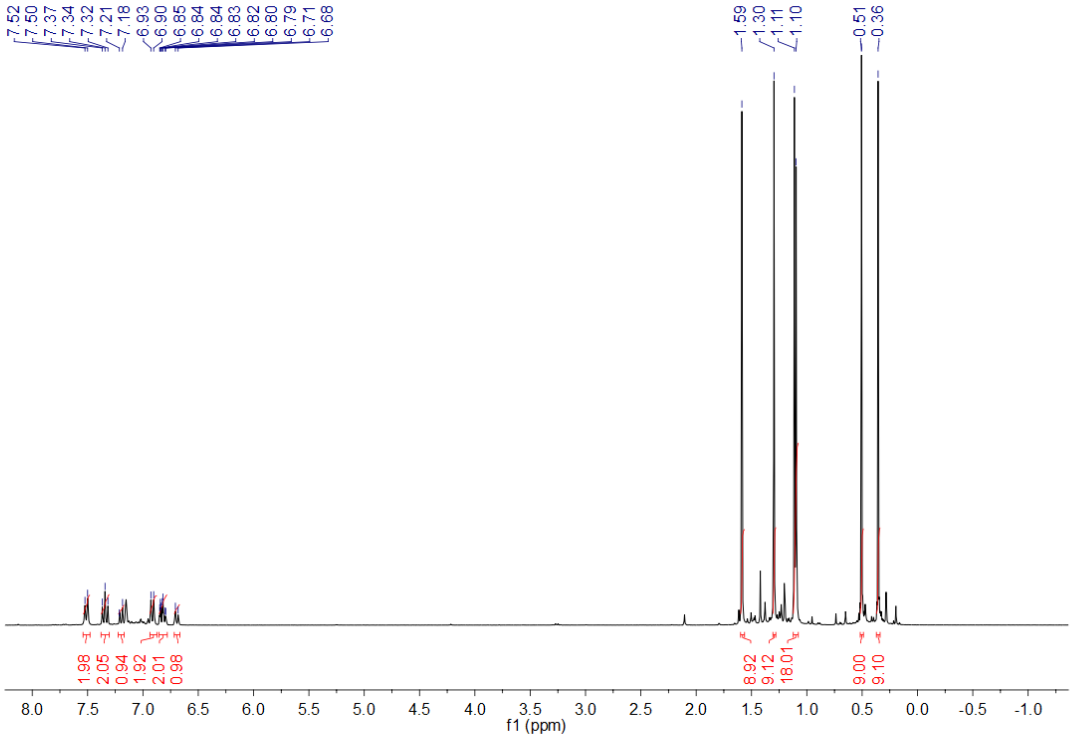

Figure S6. ^{1}H-NMR spectrum of **2**.

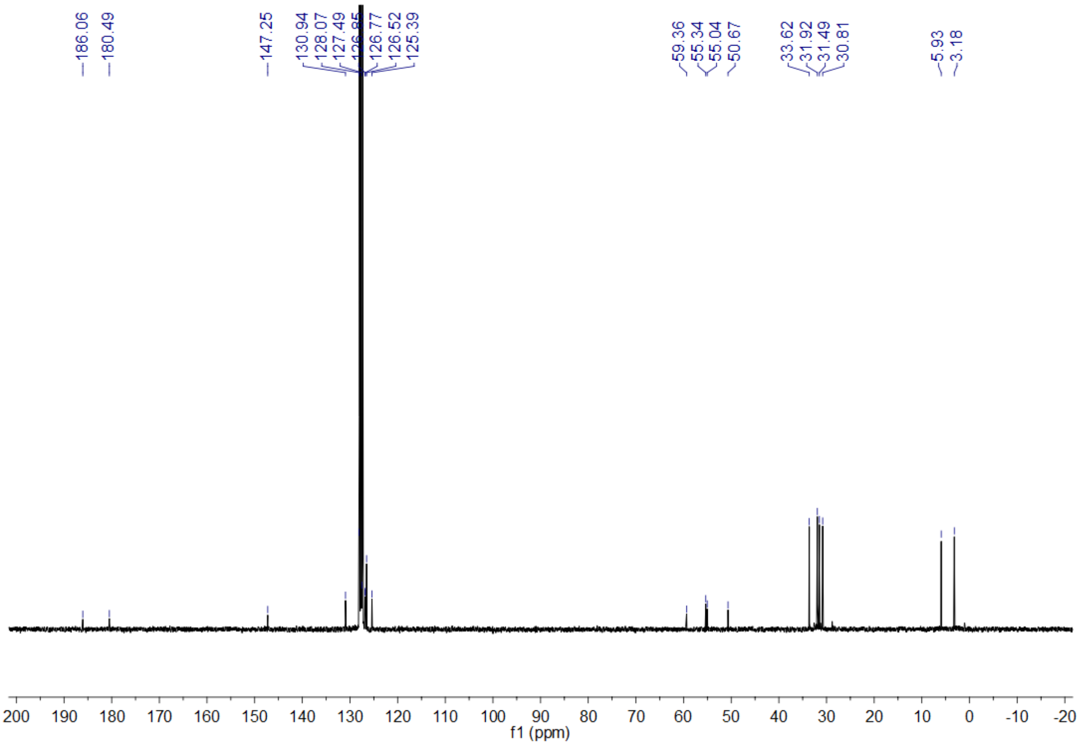

Figure S7. ^{13}C-NMR spectrum of **2**.

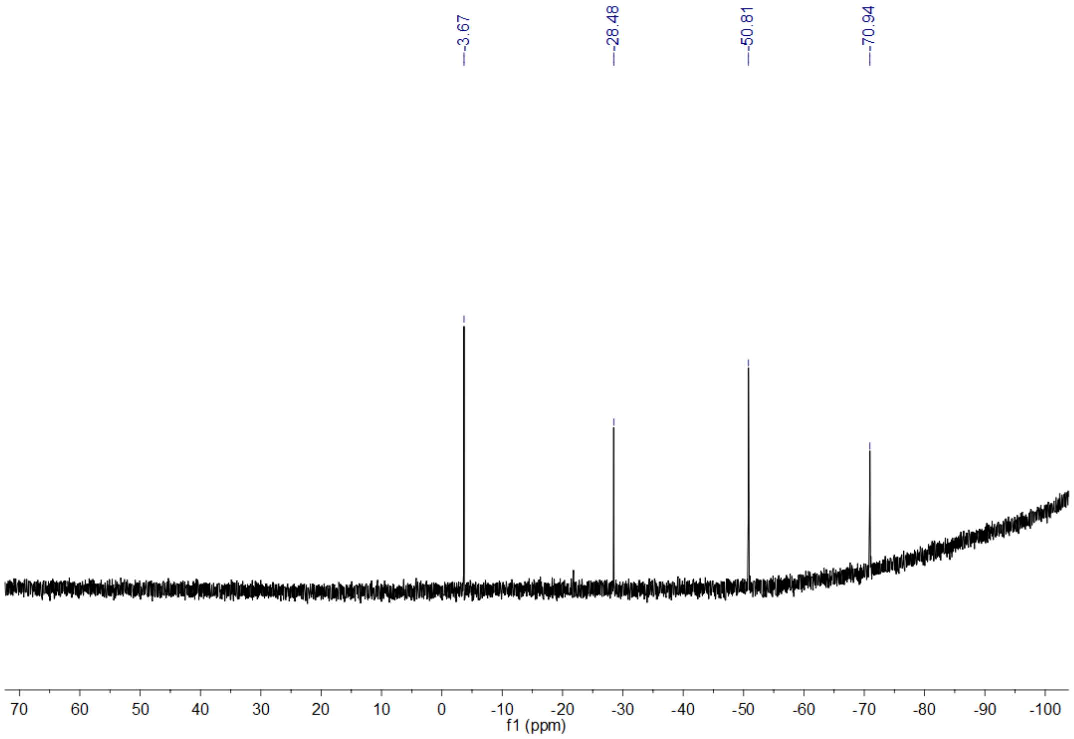

Figure S8. ^{29}Si-NMR spectrum of **2**.

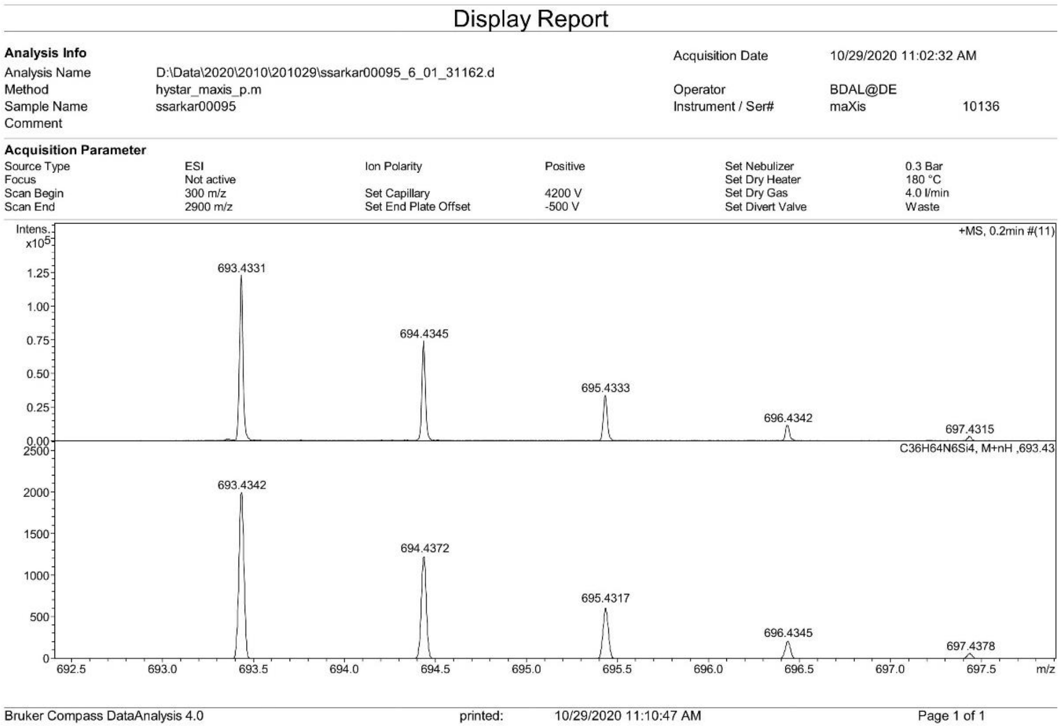

Figure S9. Isotopic mass distribution spectrum of **2**.

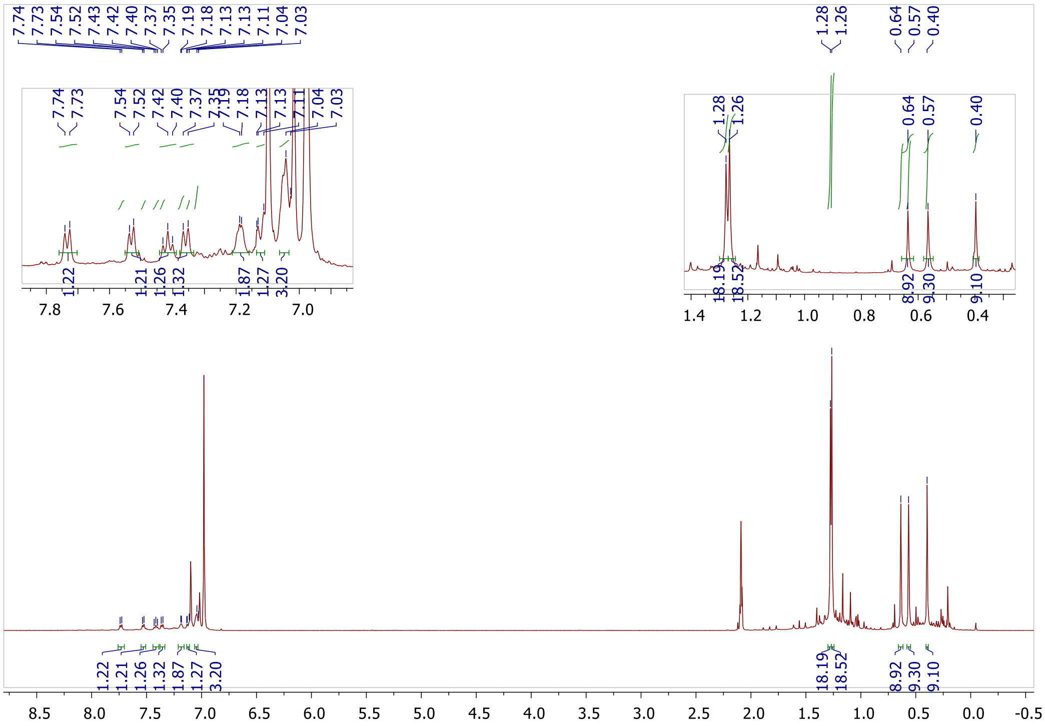

Figure S10. ^{1}H-NMR spectrum of **3** (toluene at 2.1 ppm).

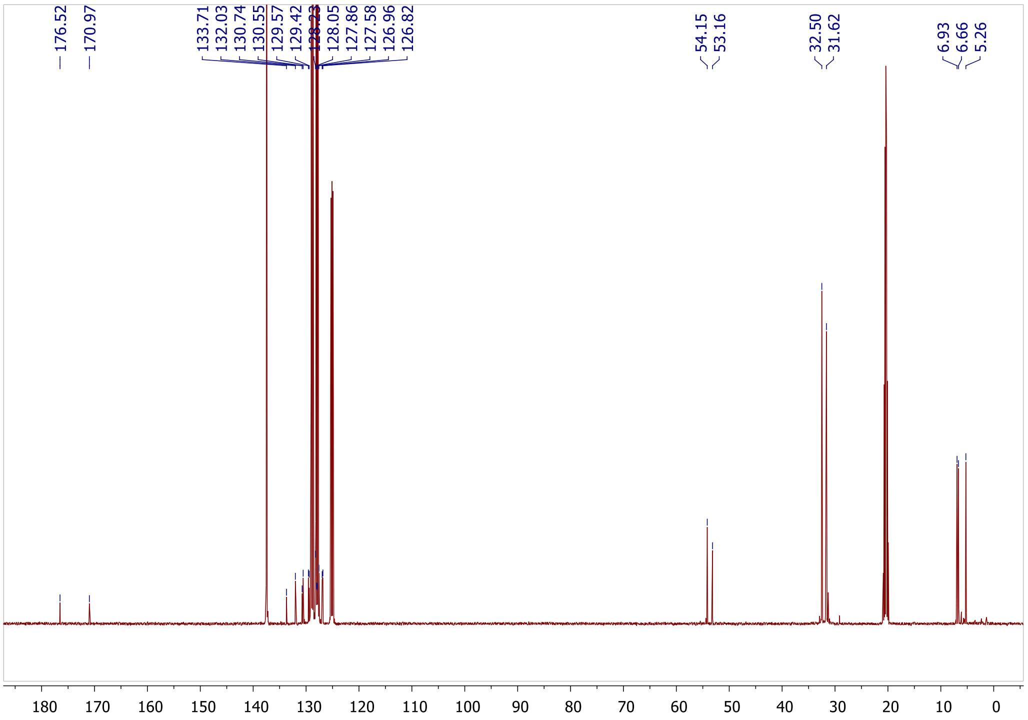

Figure S11. ^{13}C-NMR spectrum of **3**.

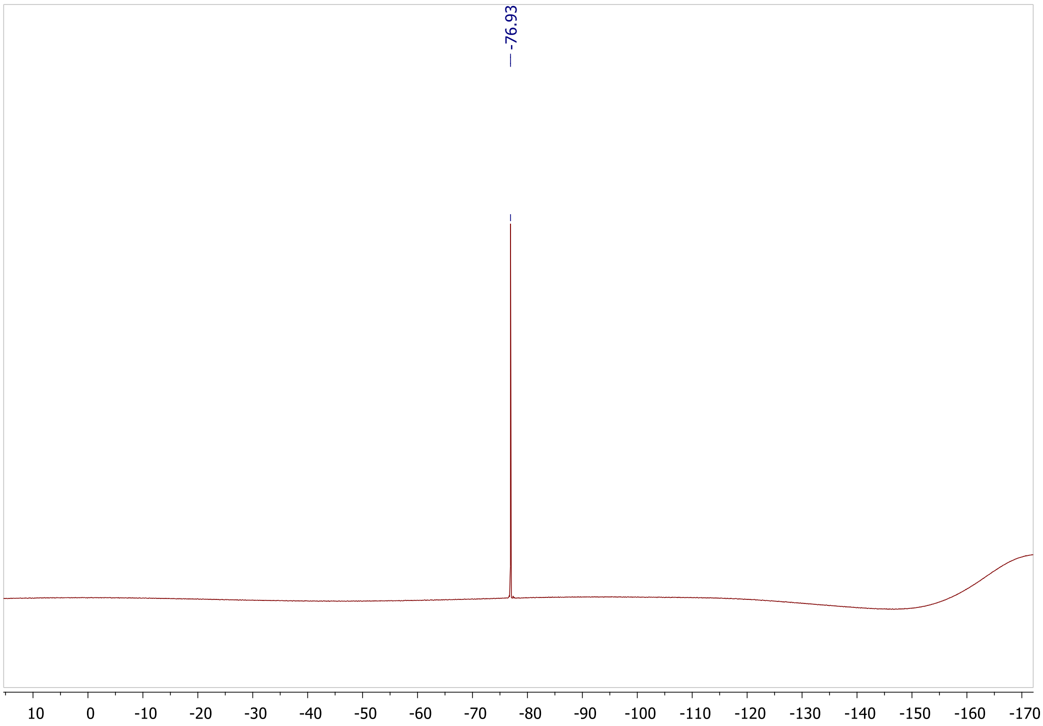

Figure S12. ^{19}F-NMR spectrum of **3**.

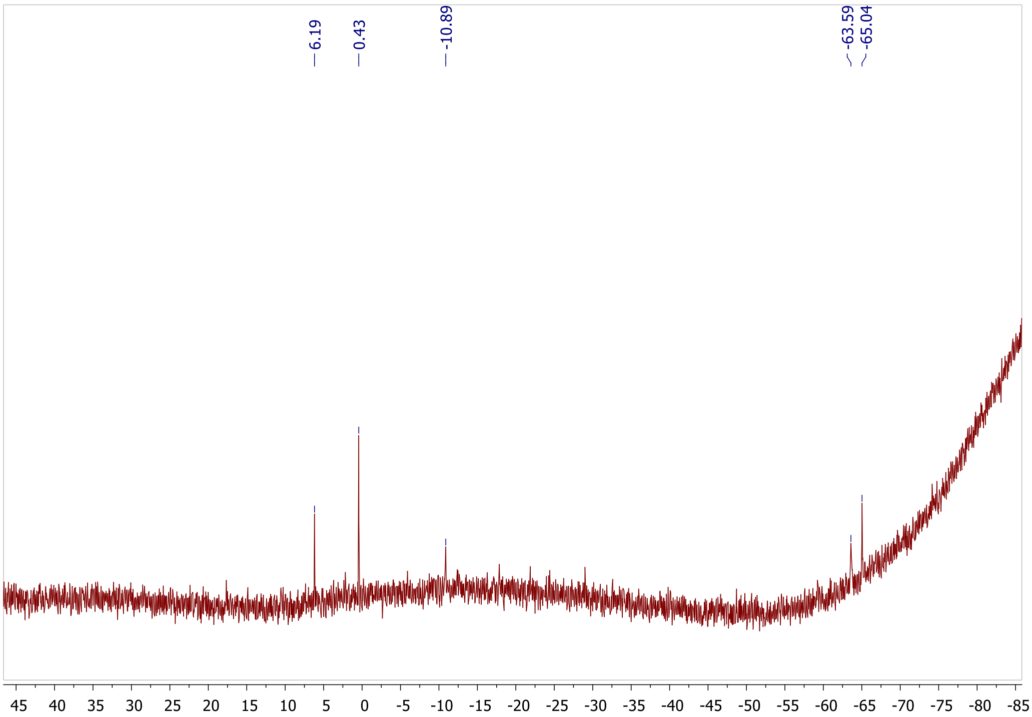

Figure S13. ^{29}Si-NMR spectrum of **3**.

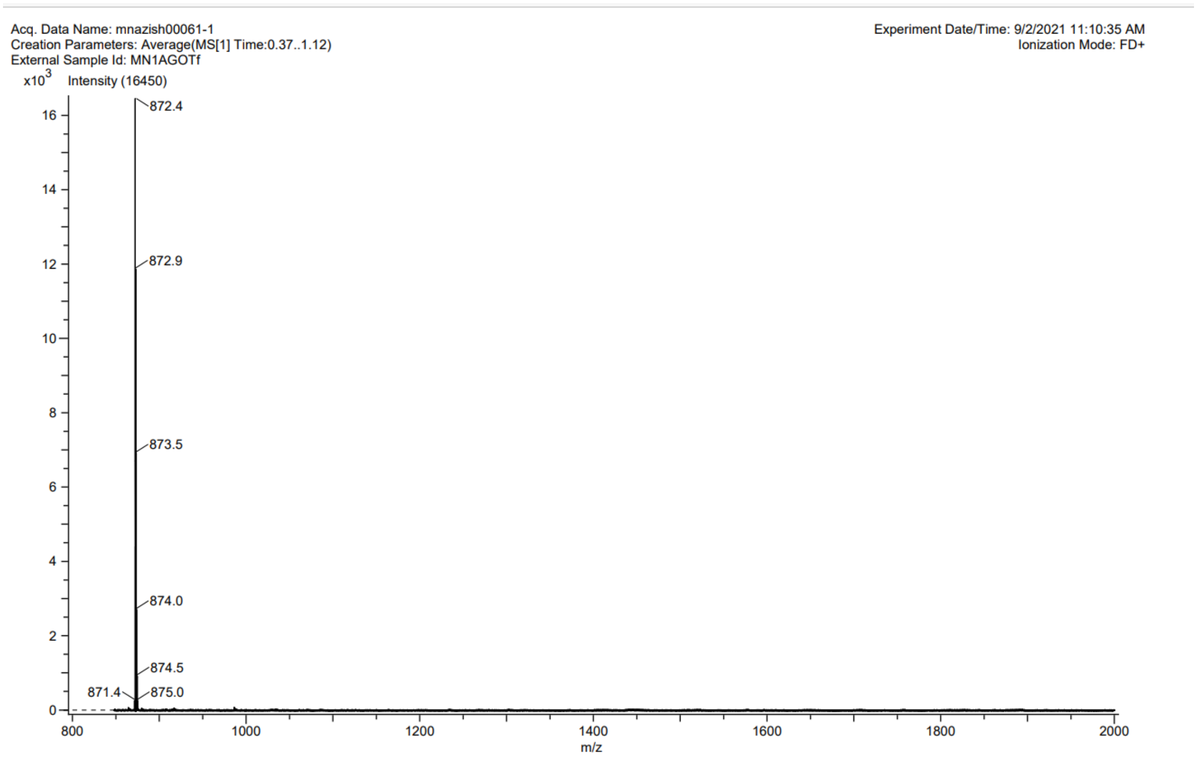

Figure 14. Mass spectrum of **3**.

(S2) X-Ray Crystallographic Analysis: All crystals were selected under cooling, using a X-Temp2 device.[1] The diffraction data were collected using an Incoatec Mo Microsource[2] and a Bruker Apex II detector. The data were integrated with SAINT.[3] A multi-scan absorption correction was applied using SADABS.[4] The structures were solved by SHELXT[5] and refined on F^2 using SHELXL[6] in the graphical user interface ShelXle.[7] an overview of the crystallographic data can be found in Table S1, while individual bond lengths and angles are listed in Table S2 for **1** and S3 for **2,** respectively.

Table S1. Crystal data and structure refinement for compounds **1-2**.

Compound	**1**	**2**
CCDC	2067321	2067320
Empirical formula	$C_{42.50}H_{77}N_7Si_5$	$C_{36}H_{64}N_6Si_4$
Formula weight	826.56	693.29
Temperature (K)	100(2)	100(2)
Crystal system	triclinic	monoclinic
Space group	$P\bar{1}$	*Cc*
a (Å)	9.957(2)	11.985(2)
b (Å)	12.538(2)	22.595(3)

c (Å)	21.328(3)	15.218(2)
α (deg)	93.84(2)	90
β (deg)	100.39(2)	94.22(2)
γ (deg)	105.63(3)	90
V (Å^3)	2503.3(8)	4109.9(10)
Z	2	4
μ/mm^{-1}	0.178	0.176
Crystal size(mm)	0.423 · 0.342 · 0.252	0.421 · 0.401 · 0.222
Θ max (deg)	26.402	29.155
Reflections collected	61735	85318
Independent reflections (R_{int})	10267 (0.0611)	11096 (0.0345)
Data/restraints/parameters	10267 / 122 / 545	11096 / 1644 / 652
Absolute structure parameter	-	0.16(10)
$R1^a$ ($I > 2\sigma(I)$)	0.0476	0.0340
$wR2^b$(all data)	0.1132	0.0927
$\Delta\rho_{max}/\Delta\rho_{min}$(e Å^{-3})	0.523/-0.412	0.370/-0.196

$^aR1 = \Sigma||F_0| - |F_c||/\Sigma|F_0|$. $^bwR_2 = [\Sigma w(F_0^2 - F_c^2)^2/\Sigma(F_0^2)]^{1/2}$

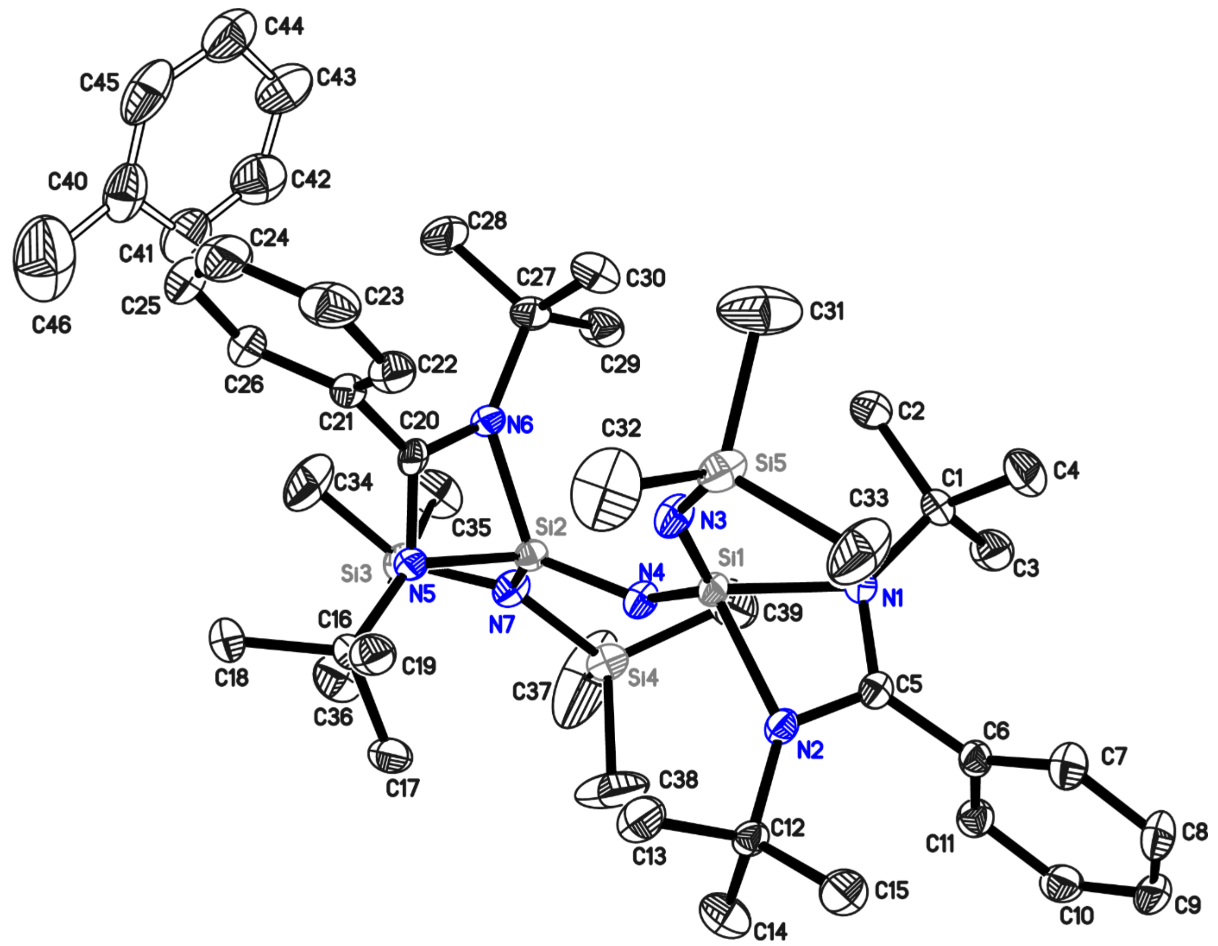

Figure S15: Asymmetric unit of **1** with thermal ellipsoids at 50% probability level. The hydrogen atoms are omitted for clarity.

The toluene molecule is disordered about an inversion center. It was refined with distance restraints and restraints for the anisotropic displacement parameters.

Table S2. Bond lengths [Å] and angles [°] for **1**.

		Si(2)-N(5)	1.8294(18)
Si(1)-N(3)	1.6126(18)	Si(2)-N(6)	1.8323(18)
Si(1)-N(4)	1.6522(18)	Si(2)-C(20)	2.275(2)
Si(1)-N(2)	1.8774(18)	N(2)-C(5)	1.332(3)
Si(1)-N(1)	1.8841(18)	N(2)-C(12)	1.480(3)
Si(1)-C(5)	2.333(2)	C(5)-C(6)	1.492(3)
C(1)-N(1)	1.479(3)	N(5)-C(20)	1.340(3)
C(1)-C(4)	1.529(3)	N(5)-C(16)	1.480(3)
C(1)-C(3)	1.530(3)	N(6)-C(20)	1.336(3)
C(1)-C(2)	1.531(3)	N(6)-C(27)	1.488(3)
N(1)-C(5)	1.330(3)	C(6)-C(11)	1.393(3)
Si(2)-N(4)	1.6115(19)	C(6)-C(7)	1.394(3)
Si(2)-N(7)	1.7212(18)	C(7)-C(8)	1.389(3)

C(9)-C(8)	1.373(4)	N(3)-Si(1)-N(4)	123.10(9)
C(9)-C(10)	1.386(4)	N(3)-Si(1)-N(2)	116.36(9)
C(10)-C(11)	1.385(3)	N(4)-Si(1)-N(2)	109.28(9)
C(12)-C(14)	1.520(3)	N(3)-Si(1)-N(1)	118.44(9)
C(12)-C(13)	1.528(3)	N(4)-Si(1)-N(1)	107.91(9)
C(12)-C(15)	1.529(3)	N(2)-Si(1)-N(1)	69.52(8)
C(16)-C(19)	1.530(3)	N(3)-Si(1)-C(5)	124.97(8)
C(16)-C(17)	1.530(3)	N(4)-Si(1)-C(5)	111.93(8)
C(16)-C(18)	1.531(3)	N(2)-Si(1)-C(5)	34.79(7)
C(20)-C(21)	1.487(3)	N(1)-Si(1)-C(5)	34.75(7)
C(21)-C(26)	1.391(3)	N(1)-C(1)-C(4)	110.33(18)
C(21)-C(22)	1.391(3)	N(1)-C(1)-C(3)	112.55(17)
C(22)-C(23)	1.387(3)	C(4)-C(1)-C(3)	110.05(19)
C(23)-C(24)	1.383(4)	N(1)-C(1)-C(2)	105.87(17)
C(24)-C(25)	1.377(4)	C(4)-C(1)-C(2)	108.89(19)
C(25)-C(26)	1.383(3)	C(3)-C(1)-C(2)	109.00(19)
C(27)-C(29)	1.524(3)	C(5)-N(1)-C(1)	129.91(17)
C(27)-C(28)	1.529(3)	C(5)-N(1)-Si(1)	91.42(13)
C(27)-C(30)	1.530(3)	C(1)-N(1)-Si(1)	138.55(14)
N(7)-Si(3)	1.7530(19)	N(4)-Si(2)-N(7)	113.96(9)
N(7)-Si(4)	1.7764(19)	N(4)-Si(2)-N(5)	117.62(9)
Si(4)-C(38)	1.830(3)	N(7)-Si(2)-N(5)	113.98(9)
Si(4)-C(39)	1.855(3)	N(4)-Si(2)-N(6)	119.56(9)
Si(4)-C(37)	1.866(3)	N(7)-Si(2)-N(6)	113.71(9)
Si(3)-C(34)	1.861(3)	N(5)-Si(2)-N(6)	71.56(8)
Si(3)-C(36)	1.871(3)	N(4)-Si(2)-C(20)	121.75(8)
Si(3)-C(35)	1.875(3)	N(7)-Si(2)-C(20)	124.28(8)
N(3)-Si(5)	1.6665(19)	N(5)-Si(2)-C(20)	36.08(7)
Si(5)-C(31)	1.871(3)	N(6)-Si(2)-C(20)	35.95(7)
Si(5)-C(32)	1.882(3)	C(5)-N(2)-C(12)	131.04(17)
Si(5)-C(33)	1.885(3)	C(5)-N(2)-Si(1)	91.67(13)
C(40)-C(45)	1.395(9)	C(12)-N(2)-Si(1)	137.29(13)
C(40)-C(41)	1.401(10)	N(1)-C(5)-N(2)	107.33(17)
C(40)-C(46)	1.419(14)	N(1)-C(5)-C(6)	127.27(18)
C(41)-C(42)	1.344(9)	N(2)-C(5)-C(6)	125.30(18)
C(42)-C(43)	1.293(8)	N(1)-C(5)-Si(1)	53.83(10)
C(43)-C(44)	1.420(10)	N(2)-C(5)-Si(1)	53.54(10)
C(44)-C(45)	1.450(10)	C(6)-C(5)-Si(1)	175.22(15)
		C(20)-N(5)-C(16)	130.78(17)

C(20)-N(5)-Si(2)	90.40(12)	C(24)-C(25)-C(26)	120.6(3)
C(16)-N(5)-Si(2)	136.09(14)	C(25)-C(26)-C(21)	119.3(2)
Si(2)-N(4)-Si(1)	136.86(11)	N(6)-C(27)-C(29)	104.73(17)
C(20)-N(6)-C(27)	131.07(17)	N(6)-C(27)-C(28)	111.95(18)
C(20)-N(6)-Si(2)	90.42(12)	C(29)-C(27)-C(28)	109.21(19)
C(27)-N(6)-Si(2)	136.39(14)	N(6)-C(27)-C(30)	110.41(17)
C(11)-C(6)-C(7)	119.4(2)	C(29)-C(27)-C(30)	110.06(19)
C(11)-C(6)-C(5)	118.51(19)	C(28)-C(27)-C(30)	110.3(2)
C(7)-C(6)-C(5)	122.06(19)	Si(2)-N(7)-Si(3)	125.30(11)
C(8)-C(7)-C(6)	119.6(2)	Si(2)-N(7)-Si(4)	114.37(10)
C(8)-C(9)-C(10)	119.9(2)	Si(3)-N(7)-Si(4)	120.08(10)
C(9)-C(8)-C(7)	120.8(2)	N(7)-Si(4)-C(38)	111.17(11)
C(11)-C(10)-C(9)	120.1(2)	N(7)-Si(4)-C(39)	113.29(11)
N(2)-C(12)-C(14)	110.53(17)	C(38)-Si(4)-C(39)	110.09(18)
N(2)-C(12)-C(13)	105.41(16)	N(7)-Si(4)-C(37)	112.12(13)
C(14)-C(12)-C(13)	109.12(19)	C(38)-Si(4)-C(37)	105.6(2)
N(2)-C(12)-C(15)	112.59(17)	C(39)-Si(4)-C(37)	104.09(19)
C(14)-C(12)-C(15)	110.29(18)	N(7)-Si(3)-C(34)	113.92(10)
C(13)-C(12)-C(15)	108.72(18)	N(7)-Si(3)-C(36)	111.22(11)
C(10)-C(11)-C(6)	120.2(2)	C(34)-Si(3)-C(36)	105.55(14)
N(5)-C(16)-C(19)	111.13(17)	N(7)-Si(3)-C(35)	111.30(11)
N(5)-C(16)-C(17)	105.00(16)	C(34)-Si(3)-C(35)	104.39(14)
C(19)-C(16)-C(17)	110.29(18)	C(36)-Si(3)-C(35)	110.12(12)
N(5)-C(16)-C(18)	110.61(18)	Si(1)-N(3)-Si(5)	140.54(12)
C(19)-C(16)-C(18)	110.59(19)	N(3)-Si(5)-C(31)	111.65(11)
C(17)-C(16)-C(18)	109.05(18)	N(3)-Si(5)-C(32)	112.59(12)
N(6)-C(20)-N(5)	106.28(17)	C(31)-Si(5)-C(32)	106.17(16)
N(6)-C(20)-C(21)	126.36(18)	N(3)-Si(5)-C(33)	114.82(11)
N(5)-C(20)-C(21)	127.24(18)	C(31)-Si(5)-C(33)	106.70(15)
N(6)-C(20)-Si(2)	53.64(10)	C(32)-Si(5)-C(33)	104.24(15)
N(5)-C(20)-Si(2)	53.51(10)	C(45)-C(40)-C(41)	119.1(7)
C(21)-C(20)-Si(2)	168.92(15)	C(45)-C(40)-C(46)	127.7(8)
C(26)-C(21)-C(22)	120.4(2)	C(41)-C(40)-C(46)	113.2(8)
C(26)-C(21)-C(20)	121.9(2)	C(42)-C(41)-C(40)	115.9(8)
C(22)-C(21)-C(20)	117.75(19)	C(43)-C(42)-C(41)	130.2(8)
C(23)-C(22)-C(21)	119.5(2)	C(42)-C(43)-C(44)	117.2(8)
C(24)-C(23)-C(22)	120.1(3)	C(43)-C(44)-C(45)	116.7(7)
C(25)-C(24)-C(23)	120.2(2)	C(40)-C(45)-C(44)	120.9(7)

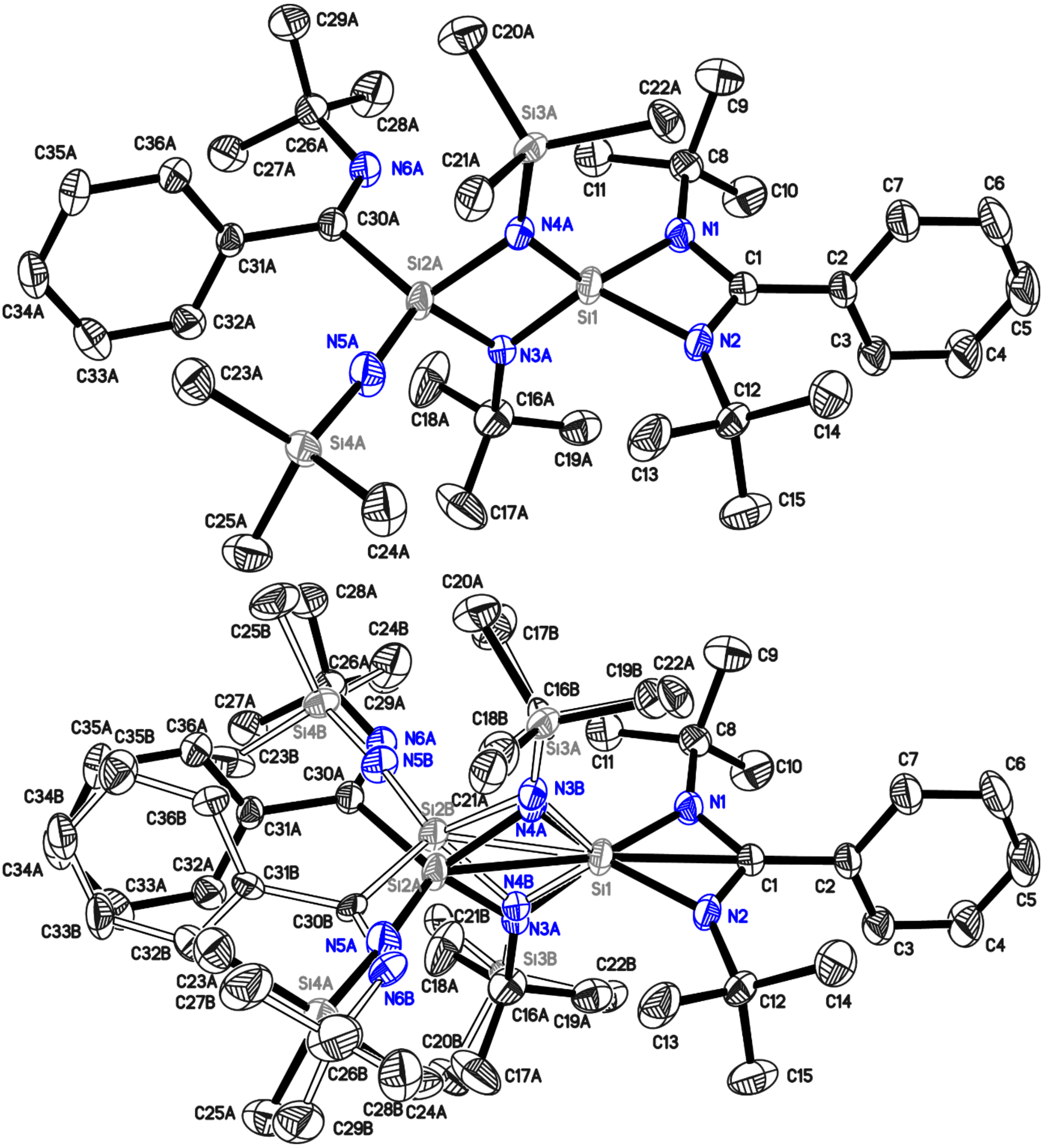

Figure S16: Top: Major disorder components for compound **2**. Bottom: Relative position of the disordered fractions. The second disorder atoms are depicted with open bonds. Hydrogen atoms are omitted for clarity. ADPs are depicted at 50% probability level.

N3-TMS is disordered with N4-^{t}Bu with a relative occupation of 51.7(5) %. Si2A to C25A belong to an independent disorder that is disordered with Si2B to C25B with a relative occupation of 76.8(3)%. Bond lengths and angles have been restrained to be similar to the corresponding values in the second disorder faction. ADPs have been restrained to be similar to nearby atoms, as well as to follow a rigid bond model. ADPs of N3A/N4B, N3B/N4A, C34A/C34B and C24B/C28A have been constrained to be identical to their respective partner. The crystal was refined as an inversion twin. The fractional contribution of the minor twin component refined to 0.16(10).

Table S3. Bond lengths [Å] and angles [°] for **2**.

C(1)-N(2)	1.340(3)	C(1)-C(2)	1.481(2)
C(1)-N(1)	1.341(3)	C(1)-Si(1)	2.2568(16)

C(2)-C(7)	1.389(4)	Si(3B)-C(21B)	1.857(8)
C(2)-C(3)	1.395(4)	Si(2A)-N(5A)	1.598(4)
C(3)-C(4)	1.389(4)	Si(2A)-C(30A)	1.914(3)
C(4)-C(5)	1.371(5)	N(5A)-Si(4A)	1.650(4)
C(5)-C(6)	1.387(5)	Si(4A)-C(24A)	1.871(6)
C(6)-C(7)	1.390(4)	Si(4A)-C(25A)	1.876(5)
C(8)-N(1)	1.499(3)	Si(4A)-C(23A)	1.887(4)
C(8)-C(9)	1.517(4)	N(6A)-C(30A)	1.287(4)
C(8)-C(11)	1.521(4)	N(6A)-C(26A)	1.488(9)
C(8)-C(10)	1.528(3)	C(26A)-C(29A)	1.511(10)
C(12)-N(2)	1.485(3)	C(26A)-C(28A)	1.528(9)
C(12)-C(14)	1.528(4)	C(26A)-C(27A)	1.556(10)
C(12)-C(13)	1.532(4)	C(30A)-C(31A)	1.503(4)
C(12)-C(15)	1.534(4)	C(31A)-C(36A)	1.393(4)
Si(1)-N(4B)	1.583(19)	C(31A)-C(32A)	1.397(4)
Si(1)-N(4A)	1.596(19)	C(32A)-C(33A)	1.383(7)
Si(1)-N(3B)	1.75(2)	C(33A)-C(34A)	1.378(8)
Si(1)-N(3A)	1.753(17)	C(34A)-C(35A)	1.395(7)
Si(1)-N(1)	1.8051(19)	C(35A)-C(36A)	1.401(6)
Si(1)-N(2)	1.8114(19)	Si(2B)-N(5B)	1.589(11)
N(3A)-C(16A)	1.536(19)	Si(2B)-C(30B)	1.923(9)
N(3A)-Si(2A)	1.902(17)	N(5B)-Si(4B)	1.659(13)
C(16A)-C(19A)	1.482(15)	Si(4B)-C(24B)	1.861(18)
C(16A)-C(18A)	1.510(14)	Si(4B)-C(25B)	1.873(14)
C(16A)-C(17A)	1.523(14)	Si(4B)-C(23B)	1.878(14)
N(4A)-Si(3A)	1.700(16)	N(6B)-C(30B)	1.266(11)
N(4A)-Si(2A)	1.752(18)	N(6B)-C(26B)	1.486(19)
Si(3A)-C(20A)	1.846(10)	C(26B)-C(29B)	1.518(19)
Si(3A)-C(21A)	1.860(8)	C(26B)-C(28B)	1.522(19)
Si(3A)-C(22A)	1.867(10)	C(26B)-C(27B)	1.535(17)
N(3B)-C(16B)	1.50(2)	C(30B)-C(31B)	1.530(11)
N(3B)-Si(2B)	1.937(19)	C(31B)-C(36B)	1.382(11)
C(16B)-C(18B)	1.519(16)	C(31B)-C(32B)	1.398(11)
C(16B)-C(17B)	1.534(16)	C(32B)-C(33B)	1.390(17)
C(16B)-C(19B)	1.559(15)	C(33B)-C(34B)	1.377(19)
N(4B)-Si(3B)	1.701(16)	C(34B)-C(35B)	1.372(18)
N(4B)-Si(2B)	1.727(18)	C(35B)-C(36B)	1.371(15)
Si(3B)-C(22B)	1.841(12)		
Si(3B)-C(20B)	1.843(9)	N(2)-C(1)-N(1)	106.50(13)

N(2)-C(1)-C(2)	127.3(2)	N(3A)-Si(1)-C(1)	130.3(6)
N(1)-C(1)-C(2)	126.2(2)	N(1)-Si(1)-C(1)	36.46(9)
N(2)-C(1)-Si(1)	53.38(9)	N(2)-Si(1)-C(1)	36.43(9)
N(1)-C(1)-Si(1)	53.11(9)	C(1)-N(1)-C(8)	132.36(19)
C(2)-C(1)-Si(1)	179.3(2)	C(1)-N(1)-Si(1)	90.42(14)
C(7)-C(2)-C(3)	120.59(16)	C(8)-N(1)-Si(1)	137.21(15)
C(7)-C(2)-C(1)	119.7(2)	C(1)-N(2)-C(12)	132.10(19)
C(3)-C(2)-C(1)	119.7(2)	C(1)-N(2)-Si(1)	90.19(13)
C(4)-C(3)-C(2)	119.4(2)	C(12)-N(2)-Si(1)	137.71(16)
C(5)-C(4)-C(3)	120.3(3)	C(16A)-N(3A)-Si(1)	138.3(13)
C(4)-C(5)-C(6)	120.4(2)	C(16A)-N(3A)-Si(2A)	135.9(13)
C(5)-C(6)-C(7)	120.4(3)	Si(1)-N(3A)-Si(2A)	85.6(6)
C(2)-C(7)-C(6)	119.0(2)	C(19A)-C(16A)-C(18A)	110.7(13)
N(1)-C(8)-C(9)	110.0(2)	C(19A)-C(16A)-C(17A)	111.9(14)
N(1)-C(8)-C(11)	105.25(19)	C(18A)-C(16A)-C(17A)	113.1(10)
C(9)-C(8)-C(11)	109.7(2)	C(19A)-C(16A)-N(3A)	109.0(13)
N(1)-C(8)-C(10)	111.9(2)	C(18A)-C(16A)-N(3A)	106.3(12)
C(9)-C(8)-C(10)	110.2(2)	C(17A)-C(16A)-N(3A)	105.5(12)
C(11)-C(8)-C(10)	109.7(2)	Si(1)-N(4A)-Si(3A)	135.6(12)
N(2)-C(12)-C(14)	111.8(2)	Si(1)-N(4A)-Si(2A)	95.8(8)
N(2)-C(12)-C(13)	105.72(19)	Si(3A)-N(4A)-Si(2A)	127.8(12)
C(14)-C(12)-C(13)	110.4(2)	N(4A)-Si(3A)-C(20A)	111.0(9)
N(2)-C(12)-C(15)	109.7(2)	N(4A)-Si(3A)-C(21A)	109.6(9)
C(14)-C(12)-C(15)	110.1(2)	C(20A)-Si(3A)-C(21A)	107.1(6)
C(13)-C(12)-C(15)	109.0(2)	N(4A)-Si(3A)-C(22A)	109.3(9)
N(4B)-Si(1)-N(3B)	92.7(5)	C(20A)-Si(3A)-C(22A)	111.3(7)
N(4A)-Si(1)-N(3A)	93.9(5)	C(21A)-Si(3A)-C(22A)	108.6(7)
N(4B)-Si(1)-N(1)	125.0(8)	C(16B)-N(3B)-Si(1)	140.0(15)
N(4A)-Si(1)-N(1)	127.2(8)	C(16B)-N(3B)-Si(2B)	134.2(15)
N(3B)-Si(1)-N(1)	122.9(8)	Si(1)-N(3B)-Si(2B)	84.5(7)
N(3A)-Si(1)-N(1)	122.6(7)	N(3B)-C(16B)-C(18B)	109.9(15)
N(4B)-Si(1)-N(2)	125.7(7)	N(3B)-C(16B)-C(17B)	108.8(15)
N(4A)-Si(1)-N(2)	123.0(8)	C(18B)-C(16B)-C(17B)	113.0(13)
N(3B)-Si(1)-N(2)	121.2(8)	N(3B)-C(16B)-C(19B)	107.0(14)
N(3A)-Si(1)-N(2)	120.1(6)	C(18B)-C(16B)-C(19B)	109.4(13)
N(1)-Si(1)-N(2)	72.90(7)	C(17B)-C(16B)-C(19B)	108.4(13)
N(4B)-Si(1)-C(1)	136.0(6)	Si(1)-N(4B)-Si(3B)	134.1(13)
N(4A)-Si(1)-C(1)	135.6(6)	Si(1)-N(4B)-Si(2B)	97.1(8)
N(3B)-Si(1)-C(1)	131.3(6)	Si(3B)-N(4B)-Si(2B)	125.5(13)

N(4B)-Si(3B)-C(22B)	109.6(10)
N(4B)-Si(3B)-C(20B)	111.0(9)
C(22B)-Si(3B)-C(20B)	109.9(9)
N(4B)-Si(3B)-C(21B)	110.0(8)
C(22B)-Si(3B)-C(21B)	108.4(8)
C(20B)-Si(3B)-C(21B)	107.9(4)
N(5A)-Si(2A)-N(4A)	121.2(8)
N(5A)-Si(2A)-N(3A)	118.1(7)
N(4A)-Si(2A)-N(3A)	84.1(4)
N(5A)-Si(2A)-C(30A)	111.5(2)
N(4A)-Si(2A)-C(30A)	110.3(8)
N(3A)-Si(2A)-C(30A)	108.4(7)
Si(2A)-N(5A)-Si(4A)	175.9(4)
N(5A)-Si(4A)-C(24A)	112.9(3)
N(5A)-Si(4A)-C(25A)	111.9(3)
C(24A)-Si(4A)-C(25A)	106.6(4)
N(5A)-Si(4A)-C(23A)	112.9(2)
C(24A)-Si(4A)-C(23A)	105.1(4)
C(25A)-Si(4A)-C(23A)	106.9(2)
C(30A)-N(6A)-C(26A)	125.8(4)
N(6A)-C(26A)-C(29A)	109.4(7)
N(6A)-C(26A)-C(28A)	105.7(9)
C(29A)-C(26A)-C(28A)	108.9(8)
N(6A)-C(26A)-C(27A)	115.5(5)
C(29A)-C(26A)-C(27A)	110.0(7)
C(28A)-C(26A)-C(27A)	107.0(9)
N(6A)-C(30A)-C(31A)	127.0(3)
N(6A)-C(30A)-Si(2A)	117.5(2)
C(31A)-C(30A)-Si(2A)	115.5(2)
C(36A)-C(31A)-C(32A)	117.9(2)
C(36A)-C(31A)-C(30A)	121.8(3)
C(32A)-C(31A)-C(30A)	120.2(3)
C(33A)-C(32A)-C(31A)	121.9(4)
C(34A)-C(33A)-C(32A)	120.2(6)
C(33A)-C(34A)-C(35A)	118.9(7)
C(34A)-C(35A)-C(36A)	120.8(6)
C(31A)-C(36A)-C(35A)	120.0(4)
N(5B)-Si(2B)-N(4B)	126.8(10)
N(5B)-Si(2B)-C(30B)	111.8(6)
N(4B)-Si(2B)-C(30B)	106.7(8)
N(5B)-Si(2B)-N(3B)	120.3(10)
N(4B)-Si(2B)-N(3B)	82.2(5)
C(30B)-Si(2B)-N(3B)	104.3(8)
Si(2B)-N(5B)-Si(4B)	174.3(13)
N(5B)-Si(4B)-C(24B)	111(2)
N(5B)-Si(4B)-C(25B)	110.9(10)
C(24B)-Si(4B)-C(25B)	106.7(18)
N(5B)-Si(4B)-C(23B)	112.8(8)
C(24B)-Si(4B)-C(23B)	108.9(18)
C(25B)-Si(4B)-C(23B)	106.7(9)
C(30B)-N(6B)-C(26B)	127.0(14)
N(6B)-C(26B)-C(29B)	108.9(16)
N(6B)-C(26B)-C(28B)	107.0(17)
C(29B)-C(26B)-C(28B)	106.7(17)
N(6B)-C(26B)-C(27B)	111.6(14)
C(29B)-C(26B)-C(27B)	110.4(16)
C(28B)-C(26B)-C(27B)	112.0(18)
N(6B)-C(30B)-C(31B)	128.6(10)
N(6B)-C(30B)-Si(2B)	118.0(9)
C(31B)-C(30B)-Si(2B)	113.4(6)
C(36B)-C(31B)-C(32B)	118.4(8)
C(36B)-C(31B)-C(30B)	118.4(8)
C(32B)-C(31B)-C(30B)	123.2(8)
C(33B)-C(32B)-C(31B)	116.9(13)
C(34B)-C(33B)-C(32B)	123(2)
C(35B)-C(34B)-C(33B)	121(2)
C(36B)-C(35B)-C(34B)	116.1(18)
C(35B)-C(36B)-C(31B)	124.7(12)

(S3) Theoretical Calculations

Computational Methodology

The DFT calculations were performed in G09 program package.[8] Geometry optimizations were carried out using the GGA functional BP86, which is composed of Becke 1988 exchange functional and Perdue 86 correlation functional.[9] D3BJ empirical dispersion was included while optimizing which adds the D3 version of Grimme dispersion along with Becke-Johnson damping.[10] The def2-TZVPP basis set which is of the triple-ζ valance type, augmented with two sets of polarization functions was used for optimization.[11] The single point and natural bond orbital (NBO)[12] calculations were performed using the meta-GGA functional M06 and def2-TZVPP basis set.[13] The wavefunctions were generated at M06/def2-TZVPP level of theory using the geometries optimized at BP86/D3-BJ/def2-TZVPP level of theory. Quantum theory of atoms in molecules (QTAIM)[14] analysis was performed in Multiwfn software package.[15] The Laplacian of electron densities were plotted in the GUI unit of Multiwfn itself. The NMR spectra of **1** and **2** were calculated at M06/PCM/def2-TZVPP//BP86/D3-BJ/def2-TZVPP level of theory with benzene as solvent using the GIAO approach. The spectra were also calculated at B3LYP/PCM/6-311+G(2d,p)//BP86/D3-BJ/def2-TZVPP level of theory in benzene for comparison.[16]

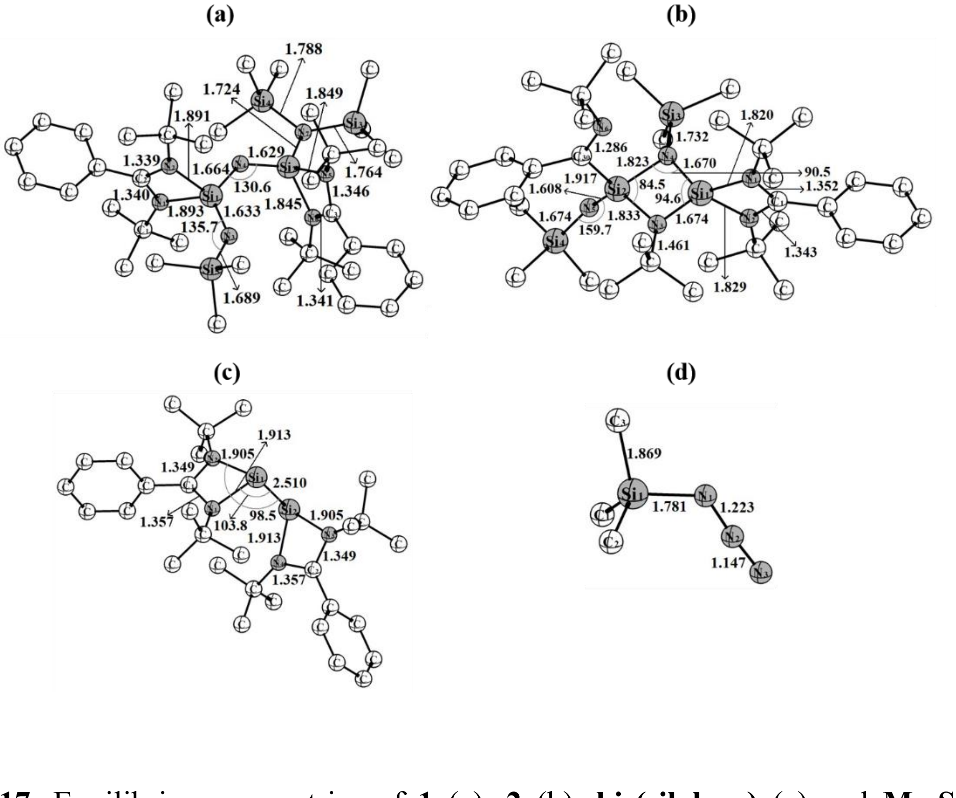

Figure S17. Equilibrium geometries of **1** (a), **2** (b), **bis(silylene)** (c) and **Me_3SiN_3** (d) at BP86/D3-BJ/def2-TZVPP level of theory. The hydrogen atoms are omitted for clarity. Distances are in Å and angles are in degrees.

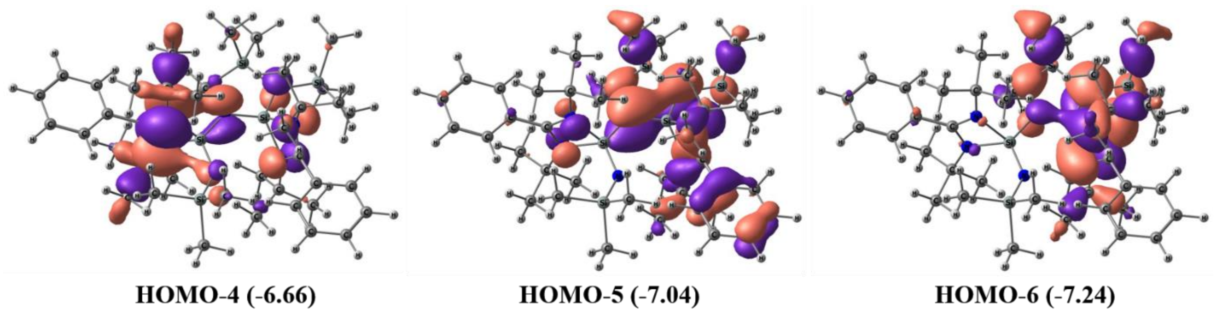

Figure S18. Plots of selected molecular orbitals of **1** at M06/def2-TZVPP//BP86/D3-BJ/def2-TZVPP level of theory. The surfaces are plotted at the iso-surface value of 0.03. The energy values given in the parentheses are in eV.

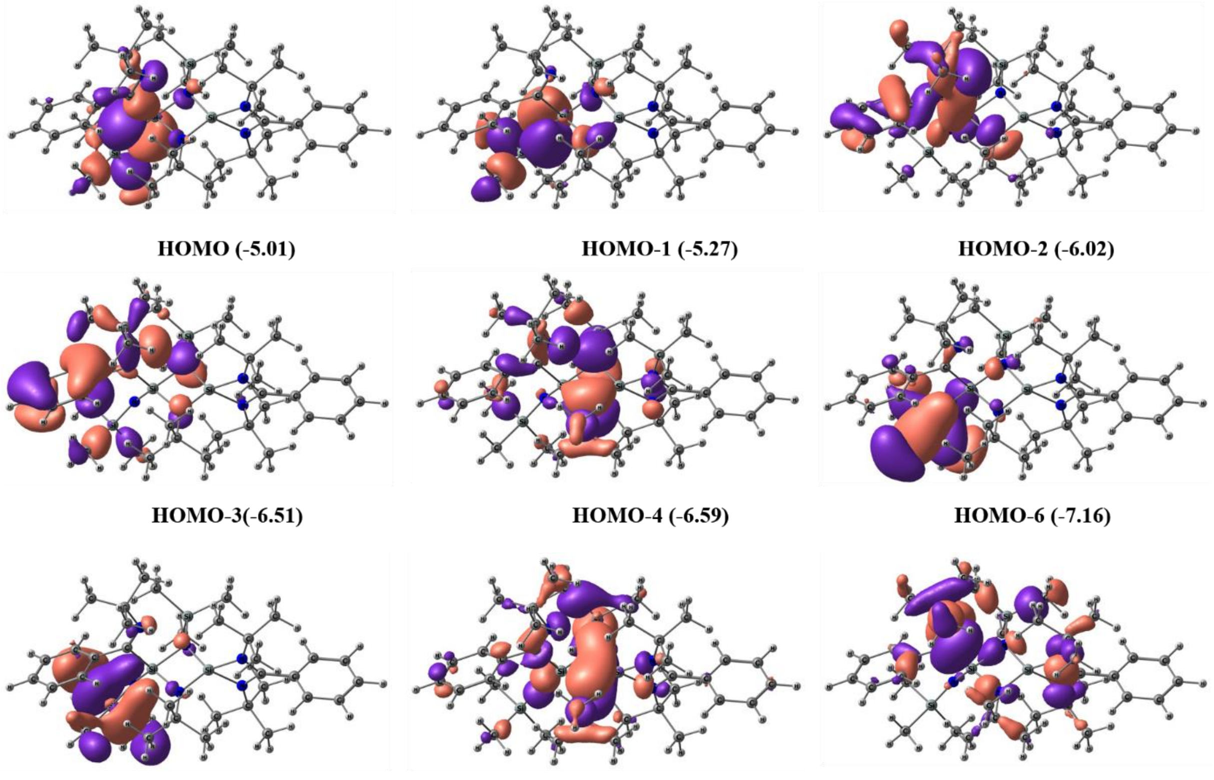

Figure S19. Plots of selected molecular orbitals of **2** at M06/def2-TZVPP//BP86/D3-BJ/def2-TZVPP level of theory. The surfaces are plotted at the iso-surface value of 0.03. The energy values given in the parentheses are in eV.

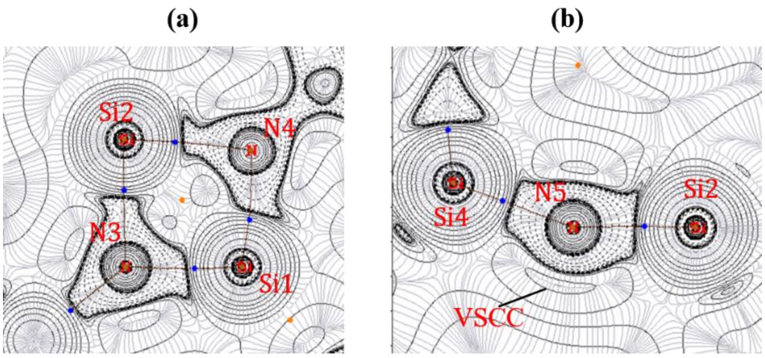

Figure S20. The Laplacian of the electron density plotted in the plane of (a) Si1-N3-Si2-N4 ring and (b) Si2-N5-Si4 of **2**. QTAIM analysis was done using the wavefunction generated at M06/def2-TZVPP//BP86/D3-BJ/def2-TZVPP level of theory. Blue dots represent the bond critical points (BCPs), orange dots correspond to the ring critical points (RCPs) and, the brown lines are the bond paths. The atom numbering is based on **Figure S12-b**.

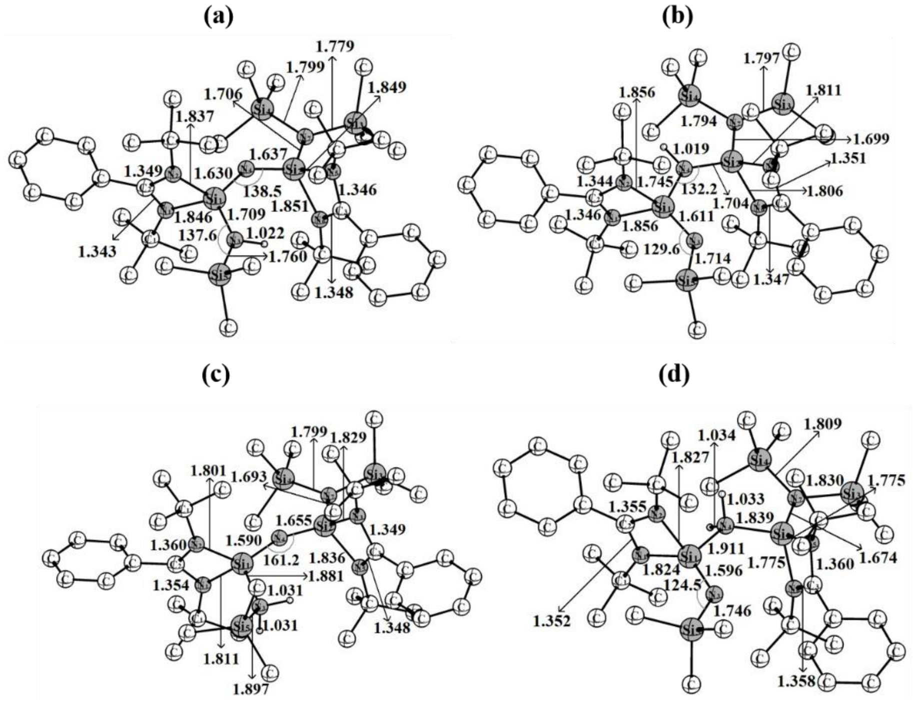

Figure S21. Equilibrium geometries of the protonated structures of **1** at BP86/D3-BJ/def2-TZVPP level of theory. (a) monoprotonation at N3, (b) monoprotonation at N4, (c) diprotonation at N3, (d) diprotonation at N4. Other hydrogen atoms are omitted for clarity. Distances are in Å and angles are in degrees.

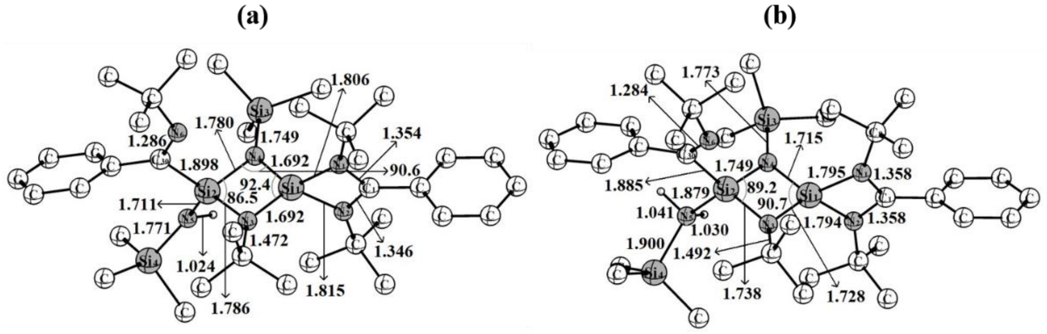

Figure S22. The equilibrium geometries of the protonated structures of **2**, optimized at BP86/D3-BJ/def2-TZVPP level of theory. (a) monoprotonation at N5, (b) diprotonation at N5. The other hydrogen atoms are omitted for clarity. Distances are in Å and angles are in degrees.

Table S4. Occupancy of lone-pairs of **1** at M06/def2-TZVPP//BP86/D3-BJ/def2-TZVPP level of theory.

LP	Occupancy	%p	%s
LP(σ) N3	1.80	97.37	2.33
LP(π) N3	1.76	99.68	
LP(σ) N4	1.81	96.28	3.27
LP(π) N4	1.75	99.55	
LP(π) N7	1.83	99.96	

Table S5. Hyperconjugative stabilization energy calculated for **1** at M06/def2-TZVPP//BP86/D3-BJ/def2-TZVPP level of theory.

Donor Orbital	Accepter Orbital	Energy (kcal/mol)
LP(σ) N3	Si1-N2 BD*	11.38
LP(σ) N3	Si1-N1 BD*	6.64
LP(σ) N3	Si1-N4 BD*	4.88
LP(σ) N3	Si5-C BD*	12.13
LP(σ) N3	Si5-C BD*	1.20
LP(σ) N3	Si5-C BD*	1.10
LP(π) N3	Si1-N2 BD*	10.77
LP(π) N3	Si1-N1 BD*	15.33
LP(π) N3	Si5-C BD*	8.68
LP(π) N3	Si5-C BD*	7.78
LP(σ) N4	Si1-N2 BD*	1.61
LP(σ) N4	Si1-N1 BD*	2.72
LP(σ) N4	Si1-N3 BD*	9.59
LP(σ) N4	Si2-N6 BD*	8.39
LP(σ) N4	Si2-N7 BD*	5.64
LP(σ) N4	Si2-N5 BD*	6.78
LP(π) N4	Si1-N2 BD*	10.19
LP(π) N4	Si1-N1 BD*	10.82
LP(π) N4	Si2-N6 BD*	11.55
LP(π) N4	Si2-N5 BD*	12.26

Table S6. Wiberg bond index of important bonds in **1** calculated at M06/def2-TZVPP//BP86/D3-BJ/def2-TZVPP level of theory.

Bond	WBI
Si1-N4	0.79
Si2-N4	0.92
Si1-N3	0.94
Si2-N7	0.61
Si5-N3	0.78
Si1-N1	0.39
Si1-N2	0.40
Si2-N5	0.42
Si2-N6	0.42

Table S7. Charges by natural population analysis (M06/def2-TZVPP//BP86/D3-BJ/def2-TZVPP) on selected atoms of **1**.

Atom	Natural Charge
Si1	2.29
Si2	2.36
Si3	1.90
Si4	1.91
Si5	1.88
N1	-0.71
N2	-0.71
N3	-1.81
N4	-1.83
N5	-0.76
N6	-0.75
N7	-1.79

Table S8. Hyperconjugative stabilization energy calculated for **2** at M06/def2-TZVPP//BP86/D3-BJ/def2-TZVPP level of theory.

**Donor orbital**	**Accepter orbital**	**Energy (kcal/mol)**
LP (π) N5	Si2-N4 BD*	9.08
LP (π) N5	Si2-N3 BD*	1.83
LP (π) N5	Si2-C30 BD*	15.03
LP (π) N5	Si4-C BD*	9.66
LP (π) N5	Si4-C BD*	0.62
LP (π) N5	Si4-C BD*	6.62
LP (σ) N5	Si2-N4 BD*	6.47
LP (σ) N5	Si2-N3 BD*	19.55
LP (σ) N5	Si2-C30 BD*	0.66
LP (σ) N5	Si4-C BD*	1.51
LP(σ) N5	Si4-C BD*	8.47
LP(σ) N5	Si4-C BD*	5.57
LP(σ) N6	Si2-C30 BD*	4.55
LP(σ) N6	Si2-N5 BD*	1.10

Table S9. Occupancy of lone-pairs of **2** at M06/def2-TZVPP//BP86/D3-BJ/def2-TZVPP level of theory.

**Lone-pair**	**Occupancy**	**%p**	**%s**
LP (π) N5	1.80	99.93	
LP (σ) N5	1.74	99.17	0.78
LP(σ) N6	1.81	94.14	5.62
LP(π) N3	1.80	99.80	
LP(π) N4	1.81	99.71	0.15

Table S10. Charges by natural population analysis (M06/def2-TZVPP//BP86/D3-BJ/def2-TZVPP) on selected atoms of **2**.

**Atom**	**Natural Charge**
Si1	2.33
Si2	2.15
Si3	1.89
Si4	1.89
N1	-0.76
N2	-0.74
N3	-1.33
N4	-1.76
N5	-1.78
N6	-0.45
C1	0.56
C30	-0.12

Table S11-a. Topological parameters of the electron density at the bond critical points of Si1-N3-Si2-N4 ring of **2** at M06/def2-TZVPP//BP86/D3-BJ/def2-TZVPP level of theory. All the quantities are in atomic units.

**CP (3,-1) for Si1-N3-Si3-N4 ring bonds**				
	Si2-N3	Si2-N4	Si1-N3	Si1-N4
Electron density ρ (r)	0.106323	0.108230	0.146242	0.147886
Laplacian of electron density $\nabla^2\rho$ (r)	0.332427	0.342899	0.625382	0.634570
Energy density H(r)	-0.050328	-0.051621	-0.074603	-0.761427
Lagrangian kinetic energy G(r)	0.133435	0.137345	0.230948	0.234785
Potential energy density V(r)	-0.183763	-0.188967	-0.305552	-0.310928
Ellipticity ε	0.039965	0.049817	0.195932	0.131661

Table S11-b. Topological parameters of the electron density at the ring critical point of Si1-N3-Si2-N4 ring of **2** at M06/def2-TZVPP//BP86/D3-BJ/def2-TZVPP level of theory. All the quantities are in atomic units.

**CP (3,+1) at Si1N3Si2N4 ring**	
Electron density<br>ρ (r)	0.060086
Laplacian of electron density<br>$\nabla^2\rho$ (r)	0.177288
Energy density<br>H(r)	-0.012450
Lagrangian kinetic energy<br>G(r)	0.056772
Potential energy density<br>V(r)	-0.069223

Table S11-c. Topological parameters of the electron density at the bond critical points of Si2-N5 and Si4-N5 bonds of **2** at M06/def2-TZVPP//BP86/D3-BJ/def2-TZVPP level of theory. All the quantities are in atomic units.

**CP (3, -1) for**		
	Si2-N5	Si4-N5
Electron density<br>ρ (r)	0.163817	0.142065
Laplacian of electron density<br>$\nabla^2\rho$ (r)	0.855100	0.658132
Energy density<br>H(r)	-0.082823	-0.066609
Lagrangian kinetic energy<br>G(r)	0.296598	0.231143
Potential energy density<br>V(r)	-0.379422	-0.297753
Ellipticity<br>ε	0.030344	0.023376

Table S12: Proton affinities corresponding to the structures shown in **Figure S16** and **Figure S17** for **1** and **2** respectively at M06/def2-TZVPP//BP86/D3-BJ/def2-TZVPP level of theory.

**1**		**2**	
**Structure**	**PA (kcal/mol)**	**Structure**	**PA (kcal/mol)**
(a)	281.0	(a)	281.2
(b)	268.7	(b)	174.3
(d)	172.1		
(e)	161.0		

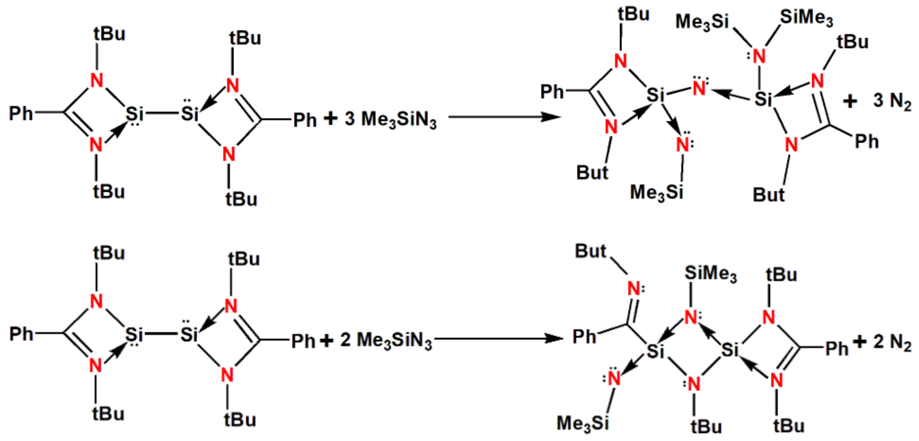

Scheme S2: Reaction for the formation of **1** (a) and **2** (b)

Table S13: The experimental and theoretical ^{29}Si NMR chemical shifts of the silicon atoms in **1**. The atom numbering is based on Figure S12.

**Atom**	**Experimental** δ (ppm)	**M06/PCM/def2-TZVPP//BP86/D3-BJ/def2-TZVPP** δ (ppm)	**B3LYP/PCM/6-311+G(2d,p)//BP86/D3-BJ/def2-TZVPP** δ (ppm)
Si1	-62.95	-61.78	-68.47
Si2	-57.20	-56.64	-59.57
Si3	-0.93	1.91	-0.73
Si4	4.25	3.45	6.21
Si5	-27.28	-24.01	-23.30

Table S14. The experimental and theoretical ^{29}Si NMR chemical shifts of the silicon atoms in **2**. The atom numbering is based on Figure S12.

**Atom**	**Experimental** δ (ppm)	**M06/PCM/def2-TZVPP//BP86/D3-BJ/def2-TZVPP** δ (ppm)	**B3LYP/PCM/6-311+G(2d,p)//BP86/D3-BJ/def2-TZVPP** δ (ppm)
Si1	-50.81	-56.81	-58.16
Si2	-70.94	-80.00	-82.81
Si3	-3.67	-0.83	-2.26
Si4	-28.48	-24.87	-27.87

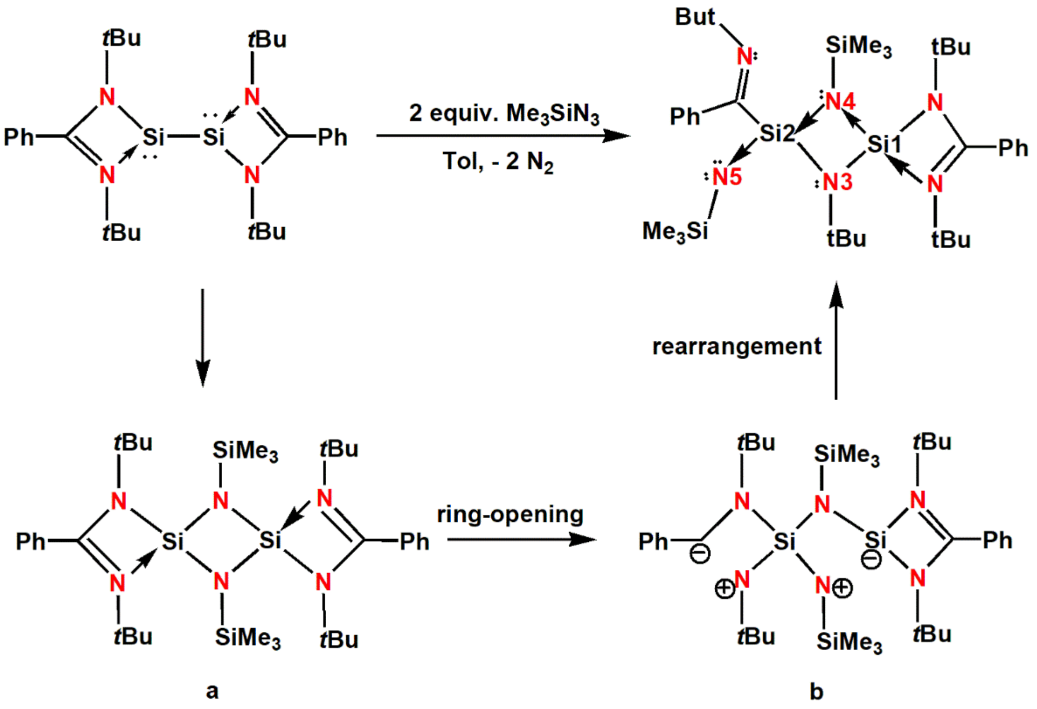

Scheme S3: Proposed reaction mechanism for the formation of **2**

Based on the structure and bonding analysis, the bonding scheme as shown in Scheme S4(b) is proposed for compound **2**. The dicoordinated nitrogen (N5) can be considered as monovalent having two lone pairs of electrons as in compound **1**. Compound **2** also shows high first (281.2 kcal/mol) and second (174.3 kcal/mol) proton affinities for the lone-pairs of N5 center (Figure S22, Table S13).

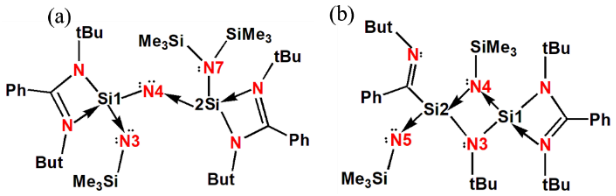

Scheme S4. Schematic representation of the qualitative bonding description of **1** (a) and **2** (b).

We have also calculated the ligating ability of the dicoordinated nitrogen center N5 in compound **2** as well. The complexation energy with respect to CO is endothermic by 19.9 kcal/mol (Figure S23, Table S15). This indicates that the ligating ability of the N5 center in compound **2** is less as compared to CO. However, dicoordinated nitrogen center N5 in **2** is relatively more reactive than dicoordinated nitrogen centers in compound **1**. The Si-Si bond in bis(silylene) is rather weak and the corresponding calculated bond dissociation energy is 43.0 kcal/mol at M06-def2-TZVPP//BP86/D3-BJ/def2-TZVPP level of theory. On the other hand, the calculated Si-N bond dissociation energies in **1** and **2** are significantly high (125.7-137.5 kcal/mol for **1** and 127.1-132.8 kcal/mol for compound **2**). Hence, there is a propensity to form a larger number of Si-N bonds on the reaction of bis(silylene) with Me_3SiN_3, and this propensity increases with the increasing equivalents of Me_3SiN_3. It is noteworthy that the total number of Si-N bonds in compound **1** is 11 while that in compound **2** is 9. Accordingly, the proposed reaction mechanism for the formation of **1** is given in Scheme S5, where three Me_3SiN groups are coordinated to the silicon centers in bis(silylene).

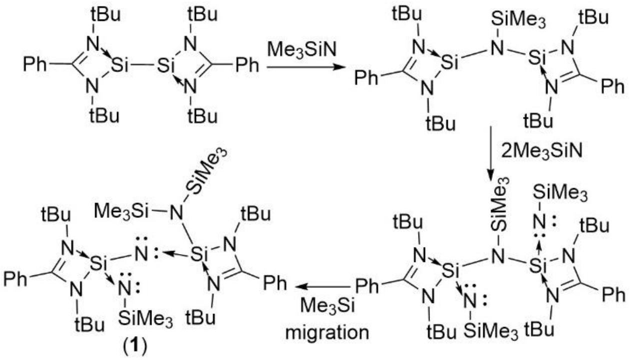

Scheme S5. Proposed reaction mechanism for the formation of **1**.

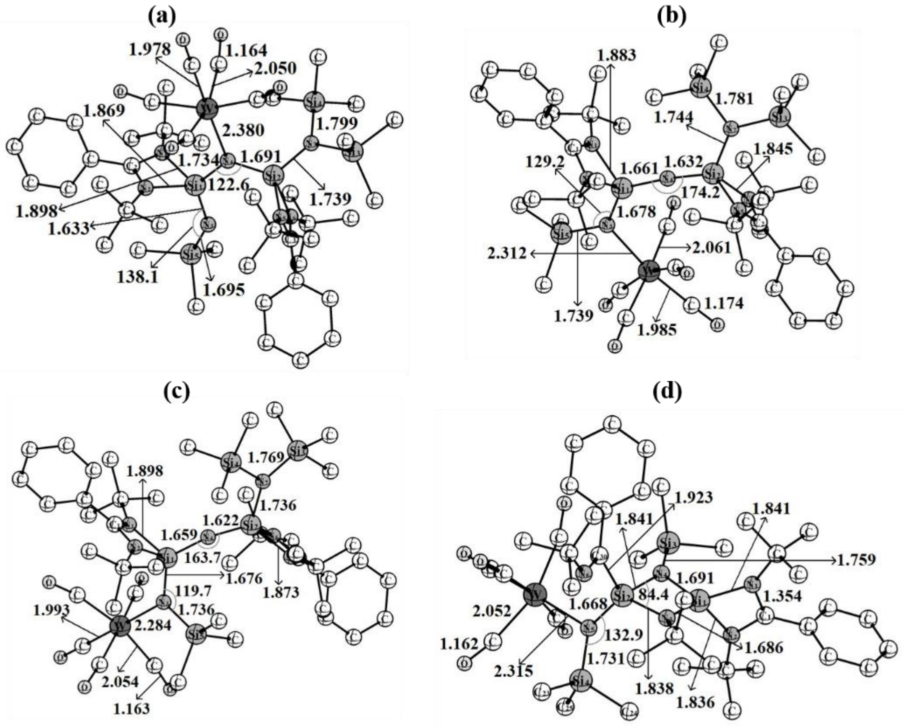

Figure S23: Equilibrium geometries of $W(CO)_5$ coordinated complexes of **1** and **2** optimized at BP86/D3-BJ/def2-TZVPP level of theory. (a) $W(CO)_5$ coordinated at N4 of **1**, (b) $W(CO)_5$ coordinated N3 of **1**, (c) $W(CO)_5$ coordinated at N3 of **1** (A second isomer of $W(CO)_5$ coordinated complex at N3 that results from the rotation around Si1-N3 bond), (d) $W(CO)_5$ coordinated at N5 of **2**. The hydrogen atoms are omitted for clarity. Distances are in Å and angles are in degrees.

Table S15: The complexation energies (**ΔE_{comp}**) calculated for the reaction of $W(CO)_6$ with **1** (**1** + $W(CO)_6$ → **1**-$W(CO)_5$ + CO) and **2** (**2** + $W(CO)_6$ → **2**-$W(CO)_5$ + CO) at M06/def2-TZVPP//BP86/D3-BJ/def2-TZVPP level of theory. The corresponding $W(CO)_5$ coordinated geometries are shown in Figure S18.

Structure	ΔE_{comp} (kcal/mol)
(a)	37.8
(b)	28.0
[a](c)	23.5
(d)	19.9

[a]A second isomer of $W(CO)_5$ coordinated complex at N3 of **1** that results from the rotation around Si1-N3 bond.

Table S16: The bond dissociation energies calculated for Si-N bonds in **1** and **2** at M06/def2-TZVPP//BP86/D3-BJ/def2-TZVPP level of theory. The atom numbering based on Figure S12.

**1**	
**Bond**	**D_e (kcal/mol)**
Si5-N3	125.7
Si1-N3	135.9
Si1-N4	135.8
Si2-N4	137.5
**2**	
**Bond**	**D_e (kcal/mol)**
Si4-N5	127.1
Si2-N5	132.8

Table S17: Optimized cartesian coordinates (BP86/D3-BJ/def2-TZVPP), electronic energy (E^{el}_{M06}, M06/def2-TZVPP//BP86/D3-BJ/def2-TZVPP), zero-point energy (ZPE_{BP86}, BP86-D3BJ/def2-TZVPP) and total energy ($E = E^{el}_{M06} + ZPE_{BP86}$) of the calculated molecules using Gaussian09 program package. The energies are given in a.u.

bis(silylene)

E^{el}_{M06} = -1968.3749042

ZPE_{BP86} = 0.687277

E (E^{el}_{M06} + ZPE_{BP86}) = -1967.687627

14	0.784107000	1.557037000	-0.979734000
7	1.480783000	-0.224446000	-1.004790000
7	2.428069000	1.427038000	-0.026679000
6	2.621279000	0.134838000	-0.363531000
6	3.845822000	-0.677627000	-0.173602000
6	3.925784000	-1.623676000	0.859776000
1	3.073338000	-1.756543000	1.526078000
6	5.082470000	-2.386834000	1.030797000
1	5.134734000	-3.117086000	1.839409000
6	6.170346000	-2.214501000	0.169734000
1	7.073175000	-2.811875000	0.302874000
6	6.098577000	-1.273426000	-0.862310000
1	6.944786000	-1.135185000	-1.536699000
6	4.944320000	-0.507795000	-1.032218000
1	4.885926000	0.233446000	-1.829864000
6	1.251998000	-1.321857000	-1.959955000
6	1.581600000	-2.693566000	-1.348210000
1	1.075255000	-2.821492000	-0.382140000
1	1.239843000	-3.490229000	-2.024650000
1	-0.490809000	-0.294946000	-2.764513000
1	3.152437000	-1.176887000	-3.032617000
6	3.130092000	2.266003000	0.957722000

6	2.494507000	3.660662000	0.860699000
1	1.417890000	3.608570000	1.082514000
1	2.960562000	4.341225000	1.586181000
1	3.322234000	0.682970000	2.451810000
6	4.635063000	2.372359000	0.656428000
1	4.796506000	2.691431000	-0.382847000
1	5.092050000	3.118681000	1.322313000
1	2.659315000	-2.822191000	-1.193053000
6	-0.246208000	-1.257861000	-2.291949000
1	-0.837506000	-1.341758000	-1.370491000
1	-0.526707000	-2.064116000	-2.983503000
6	2.076548000	-1.103161000	-3.242039000
1	1.865933000	-0.107337000	-3.656714000
1	1.824404000	-1.860667000	-3.999086000
1	2.625277000	4.077546000	-0.147810000
6	2.906076000	1.696598000	2.370198000
1	1.829810000	1.658076000	2.591499000
1	3.393931000	2.330977000	3.124634000
1	5.152823000	1.418457000	0.812330000
14	-0.784110000	1.557084000	0.979672000
7	-2.428066000	1.427039000	0.026613000
7	-1.480787000	-0.224397000	1.004811000
6	-2.621278000	0.134855000	0.363525000
6	-3.845819000	-0.677618000	0.173627000
6	-4.944318000	-0.507754000	1.032236000
1	-4.885925000	0.233519000	1.829852000
6	-6.098575000	-1.273392000	0.862357000
1	-6.944784000	-1.135124000	1.536739000
6	-6.170342000	-2.214507000	-0.169650000
1	-7.073171000	-2.811888000	-0.302767000
6	-5.082465000	-2.386874000	-1.030705000
1	-5.134728000	-3.117158000	-1.839288000
6	-3.925780000	-1.623708000	-0.859714000
1	-3.073332000	-1.756601000	-1.526009000
6	-3.130089000	2.265960000	-0.957825000
6	-4.635063000	2.372316000	-0.656543000
1	-4.796514000	2.691427000	0.382718000
1	-5.092053000	3.118607000	-1.322461000
1	-1.417897000	3.608536000	-1.082663000
1	-3.322207000	0.682864000	-2.451849000
6	-1.252003000	-1.321767000	1.960023000
6	0.246199000	-1.257747000	2.292029000
1	0.490791000	-0.294808000	2.764548000
1	0.526697000	-2.063967000	2.983624000
1	-3.152454000	-1.176762000	3.032662000
6	-1.581591000	-2.693504000	1.348333000
1	-1.075235000	-2.821467000	0.382273000
1	-1.239835000	-3.490137000	2.024809000
1	-5.152814000	1.418403000	-0.812409000
6	-2.494515000	3.660628000	-0.860855000
1	-2.625293000	4.077551000	0.147637000

1	-2.960572000	4.341158000	-1.586366000
6	-2.906060000	1.696500000	-2.370277000
1	-1.829793000	1.657981000	-2.591570000
1	-3.393919000	2.330844000	-3.124741000
1	0.837508000	-1.341685000	1.370580000
6	-2.076566000	-1.103023000	3.242090000
1	-1.865958000	-0.107182000	3.656728000
1	-1.824426000	-1.860499000	3.999169000
1	-2.659303000	-2.822142000	1.193170000

Me_3SiN_3

E^{el}_{M06} = -573.4144375

ZPE_{BP86} = 0.12085

E (E^{el}_{M06} + ZPE_{BP86}) = -573.2935875

14	0.667682000	-0.000023000	0.020995000
6	0.749800000	1.544801000	1.083212000
1	1.699466000	1.584085000	1.637839000
1	0.677691000	2.453173000	0.468683000
1	-0.067187000	1.566238000	1.819220000
6	0.749039000	-1.544618000	1.083616000
1	0.676182000	-2.453115000	0.469367000
1	1.698814000	-1.584345000	1.638020000
1	-0.067787000	-1.565289000	1.819828000
6	1.966679000	-0.000473000	-1.322427000
1	1.870945000	0.888493000	-1.961522000
1	2.976778000	-0.000076000	-0.887316000
1	1.871269000	-0.890041000	-1.960727000
7	-0.874315000	0.000272000	-0.870209000
7	-1.986184000	0.000134000	-0.360573000
7	-3.064763000	0.000016000	0.030250000

1

E^{el}_{M06} = -3360.541019

ZPE_{BP86} = 1.033565

E (E^{el}_{M06} + ZPE_{BP86}) = -3359.507454

14	1.174175000	-0.402298000	0.007163000
6	2.982854000	0.180422000	2.496550000
7	2.642474000	0.155007000	1.063718000
14	-1.491142000	0.956788000	-0.024692000
6	1.647900000	0.319507000	3.237683000
1	1.810418000	0.302943000	4.323999000
1	0.978457000	-0.508862000	2.973618000
1	1.160050000	1.264922000	2.968968000

7	2.698321000	-0.130471000	-1.077904000
6	3.880621000	1.374527000	2.860618000
1	4.891123000	1.276802000	2.447726000
1	3.967440000	1.439542000	3.954746000
1	3.439030000	2.311835000	2.494480000
6	3.435130000	0.215018000	-0.014897000
7	-2.468251000	-0.196286000	-1.089384000
6	3.651377000	-1.143274000	2.907379000
1	4.617157000	-1.271890000	2.400584000
1	3.002073000	-1.989500000	2.647817000
1	3.831918000	-1.159497000	3.992187000
7	0.130241000	0.883271000	-0.159769000
7	-2.375608000	-0.240155000	1.065875000
6	4.869320000	0.588580000	-0.029798000
6	5.855955000	-0.396163000	0.112586000
1	5.559523000	-1.440421000	0.215762000
6	7.573923000	1.300554000	-0.073172000
1	8.628554000	1.578002000	-0.090429000
6	7.204805000	-0.037929000	0.092550000
1	7.969902000	-0.807612000	0.200856000
6	6.589438000	2.283093000	-0.218478000
1	6.873661000	3.328193000	-0.346818000
6	2.983028000	-0.037075000	-2.518086000
6	5.239326000	1.930055000	-0.195723000
1	4.464862000	2.691094000	-0.295217000
6	1.973704000	-0.969123000	-3.202318000
1	0.947656000	-0.678420000	-2.949118000
1	2.118448000	-2.006762000	-2.874629000
1	2.089321000	-0.920989000	-4.293647000
6	2.754761000	1.410246000	-2.988038000
1	3.469039000	2.091672000	-2.505562000
1	1.739670000	1.735898000	-2.728569000
1	2.883482000	1.490929000	-4.077343000
6	4.404955000	-0.494475000	-2.882066000
1	4.482756000	-0.576392000	-3.975546000
1	4.621190000	-1.479966000	-2.447858000
1	5.171324000	0.211545000	-2.542993000
6	-2.380937000	-0.585181000	-2.513537000
6	-1.666898000	0.589176000	-3.200353000
1	-2.240973000	1.517886000	-3.068601000
1	-1.559518000	0.393825000	-4.275142000
1	-0.671381000	0.745240000	-2.763512000
6	-3.785507000	-0.745444000	-3.116117000
1	-4.363943000	0.183858000	-3.017882000

1	-4.336660000	-1.559808000	-2.629016000
1	-3.702312000	-0.986289000	-4.185607000
6	-1.571636000	-1.882977000	-2.682038000
1	-1.249782000	-1.992875000	-3.727250000
1	-2.174616000	-2.762389000	-2.423423000
1	-0.688674000	-1.880396000	-2.026804000
6	-2.769939000	-0.939344000	-0.008148000
6	-3.348909000	-2.297760000	-0.000121000
6	-2.479363000	-3.378383000	0.200765000
1	-1.413896000	-3.165976000	0.322014000
6	-2.988483000	-4.676994000	0.203455000
1	-2.310394000	-5.518849000	0.348696000
6	-4.357777000	-4.896436000	0.018002000
1	-4.753211000	-5.913045000	0.024595000
6	-5.221728000	-3.814042000	-0.178106000
1	-6.289573000	-3.983868000	-0.320480000
6	-4.719432000	-2.510655000	-0.193059000
1	-5.385512000	-1.659600000	-0.338452000
6	-2.455681000	-0.540284000	2.508496000
6	-3.899096000	-0.851754000	2.933781000
1	-4.567775000	-0.004650000	2.726234000
1	-3.923189000	-1.047205000	4.015206000
1	-4.285995000	-1.741297000	2.421532000
6	-1.988915000	0.745564000	3.206487000
1	-0.964901000	0.999798000	2.905425000
1	-2.005822000	0.613699000	4.296224000
1	-2.642303000	1.588391000	2.941976000
6	-1.529537000	-1.718237000	2.869351000
1	-0.600306000	-1.679096000	2.282084000
1	-2.014212000	-2.676748000	2.644734000
1	-1.296028000	-1.700759000	3.943265000
7	-2.127709000	2.555964000	0.065440000
14	-0.878379000	3.835096000	0.063415000
6	-1.635849000	5.533947000	0.405348000
1	-0.830931000	6.269830000	0.249578000
1	-1.972849000	5.636000000	1.445684000
1	-2.464665000	5.815103000	-0.256136000
6	-0.071681000	3.913537000	-1.629750000
1	-0.816025000	4.115672000	-2.413838000
1	0.405004000	2.948437000	-1.839524000
1	0.694948000	4.701449000	-1.675467000
6	0.393594000	3.595735000	1.426248000
1	1.019843000	4.496941000	1.517180000
1	1.024951000	2.727398000	1.205931000

1	-0.101677000	3.436037000	2.395758000
14	-3.841156000	2.960133000	-0.051807000
6	-4.981148000	1.460292000	-0.071858000
1	-6.013967000	1.840650000	-0.095241000
1	-4.873960000	0.840388000	0.825988000
1	-4.833560000	0.824767000	-0.951492000
6	-4.403353000	3.975629000	1.435016000
1	-4.055784000	5.014771000	1.416488000
1	-4.044069000	3.517409000	2.368747000
1	-5.503143000	3.990381000	1.474413000
6	-4.167547000	3.876492000	-1.668152000
1	-3.548622000	4.774679000	-1.792597000
1	-5.222261000	4.183941000	-1.735646000
1	-3.959992000	3.212223000	-2.520949000
7	0.676682000	-1.948464000	0.178789000
14	1.407101000	-3.469867000	0.102529000
6	1.042596000	-4.459611000	1.682352000
1	1.466788000	-3.955206000	2.564503000
1	1.462023000	-5.476851000	1.642760000
1	-0.041203000	-4.548687000	1.854021000
6	0.741623000	-4.493844000	-1.353335000
1	0.970576000	-4.002343000	-2.311155000
1	-0.350657000	-4.613517000	-1.306768000
1	1.189566000	-5.499574000	-1.378217000
6	3.300912000	-3.478488000	-0.108367000
1	3.595951000	-2.984977000	-1.046137000
1	3.694441000	-4.506775000	-0.131978000
1	3.797317000	-2.950459000	0.718519000

2

E^{el}_{M06} = -2896.50025

ZPE_{BP86} = 0.914192

E (E^{el}_{M06} + ZPE_{BP86}) = -2895.586058

14	-1.076415000	-0.015458000	-0.082445000
6	-3.352665000	0.013666000	-0.187615000
6	-4.826034000	0.044057000	-0.281522000
6	-5.614876000	-0.169187000	0.857305000
1	-5.136431000	-0.341234000	1.821377000
6	-7.005959000	-0.130476000	0.752952000
1	-7.618001000	-0.287252000	1.641476000
6	-7.611392000	0.110717000	-0.483986000
1	-8.698611000	0.136241000	-0.562629000

6	-6.823543000	0.319513000	-1.619929000
1	-7.292915000	0.503040000	-2.586741000
6	-5.431873000	0.292247000	-1.521136000
1	-4.810863000	0.441977000	-2.404326000
7	-2.524821000	1.082353000	-0.182681000
6	-2.768073000	2.537820000	-0.108625000
6	-3.125674000	3.071099000	-1.505667000
1	-3.227170000	4.164862000	-1.477019000
1	-4.077053000	2.649588000	-1.856866000
1	-2.339458000	2.816551000	-2.229198000
6	-3.879077000	2.869054000	0.898605000
1	-3.928331000	3.957933000	1.035110000
1	-3.662851000	2.411072000	1.873713000
1	-4.863840000	2.526663000	0.558369000
6	-1.441646000	3.144799000	0.368825000
1	-1.510188000	4.240716000	0.367247000
1	-0.595081000	2.846236000	-0.266521000
1	-1.200818000	2.808753000	1.385939000
7	-2.567574000	-1.073482000	-0.116436000
6	-2.845341000	-2.525423000	-0.157340000
6	-1.478108000	-3.206563000	-0.318058000
1	-0.995295000	-2.913151000	-1.258802000
1	-1.601451000	-4.296887000	-0.316114000
1	-0.790947000	-2.936831000	0.495406000
6	-3.753617000	-2.874478000	-1.345713000
1	-4.756370000	-2.442414000	-1.235641000
1	-3.859262000	-3.965707000	-1.415389000
1	-3.314596000	-2.511691000	-2.285152000
6	-3.490118000	-2.966502000	1.167533000
1	-2.866005000	-2.662964000	2.018638000
1	-3.588135000	-4.060664000	1.187156000
1	-4.491795000	-2.533996000	1.289002000
7	0.134597000	-0.134050000	-1.232302000
6	0.259742000	-0.001102000	-2.682273000
6	0.550853000	1.467900000	-3.040601000
1	0.681811000	1.599473000	-4.125112000
1	-0.275040000	2.113627000	-2.707833000
1	1.459830000	1.809967000	-2.530230000
6	1.413691000	-0.893096000	-3.163507000
1	1.537204000	-0.819243000	-4.253515000
1	2.359394000	-0.596745000	-2.687963000
1	1.227651000	-1.941202000	-2.894016000
6	-1.055132000	-0.440947000	-3.345080000
1	-1.004278000	-0.336745000	-4.438357000

1	-1.271779000	-1.491792000	-3.107481000
1	-1.892992000	0.177424000	-2.984221000
7	-0.031286000	-0.085691000	1.218513000
14	-0.070527000	-0.374571000	2.926056000
6	0.118359000	-2.208815000	3.281259000
1	0.282890000	-2.414749000	4.349313000
1	-0.759227000	-2.787131000	2.956697000
1	0.986398000	-2.568495000	2.707327000
6	1.355768000	0.568037000	3.698700000
1	1.366426000	0.487669000	4.795409000
1	2.305885000	0.163346000	3.318792000
1	1.312381000	1.629979000	3.419437000
6	-1.735039000	0.265570000	3.553861000
1	-1.865817000	0.085516000	4.630965000
1	-1.829858000	1.347545000	3.378335000
1	-2.564963000	-0.236146000	3.031086000
14	1.375474000	-0.372453000	0.095173000
7	2.150352000	-1.763610000	0.316818000
14	3.065873000	-3.123170000	-0.024008000
6	4.163749000	-2.944848000	-1.561302000
1	4.837705000	-3.808032000	-1.680906000
1	3.555661000	-2.869811000	-2.475174000
1	4.781060000	-2.037642000	-1.496197000
6	1.962591000	-4.643610000	-0.324532000
1	2.546750000	-5.553865000	-0.532951000
1	1.328823000	-4.841718000	0.553679000
1	1.295430000	-4.468969000	-1.183358000
6	4.197336000	-3.548796000	1.438599000
1	4.755517000	-4.483815000	1.275170000
1	4.929429000	-2.745164000	1.608106000
1	3.608643000	-3.662055000	2.362005000
7	1.962143000	2.298418000	0.421333000
6	2.665830000	3.596617000	0.564118000
6	4.142449000	3.545615000	0.998421000
1	4.472576000	4.559614000	1.270301000
1	4.264793000	2.901947000	1.880540000
1	4.806355000	3.173845000	0.211447000
6	1.872441000	4.356822000	1.644894000
1	2.266788000	5.375163000	1.775676000
1	0.812015000	4.417535000	1.369667000
1	1.943858000	3.832217000	2.608522000
6	2.523962000	4.334293000	-0.779544000
1	2.859405000	5.377457000	-0.681640000
1	3.130474000	3.848171000	-1.555270000

1	1.475055000	4.335204000	-1.108403000
6	2.505154000	1.175703000	0.107948000
6	3.920331000	0.887402000	-0.272426000
6	4.751060000	0.154547000	0.590328000
1	4.361360000	-0.162432000	1.557144000
6	6.053824000	-0.168653000	0.212400000
1	6.691753000	-0.728933000	0.897488000
6	6.533585000	0.196202000	-1.050311000
1	7.546956000	-0.072699000	-1.350928000
6	5.698376000	0.885053000	-1.933467000
1	6.055940000	1.154059000	-2.928674000
6	4.400500000	1.228697000	-1.545884000
1	3.749037000	1.760744000	-2.240894000

One proton added at N3 of **1** (Figure S16-a)
E^{el}_{M06} = -3361.001556
ZPE_{BP86} = 1.046269
E (E^{el}_{M06} + ZPE_{BP86}) = -3359.955287

14	1.300070000	0.381887000	0.003448000
6	3.070835000	-0.173863000	-2.492196000
7	2.742792000	-0.085539000	-1.049608000
14	-1.453687000	-0.942146000	0.054106000
6	1.721095000	-0.203203000	-3.217281000
1	1.873069000	-0.223254000	-4.303891000
1	1.132015000	0.691015000	-2.970190000
1	1.150613000	-1.095067000	-2.929644000
7	2.791157000	0.320660000	1.074231000
6	3.850413000	-1.458138000	-2.812290000
1	4.861014000	-1.440393000	-2.389274000
1	3.942415000	-1.562497000	-3.901955000
1	3.319064000	-2.338482000	-2.426779000
6	3.545424000	-0.072005000	0.026911000
7	-2.491252000	0.164372000	1.110742000
6	3.864814000	1.065836000	-2.933104000
1	4.826426000	1.130158000	-2.407849000
1	3.291406000	1.982515000	-2.740606000
1	4.072219000	1.013579000	-4.010653000
7	0.163837000	-0.764077000	0.235798000
7	-2.315590000	0.275789000	-1.041740000
6	4.977722000	-0.435955000	0.065329000
6	5.963230000	0.556499000	-0.020420000
1	5.672523000	1.603229000	-0.108407000
6	7.677113000	-1.143179000	0.168754000

1	8.730970000	-1.419008000	0.210109000
6	7.311373000	0.198676000	0.027112000
1	8.077554000	0.971135000	-0.040513000
6	6.692524000	-2.131892000	0.256869000
1	6.975478000	-3.179060000	0.365053000
6	3.034760000	0.186745000	2.530894000
6	5.342524000	-1.782838000	0.201196000
1	4.569959000	-2.550157000	0.254332000
6	1.953381000	1.039041000	3.204859000
1	0.953453000	0.684722000	2.924802000
1	2.043225000	2.093885000	2.911529000
1	2.043741000	0.973127000	4.296475000
6	2.868890000	-1.286235000	2.938316000
1	3.636741000	-1.911797000	2.463972000
1	1.881032000	-1.657702000	2.636428000
1	2.965809000	-1.394961000	4.027286000
6	4.420009000	0.707685000	2.938206000
1	4.475926000	0.742849000	4.034600000
1	4.590162000	1.723624000	2.556374000
1	5.229680000	0.061918000	2.582055000
6	-2.418588000	0.549660000	2.543230000
6	-1.678010000	-0.607658000	3.229687000
1	-2.237029000	-1.546590000	3.110817000
1	-1.567722000	-0.405709000	4.302481000
1	-0.680730000	-0.749310000	2.791682000
6	-3.815717000	0.692477000	3.163660000
1	-4.390586000	-0.237489000	3.062759000
1	-4.382398000	1.511656000	2.706122000
1	-3.714344000	0.911497000	4.235504000
6	-1.633609000	1.862693000	2.693322000
1	-1.412329000	2.054662000	3.751923000
1	-2.209879000	2.714712000	2.310785000
1	-0.686518000	1.812646000	2.141116000
6	-2.842926000	0.893091000	0.034881000
6	-3.620041000	2.150530000	0.031786000
6	-2.980242000	3.365244000	-0.255494000
1	-1.909453000	3.381997000	-0.455168000
6	-3.706116000	4.556160000	-0.258010000
1	-3.199320000	5.498105000	-0.468644000
6	-5.078421000	4.538427000	0.008506000
1	-5.647475000	5.468180000	-0.001244000
6	-5.720058000	3.328319000	0.288922000
1	-6.790564000	3.311168000	0.493882000
6	-4.994396000	2.136321000	0.310601000

1	-5.490493000	1.189838000	0.522007000
6	-2.550415000	0.461180000	-2.495050000
6	-4.041725000	0.627131000	-2.819775000
1	-4.617142000	-0.245665000	-2.485159000
1	-4.166335000	0.715060000	-3.907531000
1	-4.465395000	1.527501000	-2.359451000
6	-2.019055000	-0.815181000	-3.161803000
1	-0.956688000	-0.963468000	-2.926733000
1	-2.121375000	-0.742876000	-4.251922000
1	-2.570394000	-1.697971000	-2.814242000
6	-1.762833000	1.680601000	-3.003803000
1	-0.707312000	1.614199000	-2.709560000
1	-2.179274000	2.615102000	-2.607997000
1	-1.810249000	1.728693000	-4.100099000
7	-2.004740000	-2.551362000	-0.077446000
14	-0.717197000	-3.808040000	-0.090318000
6	-1.392138000	-5.495455000	-0.588876000
1	-0.570951000	-6.212468000	-0.429915000
1	-1.644707000	-5.529586000	-1.657496000
1	-2.253734000	-5.862739000	-0.019403000
6	0.008868000	-3.930063000	1.635063000
1	-0.761632000	-4.206860000	2.368754000
1	0.431156000	-2.960605000	1.928325000
1	0.807400000	-4.684895000	1.682817000
6	0.610924000	-3.445341000	-1.371891000
1	1.243029000	-4.337378000	-1.499733000
1	1.234732000	-2.598531000	-1.068105000
1	0.161042000	-3.219948000	-2.350279000
14	-3.702231000	-3.066510000	0.062130000
6	-4.910024000	-1.634361000	0.254139000
1	-5.920700000	-2.070381000	0.237635000
1	-4.857092000	-0.908673000	-0.566251000
1	-4.790771000	-1.100363000	1.202214000
6	-4.261310000	-3.968290000	-1.489124000
1	-3.704744000	-4.888834000	-1.692868000
1	-4.171432000	-3.317101000	-2.371602000
1	-5.324308000	-4.235065000	-1.388819000
6	-3.895791000	-4.110867000	1.614266000
1	-3.231841000	-4.983482000	1.643182000
1	-4.929956000	-4.474460000	1.706718000
1	-3.678728000	-3.500799000	2.504161000
7	0.756631000	1.978675000	-0.274563000
14	1.425901000	3.605773000	-0.226152000
6	1.030265000	4.441717000	-1.861423000

1	1.492972000	3.908751000	-2.704692000
1	1.390773000	5.480611000	-1.879959000
1	-0.054741000	4.467730000	-2.045018000
6	0.626962000	4.561429000	1.181084000
1	0.840405000	4.092543000	2.152345000
1	-0.466643000	4.608044000	1.072843000
1	0.999028000	5.596078000	1.217883000
6	3.277841000	3.495654000	0.045080000
1	3.514003000	3.003923000	0.997718000
1	3.709874000	4.506473000	0.069316000
1	3.774210000	2.939942000	-0.761375000
1	-0.243273000	1.956088000	-0.482656000

One proton added at N4 of **1** (Figure S16-b)
E^{el}_{M06} = -3360.982109
ZPE_{BP86} = 1.046388
E (E^{el}_{M06} + ZPE_{BP86}) = -3359.935721

14	1.269218000	0.496817000	-0.025122000
6	3.038548000	-0.137339000	-2.503970000
7	2.695161000	-0.077583000	-1.064752000
14	-1.524548000	-0.966047000	0.000679000
6	1.702466000	-0.269646000	-3.242763000
1	1.866442000	-0.261720000	-4.327893000
1	1.037547000	0.566703000	-2.990359000
1	1.203750000	-1.211324000	-2.977495000
7	2.707118000	0.124246000	1.087997000
6	3.918972000	-1.352406000	-2.832138000
1	4.927432000	-1.262104000	-2.414210000
1	4.013091000	-1.440645000	-3.923076000
1	3.463436000	-2.278388000	-2.453544000
6	3.473188000	-0.187167000	0.028430000
7	-2.370633000	0.213574000	1.083568000
6	3.727741000	1.167523000	-2.934011000
1	4.687097000	1.297136000	-2.417165000
1	3.086948000	2.031183000	-2.712787000
1	3.925313000	1.151085000	-4.014730000
7	0.174047000	-0.857767000	0.079484000
7	-2.291339000	0.264805000	-1.076327000
6	4.901072000	-0.567971000	0.055182000
6	5.889275000	0.407016000	-0.135871000
1	5.601533000	1.448307000	-0.282173000
6	7.596983000	-1.291610000	0.114195000
1	8.649703000	-1.573865000	0.137020000

6	7.235609000	0.041124000	-0.105245000
1	8.004571000	0.800425000	-0.248621000
6	6.609411000	-2.262453000	0.307610000
1	6.889412000	-3.302049000	0.478717000
6	2.988139000	0.100605000	2.540073000
6	5.261237000	-1.903449000	0.279753000
1	4.486156000	-2.657519000	0.419699000
6	2.022626000	1.110320000	3.174580000
1	0.983308000	0.883539000	2.908702000
1	2.240319000	2.130034000	2.832722000
1	2.112458000	1.080244000	4.268119000
6	2.697270000	-1.312509000	3.073917000
1	3.359999000	-2.051117000	2.602647000
1	1.655346000	-1.596359000	2.870291000
1	2.854542000	-1.355556000	4.160416000
6	4.428494000	0.510325000	2.878575000
1	4.507623000	0.642702000	3.966074000
1	4.688648000	1.463588000	2.400010000
1	5.163226000	-0.244652000	2.578879000
6	-2.317629000	0.571397000	2.524586000
6	-1.523915000	-0.562990000	3.189204000
1	-2.010248000	-1.533280000	3.010477000
1	-1.471113000	-0.400944000	4.272798000
1	-0.499217000	-0.613732000	2.799154000
6	-3.730088000	0.611425000	3.125779000
1	-4.235180000	-0.359260000	3.027618000
1	-4.344389000	1.384149000	2.648040000
1	-3.662953000	0.851129000	4.195765000
6	-1.610023000	1.921860000	2.713320000
1	-1.313763000	2.044417000	3.763745000
1	-2.273413000	2.756120000	2.454402000
1	-0.721077000	1.986349000	2.070510000
6	-2.691360000	0.961717000	0.005259000
6	-3.335820000	2.285008000	0.003727000
6	-2.543808000	3.407381000	-0.270808000
1	-1.479233000	3.257336000	-0.446782000
6	-3.124185000	4.675288000	-0.266324000
1	-2.505204000	5.550802000	-0.464346000
6	-4.490645000	4.820651000	-0.006829000
1	-4.943661000	5.812346000	-0.009928000
6	-5.278946000	3.696048000	0.257809000
1	-6.345228000	3.808755000	0.454372000
6	-4.704660000	2.423750000	0.272839000
1	-5.317390000	1.543468000	0.466042000

6	-2.430528000	0.528689000	-2.529946000
6	-3.891710000	0.805487000	-2.909194000
1	-4.535914000	-0.055865000	-2.686476000
1	-3.950282000	0.998029000	-3.989050000
1	-4.284739000	1.687485000	-2.389685000
6	-1.960243000	-0.763639000	-3.211207000
1	-0.919031000	-0.989431000	-2.946044000
1	-2.016919000	-0.657465000	-4.301517000
1	-2.588382000	-1.613840000	-2.911150000
6	-1.539605000	1.714611000	-2.940308000
1	-0.584226000	1.689688000	-2.399996000
1	-2.025384000	2.668484000	-2.702247000
1	-1.356589000	1.689607000	-4.022922000
7	-2.091717000	-2.567197000	-0.053414000
14	-0.917082000	-3.922019000	-0.122427000
6	-1.725505000	-5.595015000	-0.378482000
1	-0.932670000	-6.344506000	-0.227414000
1	-2.103603000	-5.722856000	-1.400377000
1	-2.532180000	-5.831412000	0.325000000
6	0.005643000	-3.989604000	1.516221000
1	-0.695290000	-4.254605000	2.321768000
1	0.478439000	-3.042320000	1.807519000
1	0.789584000	-4.760747000	1.491366000
6	0.249201000	-3.702764000	-1.583888000
1	0.875239000	-4.602418000	-1.681236000
1	0.918456000	-2.836020000	-1.526122000
1	-0.334438000	-3.607085000	-2.511264000
14	-3.840739000	-2.947171000	0.106375000
6	-4.929758000	-1.416219000	0.113295000
1	-5.967740000	-1.780249000	0.157941000
1	-4.834477000	-0.817978000	-0.800046000
1	-4.769098000	-0.767913000	0.980120000
6	-4.416693000	-3.966697000	-1.363623000
1	-4.157838000	-5.029117000	-1.300285000
1	-4.001487000	-3.571911000	-2.302766000
1	-5.512288000	-3.897618000	-1.435463000
6	-4.103823000	-3.813169000	1.752439000
1	-3.489250000	-4.713496000	1.880198000
1	-5.156574000	-4.112283000	1.864763000
1	-3.865347000	-3.129126000	2.580891000
7	0.572332000	1.945585000	-0.129479000
14	1.285370000	3.504516000	-0.110040000
6	0.911708000	4.399493000	-1.733042000
1	1.311708000	3.838580000	-2.591449000

1	1.360258000	5.403950000	-1.753077000
1	-0.169428000	4.515817000	-1.900204000
6	0.595531000	4.537632000	1.314756000
1	0.841561000	4.085279000	2.287070000
1	-0.498322000	4.635287000	1.269727000
1	1.021722000	5.552167000	1.305022000
6	3.172173000	3.499335000	0.102233000
1	3.472025000	3.050447000	1.060573000
1	3.568310000	4.525887000	0.082309000
1	3.670705000	2.940446000	-0.702490000
1	0.653761000	-1.747429000	0.205052000

Two protons added at N3 of **1** (Figure S16-c)
E^{el}_{M06} = -3361.287773
ZPE_{BP86} = 1.058164
E (E^{el}_{M06} + ZPE_{BP86}) = -3360.229609

14	-1.375365000	-0.386807000	-0.065786000
6	-3.101058000	0.156578000	-2.577476000
7	-2.771554000	0.081614000	-1.119510000
14	1.565424000	0.872548000	0.053965000
6	-1.766301000	0.007393000	-3.317265000
1	-1.913231000	0.170212000	-4.391718000
1	-1.347482000	-1.006770000	-3.213782000
1	-1.028261000	0.737008000	-2.958551000
7	-2.818095000	-0.359769000	1.011340000
6	-3.723283000	1.513603000	-2.937683000
1	-4.724217000	1.624668000	-2.508112000
1	-3.819103000	1.585054000	-4.029055000
1	-3.089872000	2.340741000	-2.594243000
6	-3.586973000	0.035531000	-0.039097000
7	2.685436000	-0.139239000	1.086223000
6	-4.047484000	-0.988378000	-2.963068000
1	-5.005786000	-0.914734000	-2.434727000
1	-3.597061000	-1.967443000	-2.742905000
1	-4.253306000	-0.951680000	-4.040741000
7	-0.011133000	0.388269000	0.190809000
7	2.540254000	-0.206010000	-1.067800000
6	-5.015850000	0.384052000	-0.001022000
6	-5.992973000	-0.608074000	0.172612000
1	-5.703954000	-1.655535000	0.246826000
6	-7.715268000	1.091697000	0.110941000
1	-8.768620000	1.368198000	0.155225000
6	-7.341500000	-0.251002000	0.215762000

1	-8.100645000	-1.023963000	0.333915000
6	-6.740261000	2.081448000	-0.050527000
1	-7.030403000	3.129412000	-0.124132000
6	-3.075286000	-0.205201000	2.482235000
6	-5.392212000	1.732944000	-0.118511000
1	-4.629136000	2.502382000	-0.231341000
6	-1.711146000	-0.334634000	3.165607000
1	-1.040498000	0.480244000	2.868003000
1	-1.224686000	-1.288169000	2.917455000
1	-1.838207000	-0.297963000	4.254240000
6	-3.664730000	1.178297000	2.791441000
1	-4.694927000	1.279376000	2.433479000
1	-3.050554000	1.972512000	2.345887000
1	-3.674081000	1.328378000	3.878812000
6	-4.012250000	-1.317532000	2.970904000
1	-4.163075000	-1.217917000	4.053833000
1	-3.585251000	-2.312249000	2.783562000
1	-4.996007000	-1.255753000	2.490248000
6	2.706007000	-0.518366000	2.520518000
6	2.067570000	0.662865000	3.262223000
1	2.667451000	1.573209000	3.136459000
1	1.996101000	0.443939000	4.334739000
1	1.054927000	0.864235000	2.885646000
6	4.134367000	-0.736522000	3.035957000
1	4.761028000	0.144413000	2.843143000
1	4.607448000	-1.613435000	2.580484000
1	4.105137000	-0.896522000	4.122013000
6	1.860059000	-1.787991000	2.712574000
1	1.758492000	-2.028010000	3.779318000
1	2.328150000	-2.648077000	2.214808000
1	0.856304000	-1.630849000	2.294735000
6	3.117256000	-0.802884000	-0.005386000
6	4.012109000	-1.976734000	-0.038016000
6	3.471130000	-3.254954000	-0.239206000
1	2.391547000	-3.378041000	-0.343039000
6	4.309418000	-4.369442000	-0.291875000
1	3.886447000	-5.363118000	-0.440027000
6	5.691491000	-4.209073000	-0.155941000
1	6.347515000	-5.078083000	-0.203912000
6	6.231991000	-2.934904000	0.042951000
1	7.309465000	-2.808341000	0.147440000
6	5.397456000	-1.818629000	0.109095000
1	5.816938000	-0.824179000	0.257154000
6	2.705743000	-0.403004000	-2.526627000
6	4.153293000	-0.716648000	-2.926957000
1	4.846230000	0.044897000	-2.546951000
1	4.225658000	-0.717587000	-4.022585000
1	4.481631000	-1.698316000	-2.568359000
6	2.263696000	0.916377000	-3.175051000
1	1.244491000	1.188433000	-2.862535000
1	2.275066000	0.825907000	-4.268427000

1	2.934917000	1.735543000	-2.888334000
6	1.779845000	-1.545475000	-2.980821000
1	0.738190000	-1.302999000	-2.729173000
1	2.063676000	-2.493073000	-2.503450000
1	1.839807000	-1.682664000	-4.068669000
7	1.856291000	2.539143000	-0.004401000
14	0.433728000	3.635529000	0.092983000
6	0.890680000	5.440671000	-0.128021000
1	-0.020766000	6.016281000	0.098841000
1	1.169102000	5.673838000	-1.163477000
1	1.677369000	5.814796000	0.536668000
6	-0.373069000	3.414957000	1.773029000
1	0.328185000	3.672194000	2.579640000
1	-0.687939000	2.373277000	1.914708000
1	-1.254542000	4.064151000	1.879482000
6	-0.778031000	3.260925000	-1.297663000
1	-1.564115000	4.031017000	-1.310464000
1	-1.253229000	2.281728000	-1.178137000
1	-0.276212000	3.291493000	-2.275950000
14	3.503260000	3.260401000	0.056748000
6	4.852720000	1.952719000	-0.028304000
1	5.809914000	2.495805000	-0.014639000
1	4.831271000	1.361649000	-0.951748000
1	4.847563000	1.274508000	0.832352000
6	3.768053000	4.389124000	-1.419723000
1	3.340510000	5.389443000	-1.287424000
1	3.342614000	3.960655000	-2.338735000
1	4.848302000	4.515225000	-1.585118000
6	3.724903000	4.144175000	1.697604000
1	2.924057000	4.856431000	1.931296000
1	4.672633000	4.703198000	1.695917000
1	3.776197000	3.419784000	2.523717000
7	-0.992913000	-2.148236000	-0.603590000
14	-1.706624000	-3.796442000	0.007248000
6	-0.992203000	-5.004314000	-1.219404000
1	-1.336021000	-4.807440000	-2.245550000
1	-1.309115000	-6.026519000	-0.963889000
1	0.107649000	-4.996927000	-1.216998000
6	-1.037021000	-4.011806000	1.727024000
1	-1.427143000	-3.263302000	2.428454000
1	0.060840000	-3.972883000	1.752858000
1	-1.336952000	-5.000944000	2.105189000
6	-3.555597000	-3.644806000	-0.094055000
1	-3.937582000	-2.910390000	0.624789000
1	-4.006418000	-4.618111000	0.151509000
1	-3.898963000	-3.370994000	-1.100397000
1	0.029727000	-2.184889000	-0.473815000
1	-1.110860000	-2.147858000	-1.628103000

Two protons added at N4 of **1** (Figure S16-d)

E^{el}_{M06} = -3361.250683
ZPE_{BP86} = 1.058262
E (E^{el}_{M06} + ZPE_{BP86}) = -3360.192421

14	1.330527000	-0.698387000	0.027135000
6	3.007037000	-0.016150000	2.529162000
7	2.699679000	-0.104301000	1.075307000
14	-1.583265000	1.011843000	0.053641000
6	1.653565000	0.198950000	3.216114000
1	1.767793000	0.163437000	4.306401000
1	0.939177000	-0.584940000	2.927477000
1	1.240715000	1.187504000	2.964249000
7	2.684825000	-0.197276000	-1.091705000
6	3.934081000	1.162232000	2.852845000
1	4.952386000	1.009239000	2.481781000
1	3.988372000	1.277148000	3.943474000
1	3.545980000	2.100861000	2.432651000
6	3.465114000	0.089383000	-0.021879000
7	-2.303105000	-0.157725000	-1.070986000
6	3.611931000	-1.346594000	3.001792000
1	4.576677000	-1.537240000	2.516134000
1	2.934777000	-2.182851000	2.781899000
1	3.781473000	-1.316633000	4.086329000
7	0.250494000	0.878175000	0.036190000
7	-2.280084000	-0.242285000	1.098797000
6	4.869140000	0.526742000	-0.044991000
6	5.886479000	-0.374119000	0.302333000
1	5.638940000	-1.403254000	0.561657000
6	7.529847000	1.360717000	-0.087985000
1	8.569909000	1.686061000	-0.105455000
6	7.216139000	0.045978000	0.271578000
1	8.009825000	-0.655977000	0.526808000
6	6.512816000	2.258118000	-0.428734000
1	6.757625000	3.283161000	-0.706677000
6	3.021973000	-0.286733000	-2.543217000
6	5.180825000	1.842648000	-0.416484000
1	4.384223000	2.540335000	-0.677109000
6	2.061888000	-1.319737000	-3.147097000
1	1.013609000	-1.074190000	-2.937752000
1	2.256319000	-2.322641000	-2.747783000
1	2.191506000	-1.350971000	-4.236032000
6	2.795539000	1.091552000	-3.182789000
1	3.458406000	1.845792000	-2.739374000
1	1.752563000	1.420812000	-3.059143000

1	3.001527000	1.049770000	-4.260413000
6	4.463581000	-0.757187000	-2.782238000
1	4.582701000	-0.974465000	-3.851801000
1	4.676081000	-1.679303000	-2.225445000
1	5.208011000	-0.001708000	-2.511224000
6	-2.240658000	-0.456082000	-2.532367000
6	-1.293118000	0.606758000	-3.105179000
1	-1.658004000	1.621590000	-2.885775000
1	-1.219042000	0.505086000	-4.194474000
1	-0.276265000	0.493858000	-2.702048000
6	-3.621958000	-0.292300000	-3.179590000
1	-4.011085000	0.726690000	-3.051206000
1	-4.341481000	-1.007704000	-2.765693000
1	-3.540598000	-0.487635000	-4.257035000
6	-1.691716000	-1.869197000	-2.762347000
1	-1.433312000	-1.993638000	-3.822026000
1	-2.438182000	-2.631834000	-2.511354000
1	-0.802248000	-2.047381000	-2.143542000
6	-2.666616000	-0.925949000	-0.009008000
6	-3.377477000	-2.205415000	-0.051326000
6	-2.712350000	-3.375713000	0.340085000
1	-1.668701000	-3.310744000	0.636096000
6	-3.381869000	-4.596726000	0.285751000
1	-2.859079000	-5.510557000	0.568625000
6	-4.716121000	-4.649183000	-0.130408000
1	-5.239628000	-5.604678000	-0.162749000
6	-5.382070000	-3.477142000	-0.504879000
1	-6.425349000	-3.515904000	-0.817668000
6	-4.714959000	-2.252604000	-0.479865000
1	-5.237733000	-1.336340000	-0.751444000
6	-2.520522000	-0.484153000	2.552970000
6	-4.012064000	-0.696935000	2.839750000
1	-4.603176000	0.196162000	2.599219000
1	-4.140993000	-0.902934000	3.910481000
1	-4.414544000	-1.552473000	2.285103000
6	-2.036476000	0.793904000	3.249671000
1	-0.962946000	0.958281000	3.075983000
1	-2.182457000	0.708457000	4.333078000
1	-2.591866000	1.674545000	2.898184000
6	-1.706733000	-1.700534000	3.023127000
1	-0.713612000	-1.714584000	2.557438000
1	-2.210720000	-2.636247000	2.756485000
1	-1.599640000	-1.676218000	4.115301000
7	-1.961737000	2.641400000	0.098942000

14	-0.705330000	3.942719000	0.089217000
6	-1.418215000	5.627050000	0.453078000
1	-0.595321000	6.346826000	0.318619000
1	-1.758215000	5.712885000	1.493045000
1	-2.232150000	5.941094000	-0.209257000
6	0.110420000	3.957467000	-1.604874000
1	-0.633944000	4.167182000	-2.386361000
1	0.622911000	3.027163000	-1.898810000
1	0.867181000	4.754941000	-1.647518000
6	0.583509000	3.626473000	1.442409000
1	0.969783000	4.604152000	1.768072000
1	1.473906000	3.057922000	1.130326000
1	0.149845000	3.158465000	2.338949000
14	-3.714533000	3.135927000	-0.077178000
6	-4.822435000	1.622927000	-0.133356000
1	-5.853103000	2.008143000	-0.174060000
1	-4.754776000	0.994002000	0.761568000
1	-4.675488000	1.000239000	-1.021506000
6	-4.228287000	4.139022000	1.416009000
1	-3.921750000	5.189657000	1.375108000
1	-3.835241000	3.704858000	2.346861000
1	-5.326027000	4.121913000	1.487201000
6	-3.882008000	4.027219000	-1.714846000
1	-3.258003000	4.924803000	-1.808572000
1	-4.926510000	4.343201000	-1.856286000
1	-3.630148000	3.354548000	-2.548482000
7	0.440399000	-2.021906000	0.091068000
14	1.081407000	-3.645987000	0.107507000
6	0.666159000	-4.454310000	1.757297000
1	1.079917000	-3.875959000	2.597117000
1	1.096340000	-5.465298000	1.810775000
1	-0.414936000	-4.551792000	1.930484000
6	0.333844000	-4.629680000	-1.309330000
1	0.627289000	-4.213842000	-2.284457000
1	-0.764208000	-4.640693000	-1.271886000
1	0.678659000	-5.673801000	-1.281064000
6	2.964269000	-3.679394000	-0.081197000
1	3.293561000	-3.283748000	-1.052934000
1	3.340685000	-4.710906000	-0.012085000
1	3.465435000	-3.101536000	0.709287000
1	0.579308000	1.450416000	-0.758532000
1	0.585495000	1.413438000	0.855045000

One proton added at N5 of **2** (Figure S17-a)
E^{el}_{M06} = -2896.963317
ZPE_{BP86} = 0.92902
E (E^{el}_{M06} + ZPE_{BP86}) = -2896.034297

14	-1.137436000	-0.031646000	-0.135153000
6	-3.399943000	-0.025215000	-0.126136000
6	-4.874865000	-0.006936000	-0.111325000
6	-5.561703000	-0.075707000	1.109106000
1	-5.005186000	-0.131800000	2.044595000
6	-6.956607000	-0.043920000	1.117489000
1	-7.490956000	-0.089266000	2.066441000
6	-7.664699000	0.047910000	-0.084559000
1	-8.754466000	0.069562000	-0.073798000
6	-6.977539000	0.112007000	-1.300041000
1	-7.527785000	0.179660000	-2.238494000
6	-5.581920000	0.089367000	-1.318070000
1	-5.041973000	0.130008000	-2.264122000
7	-2.581393000	1.050418000	-0.201926000
6	-2.852058000	2.507357000	-0.267174000
6	-3.297139000	2.880622000	-1.689890000
1	-3.427800000	3.968298000	-1.767760000
1	-4.254789000	2.405898000	-1.939887000
1	-2.546845000	2.572667000	-2.430328000
6	-3.915896000	2.929617000	0.755569000
1	-3.969862000	4.026123000	0.780239000
1	-3.654716000	2.579293000	1.763349000
1	-4.911380000	2.550222000	0.498578000
6	-1.513552000	3.174685000	0.068857000
1	-1.603468000	4.262990000	-0.037731000
1	-0.705515000	2.827278000	-0.591327000
1	-1.210201000	2.954721000	1.101372000
7	-2.598742000	-1.105588000	-0.065598000
6	-2.887361000	-2.559197000	-0.013501000
6	-1.520764000	-3.252754000	-0.106499000
1	-0.989902000	-2.974119000	-1.026482000
1	-1.652197000	-4.341564000	-0.097730000
1	-0.890554000	-2.977791000	0.751252000
6	-3.770652000	-2.972730000	-1.199329000
1	-4.767260000	-2.518978000	-1.138174000
1	-3.895511000	-4.063654000	-1.201458000
1	-3.307061000	-2.677732000	-2.150396000
6	-3.555354000	-2.919385000	1.322149000
1	-2.938286000	-2.583156000	2.166930000

1	-3.671560000	-4.008964000	1.396186000
1	-4.551136000	-2.467906000	1.408172000
7	0.072875000	-0.185678000	-1.307412000
6	0.142316000	-0.086422000	-2.774015000
6	0.323054000	1.385225000	-3.178663000
1	0.429910000	1.487674000	-4.267666000
1	-0.544701000	1.980029000	-2.860988000
1	1.210950000	1.806722000	-2.691006000
6	1.343608000	-0.906596000	-3.258014000
1	1.426530000	-0.874999000	-4.352418000
1	2.277251000	-0.497713000	-2.842264000
1	1.254343000	-1.955211000	-2.943221000
6	-1.156106000	-0.644333000	-3.371754000
1	-1.154751000	-0.556555000	-4.466482000
1	-1.281808000	-1.704557000	-3.111204000
1	-2.026458000	-0.085020000	-2.993260000
7	-0.015759000	-0.077351000	1.131199000
14	-0.072795000	-0.032767000	2.879032000
6	0.407635000	-1.721444000	3.548626000
1	0.358435000	-1.749889000	4.646927000
1	-0.260795000	-2.508486000	3.167405000
1	1.437172000	-1.978572000	3.258267000
6	1.125416000	1.275707000	3.477468000
1	1.146719000	1.337314000	4.574936000
1	2.147703000	1.058001000	3.134756000
1	0.851694000	2.263733000	3.081669000
6	-1.843747000	0.386836000	3.351329000
1	-1.969015000	0.417370000	4.442878000
1	-2.134158000	1.370214000	2.954627000
1	-2.546927000	-0.363119000	2.958720000
14	1.310649000	-0.331426000	-0.028102000
7	1.974619000	-1.871403000	0.311018000
14	3.135066000	-3.089463000	-0.242825000
6	4.314490000	-2.442437000	-1.537322000
1	4.912786000	-3.289391000	-1.906636000
1	3.802900000	-2.002734000	-2.402314000
1	5.008425000	-1.698980000	-1.130164000
6	2.112773000	-4.505156000	-0.950621000
1	2.753735000	-5.343290000	-1.261719000
1	1.401913000	-4.895138000	-0.206327000
1	1.535200000	-4.182793000	-1.829598000
6	4.071576000	-3.697716000	1.265658000
1	4.759007000	-4.515819000	1.004789000
1	4.665257000	-2.887062000	1.709821000

1	3.384799000	-4.077066000	2.037176000
7	2.049563000	2.210568000	-0.070408000
6	2.740702000	3.519486000	0.062967000
6	4.038974000	3.524440000	0.890275000
1	4.316225000	4.566077000	1.104767000
1	3.889784000	3.013684000	1.851713000
1	4.883224000	3.053998000	0.377715000
6	1.720824000	4.437548000	0.762630000
1	2.132668000	5.449580000	0.875624000
1	0.794994000	4.498552000	0.179365000
1	1.477104000	4.053159000	1.762845000
6	2.989327000	4.052678000	-1.360366000
1	3.336827000	5.094051000	-1.309157000
1	3.754265000	3.461599000	-1.879139000
1	2.063381000	4.027663000	-1.951374000
6	2.608613000	1.052282000	-0.062758000
6	4.057093000	0.706455000	-0.028871000
6	4.605731000	0.063535000	1.091042000
1	3.963829000	-0.179010000	1.938745000
6	5.966352000	-0.242823000	1.132047000
1	6.389174000	-0.718046000	2.017937000
6	6.786917000	0.054200000	0.038768000
1	7.848697000	-0.190812000	0.069674000
6	6.237322000	0.650872000	-1.100116000
1	6.867171000	0.863635000	-1.964414000
6	4.879894000	0.973619000	-1.134596000
1	4.450542000	1.432051000	-2.025646000
1	1.293196000	-2.355214000	0.903621000

Two protons added at N5 of **2** (Figure S17-b)
E^{el}_{M06} = -2897.253996
ZPE_{BP86} = 0.9419
E (E^{el}_{M06} + ZPE_{BP86}) = -2896.312096

14	-1.142175000	-0.113392000	-0.117543000
6	-3.381012000	0.005147000	-0.064514000
6	-4.844086000	0.088887000	0.002686000
6	-5.505459000	-0.230569000	1.200121000
1	-4.931236000	-0.523690000	2.078504000
6	-6.894127000	-0.122892000	1.268050000
1	-7.407934000	-0.350533000	2.201835000
6	-7.623455000	0.275536000	0.143000000
1	-8.709627000	0.348035000	0.197510000
6	-6.963872000	0.581857000	-1.051461000
1	-7.533491000	0.884142000	-1.930056000
6	-5.573040000	0.504130000	-1.123641000

1	-5.055860000	0.732757000	-2.055145000
7	-2.512540000	1.044408000	-0.168414000
6	-2.703136000	2.510706000	0.069400000
6	-3.337928000	3.167241000	-1.164277000
1	-3.380427000	4.254250000	-1.016249000
1	-4.362834000	2.811013000	-1.320999000
1	-2.751680000	2.972748000	-2.071403000
6	-3.569274000	2.762124000	1.310288000
1	-3.592949000	3.840677000	1.513626000
1	-3.149711000	2.264637000	2.195207000
1	-4.602862000	2.426134000	1.171791000
6	-1.292007000	3.062327000	0.300031000
1	-1.331951000	4.152994000	0.404939000
1	-0.621725000	2.826472000	-0.536771000
1	-0.848303000	2.649379000	1.216047000
7	-2.623331000	-1.120711000	-0.020717000
6	-2.986176000	-2.557760000	-0.196554000
6	-1.644599000	-3.287412000	-0.333676000
1	-1.080617000	-2.911748000	-1.196781000
1	-1.813935000	-4.361947000	-0.472902000
1	-1.037940000	-3.152912000	0.575534000
6	-3.829338000	-2.743165000	-1.465268000
1	-4.794697000	-2.229227000	-1.382037000
1	-4.032320000	-3.811003000	-1.619209000
1	-3.302082000	-2.364992000	-2.350941000
6	-3.734850000	-3.090537000	1.032519000
1	-3.165868000	-2.909476000	1.954358000
1	-3.874920000	-4.174660000	0.928989000
1	-4.726038000	-2.634968000	1.130954000
7	0.094346000	-0.249047000	-1.316578000
6	0.111254000	0.025269000	-2.782566000
6	-0.199187000	1.506935000	-3.028393000
1	-0.167310000	1.737563000	-4.101460000
1	-1.204738000	1.752019000	-2.660869000
1	0.528705000	2.139002000	-2.502833000
6	1.506075000	-0.304010000	-3.319687000
1	1.571420000	-0.072268000	-4.390281000
1	2.268667000	0.298500000	-2.803247000
1	1.738422000	-1.369241000	-3.194772000
6	-0.946305000	-0.856322000	-3.454769000
1	-0.956678000	-0.688498000	-4.539550000
1	-0.751648000	-1.922883000	-3.275731000
1	-1.950889000	-0.617266000	-3.074017000
7	0.034296000	-0.152540000	1.129441000
14	-0.026402000	-0.153775000	2.901653000
6	0.962867000	-1.640660000	3.493430000
1	0.887638000	-1.737833000	4.586581000
1	0.577178000	-2.580600000	3.067212000
1	2.036339000	-1.565599000	3.263755000
6	0.709992000	1.443478000	3.536687000
1	0.794923000	1.425648000	4.633087000

1	1.713648000	1.634510000	3.130279000
1	0.081328000	2.304924000	3.269776000
6	-1.822268000	-0.331267000	3.392479000
1	-1.918681000	-0.348165000	4.487585000
1	-2.427464000	0.510547000	3.027242000
1	-2.258785000	-1.262185000	3.005622000
14	1.305587000	-0.195788000	-0.071270000
7	2.204804000	-1.803807000	0.296661000
14	2.999334000	-3.120360000	-0.819490000
6	4.272139000	-2.272515000	-1.871476000
1	4.722592000	-3.030143000	-2.531549000
1	3.865597000	-1.480873000	-2.511135000
1	5.078319000	-1.848251000	-1.258863000
6	1.590761000	-3.906675000	-1.747345000
1	1.999212000	-4.643517000	-2.455162000
1	0.914538000	-4.445298000	-1.069721000
1	1.001809000	-3.183533000	-2.325581000
6	3.767703000	-4.258412000	0.440236000
1	4.266234000	-5.094620000	-0.072753000
1	4.532801000	-3.744776000	1.040875000
1	3.025656000	-4.695721000	1.123880000
7	2.215431000	2.203168000	-0.540131000
6	2.922437000	3.503584000	-0.617363000
6	4.015743000	3.735240000	0.437173000
1	4.298978000	4.796083000	0.424897000
1	3.644947000	3.503704000	1.445792000
1	4.923733000	3.150776000	0.258764000
6	1.822595000	4.567494000	-0.442220000
1	2.249022000	5.571725000	-0.561974000
1	1.030721000	4.433613000	-1.190045000
1	1.375972000	4.502273000	0.559242000
6	3.489635000	3.603758000	-2.048336000
1	3.897425000	4.611126000	-2.205809000
1	4.298023000	2.879834000	-2.212295000
1	2.704654000	3.430624000	-2.796402000
6	2.673122000	1.101849000	-0.064702000
6	4.014138000	0.711403000	0.462658000
6	4.126009000	0.208334000	1.773474000
1	3.261899000	0.242708000	2.442075000
6	5.350607000	-0.261109000	2.257112000
1	5.428984000	-0.616396000	3.284978000
6	6.476536000	-0.254106000	1.427553000
1	7.433423000	-0.618018000	1.801406000
6	6.372090000	0.231187000	0.120281000
1	7.248325000	0.242645000	-0.528620000
6	5.150084000	0.705782000	-0.361848000
1	5.074208000	1.068151000	-1.386636000
1	2.990786000	-1.486747000	0.901775000
1	1.577219000	-2.314640000	0.934457000

W(CO)5 adduct at N4 of **1** (Figure S18-a).

E^{el}_{M06} = -3994.327183

ZPE_{BP86} = 1.081227

E (E^{el}_{M06} + ZPE_{BP86}) = -3993.245956

14	-1.064445000	0.975727000	0.273780000
6	-3.102775000	0.352593000	2.583536000
7	-2.673044000	0.531587000	1.177466000
14	1.697278000	-0.206775000	0.234723000
6	-1.935721000	0.846075000	3.447241000
1	-2.134853000	0.633487000	4.506245000
1	-1.789799000	1.924471000	3.322474000
1	-1.005756000	0.342548000	3.167068000
7	-2.503656000	1.007325000	-0.917970000
6	-3.348835000	-1.141082000	2.851273000
1	-4.162364000	-1.527558000	2.224044000
1	-3.625340000	-1.296473000	3.904115000
1	-2.446617000	-1.726554000	2.646222000
6	-3.392397000	0.797034000	0.071330000
7	2.574492000	0.580191000	-1.209359000
6	-4.350864000	1.165692000	2.970483000
1	-5.271637000	0.784059000	2.518118000
1	-4.237416000	2.222003000	2.695297000
1	-4.467453000	1.113362000	4.062110000
7	0.019919000	-0.364819000	0.089100000
7	2.381496000	1.421964000	0.783825000
6	-4.873779000	0.920498000	-0.008043000
6	-5.460501000	2.178001000	-0.218458000
1	-4.829603000	3.055393000	-0.318953000
6	-7.673191000	1.208784000	-0.051133000
1	-8.758096000	1.319451000	-0.069682000
6	-6.848392000	2.320396000	-0.236375000
1	-7.283033000	3.309236000	-0.387675000
6	-7.096592000	-0.044605000	0.168653000
1	-7.727349000	-0.920846000	0.322195000
6	-2.670216000	1.337979000	-2.346801000
6	-5.709449000	-0.187309000	0.200973000
1	-5.274669000	-1.164737000	0.377392000
6	-3.212132000	2.761442000	-2.571297000
1	-2.628510000	3.488583000	-1.992656000
1	-4.269398000	2.845791000	-2.298789000
1	-3.121175000	3.017191000	-3.636802000
6	-1.249439000	1.295405000	-2.916685000
1	-0.788839000	0.320786000	-2.725325000
1	-0.639513000	2.067345000	-2.425477000
1	-1.255346000	1.476883000	-3.998839000
6	-3.573158000	0.313854000	-3.046183000
1	-3.658179000	0.561544000	-4.113506000
1	-4.581678000	0.314317000	-2.612936000
1	-3.159893000	-0.696440000	-2.960719000

6	2.782542000	0.434900000	-2.677277000
6	1.983471000	-0.794289000	-3.097682000
1	2.205129000	-1.660442000	-2.464259000
1	2.199974000	-1.059315000	-4.140127000
1	0.915261000	-0.586353000	-3.009908000
6	4.273873000	0.235673000	-2.998471000
1	4.685031000	-0.647077000	-2.499506000
1	4.861461000	1.114761000	-2.704578000
1	4.391062000	0.102305000	-4.083230000
6	2.266512000	1.643789000	-3.479846000
1	2.178031000	1.350119000	-4.535179000
1	2.946489000	2.500342000	-3.426235000
1	1.277796000	1.957329000	-3.129399000
6	2.835440000	1.666551000	-0.445232000
6	3.387958000	2.951521000	-0.927910000
6	2.451141000	3.943646000	-1.247856000
1	1.391851000	3.729530000	-1.099808000
6	2.886110000	5.178221000	-1.723409000
1	2.153312000	5.948178000	-1.966194000
6	4.254455000	5.429724000	-1.872107000
1	4.594502000	6.398465000	-2.240164000
6	5.187175000	4.443448000	-1.539821000
1	6.254549000	4.641037000	-1.643484000
6	4.757150000	3.199442000	-1.069586000
1	5.485526000	2.435259000	-0.800054000
6	2.681093000	2.123770000	2.056992000
6	4.078957000	1.712568000	2.543212000
1	4.151107000	0.622780000	2.642164000
1	4.277182000	2.166788000	3.524371000
1	4.851907000	2.055973000	1.842912000
6	1.627170000	1.653553000	3.058856000
1	0.627580000	1.883270000	2.673827000
1	1.773624000	2.150963000	4.026511000
1	1.714972000	0.572700000	3.220353000
6	2.627504000	3.649819000	1.918460000
1	1.701110000	3.959524000	1.428007000
1	3.480148000	4.046060000	1.355654000
1	2.655937000	4.092412000	2.923643000
7	2.675125000	-1.447705000	0.962409000
14	2.152854000	-2.314637000	2.450012000
6	2.133086000	-4.172519000	2.149101000
1	2.233053000	-4.696736000	3.111769000
1	2.944715000	-4.509904000	1.493166000
1	1.191097000	-4.497788000	1.693480000
6	0.471370000	-1.750230000	3.063689000
1	-0.012508000	-2.599578000	3.565946000
1	-0.186700000	-1.400428000	2.262678000
1	0.579168000	-0.951311000	3.806565000
6	3.261390000	-1.947613000	3.939513000
1	2.670040000	-2.202521000	4.833523000
1	3.513608000	-0.880887000	4.018673000

1	4.190051000	-2.524359000	3.984755000
14	4.350567000	-1.817559000	0.428414000
6	5.465909000	-0.313130000	0.199369000
1	6.375476000	-0.663630000	-0.312730000
1	5.770207000	0.107777000	1.164545000
1	5.034026000	0.482132000	-0.409517000
6	5.338286000	-2.818729000	1.690980000
1	4.863051000	-3.734695000	2.058686000
1	5.653408000	-2.214638000	2.550028000
1	6.250754000	-3.119841000	1.151178000
6	4.454365000	-2.865730000	-1.130212000
1	4.214417000	-3.911896000	-0.905724000
1	5.499183000	-2.827767000	-1.476998000
1	3.814916000	-2.559964000	-1.962679000
7	-0.470236000	2.464233000	0.587350000
14	-1.057587000	3.982280000	1.058405000
6	-0.731456000	4.433611000	2.879986000
1	-1.418235000	3.906598000	3.558597000
1	-0.906902000	5.511764000	3.022544000
1	0.290273000	4.216040000	3.211501000
6	-0.304993000	5.399447000	0.036490000
1	-0.430337000	5.220130000	-1.043145000
1	0.763797000	5.562721000	0.230524000
1	-0.827026000	6.338745000	0.276759000
6	-2.925110000	4.285813000	0.886163000
1	-3.235318000	4.345905000	-0.165683000
1	-3.191917000	5.242555000	1.361711000
1	-3.515025000	3.500403000	1.375367000
74	-0.786940000	-2.467543000	-0.681453000
6	1.003808000	-3.433909000	-0.902588000
8	1.826858000	-4.222907000	-1.129037000
6	-0.831867000	-2.118028000	-2.701804000
8	-0.979513000	-2.173074000	-3.855291000
6	-2.796733000	-2.172958000	-0.714443000
8	-3.945101000	-2.315412000	-0.846312000
6	-1.113475000	-3.386006000	1.122281000
8	-1.433839000	-4.102459000	1.981389000
6	-1.400853000	-4.238422000	-1.314253000
8	-1.758626000	-5.284538000	-1.685530000

$W(CO)_5$ adduct at N3 of **1** (Figure S18-b)

E^{el}_{M06} = -3994.340137

ZPE_{BP86} = 1.078669

E (E^{el}_{M06} + ZPE_{BP86}) = -3993.261468

14	-1.540906000	-0.140749000	0.038056000
6	-3.454748000	0.915079000	-2.189221000

7	-3.036519000	0.637888000	-0.800907000
14	1.264569000	1.570134000	-0.085897000
6	-2.314136000	0.364762000	-3.058397000
1	-2.467681000	0.626907000	-4.113124000
1	-2.269829000	-0.728603000	-2.971976000
1	-1.344680000	0.761285000	-2.732497000
7	-2.744219000	0.522473000	1.330584000
6	-3.665069000	2.420456000	-2.408960000
1	-4.536956000	2.780681000	-1.847101000
1	-3.840056000	2.622138000	-3.475146000
1	-2.787875000	2.986352000	-2.086880000
6	-3.584373000	0.964415000	0.383484000
7	2.530438000	1.334167000	1.274885000
6	-4.745502000	0.173585000	-2.579806000
1	-5.607154000	0.519692000	-1.996161000
1	-4.631899000	-0.908458000	-2.450608000
1	-4.960328000	0.367050000	-3.640592000
7	-0.164865000	0.782892000	-0.066625000
7	2.718049000	0.663329000	-0.772031000
6	-4.838290000	1.729631000	0.622489000
6	-6.108564000	1.164961000	0.436710000
1	-6.199959000	0.125174000	0.131397000
6	-7.152821000	3.242648000	1.120934000
1	-8.051990000	3.829125000	1.313188000
6	-7.256591000	1.918184000	0.688375000
1	-8.237409000	1.461913000	0.549969000
6	-5.889617000	3.808123000	1.313808000
1	-5.795744000	4.839496000	1.655957000
6	-2.853741000	0.480914000	2.801310000
6	-4.739915000	3.055359000	1.074002000
1	-3.756602000	3.494220000	1.223851000
6	-1.942709000	-0.675481000	3.243922000
1	-0.916740000	-0.530007000	2.882693000
1	-2.300918000	-1.628489000	2.834298000
1	-1.916985000	-0.742903000	4.339598000
6	-2.362831000	1.814335000	3.391858000
1	-3.079129000	2.617843000	3.177053000
1	-1.395140000	2.089086000	2.961919000
1	-2.254909000	1.734235000	4.483005000
6	-4.280664000	0.205857000	3.302521000
1	-4.251375000	0.094139000	4.395548000
1	-4.681894000	-0.723261000	2.880066000
1	-4.971182000	1.023688000	3.066889000
6	2.594600000	1.610451000	2.731102000

6	1.137084000	1.628175000	3.202215000
1	0.571496000	2.381113000	2.633893000
1	1.080342000	1.888646000	4.267304000
1	0.664846000	0.654260000	3.047898000
6	3.184979000	3.011243000	2.976055000
1	2.668617000	3.770481000	2.375365000
1	4.257830000	3.039532000	2.746097000
1	3.067692000	3.274392000	4.036755000
6	3.407011000	0.590381000	3.542776000
1	3.263228000	0.815805000	4.609016000
1	4.479887000	0.661035000	3.331287000
1	3.079806000	-0.435651000	3.360154000
6	3.323452000	0.646450000	0.434102000
6	4.576878000	-0.074944000	0.757112000
6	4.562273000	-1.478023000	0.736238000
1	3.649798000	-2.008725000	0.478084000
6	5.712939000	-2.195900000	1.060765000
1	5.680494000	-3.285325000	1.043937000
6	6.890335000	-1.521068000	1.394268000
1	7.791878000	-2.083026000	1.641195000
6	6.910133000	-0.123255000	1.410253000
1	7.826213000	0.409517000	1.667848000
6	5.756903000	0.599985000	1.102311000
1	5.775426000	1.687689000	1.120795000
6	3.181840000	0.260857000	-2.126084000
6	4.488374000	0.965094000	-2.526388000
1	4.360048000	2.051031000	-2.603569000
1	4.802555000	0.592959000	-3.511824000
1	5.297310000	0.745490000	-1.816235000
6	2.058815000	0.720048000	-3.066768000
1	1.099733000	0.275177000	-2.777546000
1	2.278778000	0.421094000	-4.099171000
1	1.958746000	1.813431000	-3.033364000
6	3.403298000	-1.254088000	-2.238326000
1	2.593780000	-1.811267000	-1.758301000
1	4.349768000	-1.553378000	-1.773713000
1	3.439637000	-1.542806000	-3.297482000
7	1.342882000	3.254465000	-0.531284000
14	-0.128466000	4.063661000	-1.123669000
6	-0.033760000	5.952804000	-1.196206000
1	-1.002131000	6.260216000	-1.624653000
1	0.748035000	6.376463000	-1.836227000
1	0.042268000	6.414686000	-0.204030000
6	-1.519010000	3.784605000	0.095385000

1	-1.215022000	4.216231000	1.061634000
1	-1.723198000	2.722336000	0.245618000
1	-2.429039000	4.302510000	-0.235947000
6	-0.417035000	3.528507000	-2.902001000
1	-1.288218000	4.021793000	-3.354348000
1	-0.543345000	2.445913000	-3.002278000
1	0.467470000	3.813175000	-3.490975000
14	2.793126000	4.272553000	-0.685951000
6	4.452153000	3.541948000	-0.196834000
1	5.192812000	4.307129000	-0.480649000
1	4.732062000	2.616434000	-0.701149000
1	4.529117000	3.400323000	0.883226000
6	3.023505000	4.775202000	-2.492754000
1	2.166780000	5.304379000	-2.925594000
1	3.216047000	3.891905000	-3.119626000
1	3.899576000	5.436341000	-2.577676000
6	2.639452000	5.800753000	0.429178000
1	2.428216000	6.718113000	-0.134808000
1	3.581473000	5.955917000	0.975045000
1	1.840554000	5.679546000	1.173147000
7	-1.621502000	-1.812688000	-0.072866000
14	-3.018448000	-2.840088000	-0.204878000
6	-3.001307000	-4.302039000	0.998907000
1	-2.338035000	-5.127853000	0.719484000
1	-4.024120000	-4.708267000	1.050479000
1	-2.725492000	-3.980142000	2.013793000
6	-4.694241000	-2.064043000	0.222542000
1	-4.941488000	-1.216216000	-0.419170000
1	-4.765936000	-1.750468000	1.270840000
1	-5.459998000	-2.837823000	0.056689000
6	-3.227126000	-3.475441000	-1.984962000
1	-2.340462000	-3.281135000	-2.601930000
1	-4.085002000	-2.990316000	-2.474445000
1	-3.409850000	-4.559473000	-2.006753000
74	0.270734000	-3.126098000	-0.275428000
6	-0.483987000	-4.803530000	-1.170445000
8	-0.798134000	-5.831215000	-1.613986000
6	0.314052000	-2.322158000	-2.172955000
8	0.246368000	-2.001735000	-3.286915000
6	2.039669000	-3.997495000	-0.505780000
8	3.107147000	-4.469966000	-0.628359000
6	1.125494000	-1.828474000	1.039155000
8	1.582929000	-1.331193000	1.988088000
6	0.077053000	-4.170854000	1.469709000

8	0.051384000	-4.807911000	2.441053000

W(CO)5 adduct at N4 of **1** (Figure S18-c).
E^{el}_{M06} = -3994.347925
ZPE_{BP86} = 1.07924
E (E^{el}_{M06} + ZPE_{BP86}) = -3993.268685

14	-0.841016000	0.469472000	0.362577000
6	-1.892322000	1.777983000	-2.342570000
7	-1.837719000	1.511226000	-0.871304000
14	2.384463000	0.677267000	0.044225000
6	-0.645420000	1.110837000	-2.917623000
1	-0.632120000	1.201936000	-4.011518000
1	-0.613196000	0.046149000	-2.663372000
1	0.247390000	1.594755000	-2.507008000
7	-1.837259000	1.792911000	1.290526000
6	-1.863495000	3.269441000	-2.734077000
1	-2.811680000	3.777361000	-2.533021000
1	-1.686863000	3.321000000	-3.817911000
1	-1.057854000	3.816934000	-2.236515000
6	-2.326075000	2.263389000	0.130464000
7	3.471170000	-0.476019000	1.042659000
6	-3.157377000	1.160421000	-2.958590000
1	-4.058597000	1.658637000	-2.582852000
1	-3.238369000	0.092189000	-2.741768000
1	-3.125738000	1.289663000	-4.049664000
7	0.783453000	0.794606000	0.276660000
7	3.059031000	-0.656351000	-1.073781000
6	-3.141849000	3.488313000	-0.040027000
6	-4.459891000	3.451417000	-0.510241000
1	-4.935582000	2.492726000	-0.705874000
6	-4.550949000	5.872543000	-0.431842000
1	-5.100334000	6.801503000	-0.589258000
6	-5.162119000	4.643575000	-0.697356000
1	-6.192009000	4.610017000	-1.054267000
6	-3.235380000	5.908630000	0.040957000
1	-2.754171000	6.864362000	0.251947000
6	-2.437662000	1.883556000	2.654476000
6	-2.535847000	4.720307000	0.246152000
1	-1.509831000	4.733831000	0.612657000
6	-3.157902000	0.555397000	2.951580000
1	-2.486022000	-0.300064000	2.842436000
1	-3.991468000	0.408438000	2.252755000
1	-3.559762000	0.569017000	3.974383000

6	-1.296421000	2.119850000	3.651200000
1	-0.880399000	3.127556000	3.528181000
1	-0.487147000	1.396704000	3.511796000
1	-1.670945000	2.021109000	4.679652000
6	-3.460638000	3.014516000	2.830910000
1	-3.810243000	2.977303000	3.872145000
1	-4.334892000	2.893865000	2.180995000
1	-3.027817000	4.006133000	2.655595000
6	4.246380000	-0.414081000	2.309360000
6	3.681262000	0.766912000	3.105774000
1	3.807595000	1.700209000	2.543565000
1	4.216284000	0.860976000	4.059921000
1	2.615261000	0.629598000	3.323101000
6	5.734729000	-0.146252000	2.029572000
1	5.860172000	0.791433000	1.472936000
1	6.193516000	-0.964694000	1.461229000
1	6.277371000	-0.051869000	2.980613000
6	4.084632000	-1.714007000	3.113749000
1	4.503885000	-1.577477000	4.120485000
1	4.609989000	-2.550700000	2.638608000
1	3.024571000	-1.975316000	3.212129000
6	3.716330000	-1.228005000	-0.048733000
6	4.564574000	-2.450399000	-0.111400000
6	4.088309000	-3.665794000	0.400856000
1	3.098645000	-3.710179000	0.848742000
6	4.867795000	-4.820625000	0.312027000
1	4.476302000	-5.760531000	0.702355000
6	6.136248000	-4.772476000	-0.270167000
1	6.745365000	-5.674695000	-0.335190000
6	6.619185000	-3.560910000	-0.771841000
1	7.607976000	-3.511118000	-1.229068000
6	5.837213000	-2.408376000	-0.701269000
1	6.212124000	-1.475491000	-1.115212000
6	2.625524000	-1.204269000	-2.383087000
6	3.623437000	-2.189253000	-3.007563000
1	4.625723000	-1.751182000	-3.104630000
1	3.263111000	-2.436708000	-4.015866000
1	3.701563000	-3.124340000	-2.441992000
6	2.502549000	0.009248000	-3.317887000
1	1.956581000	0.827196000	-2.835456000
1	1.963842000	-0.268783000	-4.232747000
1	3.494712000	0.384537000	-3.601936000
6	1.274667000	-1.912517000	-2.190094000
1	0.580323000	-1.296651000	-1.606300000

1	1.415928000	-2.853138000	-1.643109000
1	0.815426000	-2.142993000	-3.159959000
7	3.226122000	2.185866000	-0.125553000
14	2.303837000	3.616509000	0.428565000
6	3.379076000	5.164660000	0.570165000
1	2.690409000	5.969073000	0.875543000
1	3.864928000	5.490746000	-0.357450000
1	4.144734000	5.078613000	1.351544000
6	1.597347000	3.399234000	2.150319000
1	2.376244000	3.445543000	2.923521000
1	1.051818000	2.456255000	2.244011000
1	0.891666000	4.221750000	2.344532000
6	0.951942000	3.997817000	-0.803586000
1	0.464096000	4.960259000	-0.592313000
1	0.204844000	3.198065000	-0.757076000
1	1.347774000	4.037713000	-1.828064000
14	4.726642000	2.528383000	-0.996660000
6	5.666792000	1.010292000	-1.582474000
1	6.548852000	1.378883000	-2.129147000
1	5.106447000	0.344117000	-2.244254000
1	6.034785000	0.433992000	-0.725403000
6	4.323867000	3.540209000	-2.534222000
1	3.809174000	4.482629000	-2.305717000
1	3.670682000	2.960236000	-3.202764000
1	5.241850000	3.785770000	-3.089057000
6	5.987485000	3.416986000	0.101089000
1	5.929514000	4.509664000	0.036735000
1	7.002552000	3.119382000	-0.201877000
1	5.864173000	3.139199000	1.157900000
7	-1.385084000	-1.080968000	0.691202000
14	-0.565606000	-2.045292000	1.878884000
6	0.350984000	-1.018135000	3.181413000
1	0.918843000	-0.194116000	2.733443000
1	1.052529000	-1.666270000	3.726980000
1	-0.346059000	-0.609350000	3.924478000
6	-1.653247000	-3.147006000	2.963944000
1	-2.137282000	-3.979276000	2.441139000
1	-2.432794000	-2.576591000	3.486864000
1	-0.994009000	-3.579766000	3.734622000
6	0.735978000	-3.151469000	1.088982000
1	0.330459000	-3.802122000	0.305074000
1	1.210827000	-3.785017000	1.853995000
1	1.495403000	-2.500880000	0.639715000
74	-3.129280000	-2.145128000	-0.328712000

6	-4.211394000	-2.476466000	1.387032000
8	-4.945918000	-2.707517000	2.256266000
6	-4.320556000	-0.495543000	-0.462121000
8	-5.168752000	0.305488000	-0.500231000
6	-4.634608000	-3.063481000	-1.256822000
8	-5.519287000	-3.591453000	-1.808857000
6	-2.291127000	-2.075350000	-2.197721000
8	-1.925770000	-2.180103000	-3.299541000
6	-2.327393000	-4.030829000	-0.186036000
8	-2.016107000	-5.151120000	-0.168782000

$W(CO)_5$ adduct at N5 of **2** (Figure S18-d)
E^{el}_{M06} = -3530.314308
ZPE_{BP86} = 0.961182
E (E^{el}_{M06} + ZPE_{BP86}) = -3529.353126

14	2.053146000	0.188539000	0.160470000
6	4.267374000	-0.323396000	-0.084125000
6	5.702452000	-0.661544000	-0.192934000
6	6.273226000	-0.939370000	-1.442352000
1	5.656815000	-0.886146000	-2.339532000
6	7.618347000	-1.297215000	-1.522115000
1	8.060611000	-1.519330000	-2.493489000
6	8.395301000	-1.375247000	-0.361225000
1	9.446377000	-1.657500000	-0.427155000
6	7.824990000	-1.094588000	0.883111000
1	8.427631000	-1.155193000	1.789573000
6	6.476784000	-0.740152000	0.971695000
1	6.021814000	-0.528212000	1.939697000
7	3.710971000	0.906362000	-0.193775000
6	4.355190000	2.222950000	-0.434878000
6	5.124967000	2.668548000	0.819252000
1	5.557745000	3.665564000	0.658074000
1	5.945776000	1.973763000	1.041166000
1	4.463251000	2.718753000	1.693032000
6	5.306158000	2.190067000	-1.643272000

1	5.626239000	3.217197000	-1.865579000
1	4.801474000	1.793210000	-2.534175000
1	6.203943000	1.594248000	-1.449338000
6	3.213810000	3.195262000	-0.741651000
1	3.613452000	4.200593000	-0.925603000
1	2.517580000	3.257111000	0.102931000
1	2.654636000	2.873603000	-1.630681000
7	3.273904000	-1.182904000	0.162690000
6	3.252970000	-2.657412000	0.337180000
6	3.623845000	-2.992030000	1.790312000
1	4.654040000	-2.677021000	2.006201000
1	3.556468000	-4.076840000	1.950915000
1	2.944135000	-2.501558000	2.494898000
6	4.219313000	-3.374875000	-0.617713000
1	4.062881000	-3.057038000	-1.657336000
1	4.016877000	-4.453061000	-0.562803000
1	5.270450000	-3.216476000	-0.352450000
6	1.818780000	-3.095009000	0.004862000
1	1.066529000	-2.500482000	0.537503000
1	1.677893000	-4.148578000	0.277000000
1	1.625613000	-2.989595000	-1.070023000
7	1.098963000	0.553703000	1.501922000
6	1.400088000	1.146007000	2.826928000
6	1.549817000	2.670499000	2.676477000
1	1.715040000	3.147698000	3.653319000
1	2.406481000	2.913010000	2.034225000
1	0.646511000	3.098268000	2.227841000
6	0.270597000	0.862305000	3.825590000
1	0.446441000	1.427161000	4.752115000
1	-0.690003000	1.180537000	3.399845000
1	0.214667000	-0.198552000	4.080821000
6	2.723426000	0.565495000	3.351851000
1	2.992406000	1.033408000	4.309416000
1	2.645435000	-0.517257000	3.511322000
1	3.545824000	0.757353000	2.644442000
7	0.743243000	0.129810000	-0.907012000
14	0.849300000	-0.175017000	-2.636113000
6	-0.049409000	-1.714201000	-3.211726000
1	0.330865000	-1.985544000	-4.208904000
1	0.131581000	-2.562948000	-2.540389000
1	-1.131914000	-1.584196000	-3.294501000
6	0.358344000	1.333159000	-3.631720000
1	0.339710000	1.076849000	-4.702223000
1	-0.630413000	1.719378000	-3.363051000
1	1.085611000	2.145713000	-3.491729000
6	2.669760000	-0.501334000	-3.063341000
1	2.743021000	-0.604441000	-4.156412000
1	3.334025000	0.320311000	-2.766705000
1	3.045114000	-1.433317000	-2.620012000
14	-0.424812000	0.299601000	0.505712000
7	-1.361055000	-1.035217000	0.859119000

14	-1.380995000	-2.142939000	2.189403000
6	-2.599595000	-1.562055000	3.508313000
1	-2.649371000	-2.271404000	4.348632000
1	-2.285852000	-0.584016000	3.899917000
1	-3.614305000	-1.442443000	3.108116000
6	0.307653000	-2.391894000	3.045984000
1	0.720777000	-3.372185000	2.771565000
1	1.036834000	-1.624515000	2.771965000
1	0.194392000	-2.383009000	4.140318000
6	-1.761927000	-3.933749000	1.703690000
1	-1.587833000	-4.557617000	2.595652000
1	-2.777340000	-4.140669000	1.352801000
1	-1.061529000	-4.272847000	0.926138000
7	-1.774200000	2.263124000	1.685265000
6	-2.355879000	3.557935000	2.144950000
6	-1.714959000	4.825149000	1.545410000
1	-2.044607000	5.695851000	2.131530000
1	-0.617924000	4.782021000	1.600347000
1	-1.999619000	4.994684000	0.502063000
6	-2.127040000	3.572894000	3.669229000
1	-2.607635000	4.453660000	4.118515000
1	-2.549142000	2.667563000	4.124983000
1	-1.055264000	3.607023000	3.907848000
6	-3.868576000	3.563715000	1.884712000
1	-4.313185000	4.455911000	2.349951000
1	-4.089800000	3.587608000	0.811661000
1	-4.342734000	2.674174000	2.314064000
6	-1.349186000	1.985912000	0.503201000
6	-1.414202000	2.872358000	-0.694403000
6	-0.261997000	3.547227000	-1.117317000
1	0.663215000	3.390517000	-0.569549000
6	-0.305510000	4.432484000	-2.192431000
1	0.596231000	4.968188000	-2.494318000
6	-1.502222000	4.623610000	-2.890231000
1	-1.539327000	5.305191000	-3.740801000
6	-2.647957000	3.924929000	-2.499555000
1	-3.580689000	4.051139000	-3.050366000
6	-2.607928000	3.059770000	-1.404275000
1	-3.503862000	2.519823000	-1.103116000
74	-3.223131000	-1.227497000	-0.503628000
6	-4.774640000	-1.540297000	-1.708563000
8	-5.679839000	-1.720206000	-2.424854000
6	-2.414229000	-2.951376000	-1.286966000
8	-2.076939000	-3.960986000	-1.748602000
6	-2.701942000	-0.084378000	-2.107700000
8	-2.678235000	0.488501000	-3.125062000
6	-4.379410000	-2.361660000	0.756558000
8	-5.197536000	-2.958046000	1.326974000
6	-4.333052000	0.315616000	0.255366000
8	-5.144864000	1.073024000	0.600440000

(S4) Reference:

[1] a) D. Stalke, Chem. *Soc. Rev*. **1998**, *27*, 171-178; b) T. Kottke, D. Stalke, *J. Appl. Crystallogr*. **1993**, *26*, 615-619.

[2] T. Schulz, K. Meindl, D. Leusser, D. Stern, J. Graf, C. Michaelsen, M. Ruf, G. M. Sheldrick, D. Stalke, *J. Appl. Crystallogr*. **2009**, *42*, 885-891.

[3] Bruker AXS Inc., in *Bruker Apex CCD, SAINT v8.30C* (Ed.: Bruker AXS Inst. Inc.), WI, USA, Madison, 2013.

[4] L. Krause, R. Herbst-Irmer, G. M. Sheldrick, D. Stalke, *J. Appl. Crystallogr*. **2015**, *48*, 3-10.

[5] G. M. Sheldrick, *Acta Crystallogr.* **2015**, *A71*, 3-8.

[6] G. M. Sheldrick, *Acta Crystallogr*. **2015**, *C71*, 3-8.

[7] C. B. Hübschle, G. M. Sheldrick, B. Dittrich, *J. Appl. Crystallogr*. **2011**, *44*, 1281-1284.

[8] M. J. Frisch, G. W. Trucks, H. B. Schlegel, G. E. Scuseria, M. A. Robb, J. R. Cheeseman, G. Scalmani, V. Barone, B. Mennucci, G. A. Petersson, H. Nakatsuji, M. Caricato, X. Li, H. P. Hratchian, A. F. Izmaylov, J. Bloino, G. Zheng, J. L. Sonnenberg, M. Hada, M. Ehara, K. Toyota, R. Fukuda, J. Hasegawa, M. Ishida, T. Nakajima, Y. Honda, O. Kitao, H. Nakai, T. Vreven, J. A. Montgomery, Jr.,J. E. Peralta, F. Ogliaro, M. Bearpark, J. J. Heyd, E. Brothers, K. N. Kudin, V. N. Staroverov, T. Keith, R. Kobayashi, J. Normand, K. Raghavachari, A. Rendell, J. C. Burant, S. S. Iyengar, J. Tomasi, M. Cossi, N. Rega, J. M. Millam, M. Klene, J. E. Knox, J. B. Cross, V. Bakken, C. Adamo, J. Jaramillo, R. Gomperts, R. E.Stratmann, O. Yazyev, A. J. Austin, R. Cammi, C. Pomelli, J. W. Ochterski, R. L. Martin, K. Morokuma, V. G. Zakrzewski, G. A. Voth, P. Salvador, J. J. Dannenberg, S. Dapprich, A. D. Daniels, O. Farkas, J. B. Foresman, J. V. Ortiz, J. CioslowskiandD. J. Fox, Gaussian, Inc., Wallingford CT, **2010**.

[9] (a) A. D. Becke, *Phys. Rev. A*., **1988**, *38*, 3098 (b) J. P. Perdew, *Phys. Rev. B.,* **1986**, *33*, 8822.

[10] S. Grimme, S. Ehrlich, L. Goerigk, *J. Comput. Chem*., **2011**, *32*, 1456-1465.

[11] a) F. Weigend, R. Ahlrichs, *Phys. Chem. Chem. Phys.,* **2005**, *7*, 3297 (b) A. Schaefer, H. Horn, R. J. Ahlrichs, *J. Chem. Phys*., **1992**, *97*, 2571.

[12] (a) A. E. Reed, L. A. Curtiss and F. Weinhold, *Chem. Rev*., **1988**, *88*, 899 (b) NBO 6.0. E. D. Glendening, J. K. Badenhoop, A. E. Reed, J. E. Carpenter, J. A. Bohmann, C. M. Morales, C. R. Landis, F. Weinhold (Theoretical Chemistry Institute, University of Wisconsin, Madison, WI, **2013**; http://nbo6.chem.wisc.edu/).

[13] Y. Zhao, D. G, Truhlar, *Theor. Chem. Acc.,* **2008**, *120*, 215.

[14] (a) R. F. W. Bader, Atoms in molecules. A Quantum Theory, Oxford University Press, Oxford, **1990**; (b) C. F. Matta and R. J. Boyd, The Quantum Theory of atoms in Molecules, Wiley-VCH, Weinheim, **2007**.

[15] (a) T. Lu and F. Chen, *J. Comput. Chem.,* **2012**, *33*, 580 (b) T. Lu, F. Chen, *J. Mol. GraphicsModell*., **2012**, *38*, 314.

[16] (a) A. D. Becke, *J. Chem. Phys*., **1993**, *98*, 5638-5652. (b) C. Lee, W. Yang, R. G. Parr, *Phys. Rev. B*., **1988**, 785-789. (c)
B. Miehlich, A. Savin, H. Stoll, H. Preuss, *Chem. Phys. Lett.,* **1989,** 200-206.
